# The pH Robustness of Bacterial Sensing

**DOI:** 10.1128/mbio.01650-22

**Published:** 2022-09-26

**Authors:** Elizabet Monteagudo-Cascales, David Martín-Mora, Wenhao Xu, Victor Sourjik, Miguel A. Matilla, Álvaro Ortega, Tino Krell

**Affiliations:** a Department of Environmental Protection, Estación Experimental del Zaidín, Consejo Superior de Investigaciones Científicas, Granada, Spain; b Max Planck Institute for Terrestrial Microbiology and Center for Synthetic Microbiology (SYNMIKRO), Marburg, Germany; c Department of Biochemistry and Molecular Biology ‘B’ and Immunology, Faculty of Chemistry, University of Murcia, Regional Campus of International Excellence “Campus Mare Nostrum”, Murcia, Spain; University of Pennsylvania; Yale School of Medicine

**Keywords:** signal transduction, receptors, sensor domains, pH robustness, bacterial adaptation, pH, sensing

## Abstract

Bacteria have evolved many different signal transduction systems to sense and respond to changing environmental conditions. Signal integration is mainly achieved by signal recognition at extracytosolic ligand-binding domains (LBDs) of receptors. Hundreds of different LBDs have been reported, and our understanding of their sensing properties is growing. Receptors must function over a range of environmental pH values, but there is little information available on the robustness of sensing as a function of pH. Here, we have used isothermal titration calorimetry to determine the pH dependence of ligand recognition by nine LBDs that cover all major LBD superfamilies, of periplasmic solute-binding proteins, and cytosolic LBDs. We show that periplasmic LBDs recognize ligands over a very broad pH range, frequently stretching over eight pH units. This wide pH range contrasts with a much narrower pH response range of the cytosolic LBDs analyzed. Many LBDs must be dimeric to bind ligands, and analytical ultracentrifugation studies showed that the LBD of the Tar chemoreceptor forms dimers over the entire pH range tested. The pH dependences of Pseudomonas aeruginosa motility and chemotaxis were bell-shaped and centered at pH 7.0. Evidence for pH robustness of signaling *in vivo* was obtained by Förster Resonance Energy Transfer (FRET) measurements of the chemotaxis pathway responses in Escherichia coli. Bacteria have evolved several strategies to cope with extreme pH, such as periplasmic chaperones for protein refolding. The intrinsic pH resistance of periplasmic LBDs appears to be another strategy that permits bacteria to survive under adverse conditions.

## INTRODUCTION

The capacity of bacteria to thrive in a variety of different environments relies on an array of signal transduction systems that sense different environmental parameters. The cellular responses include regulation of transcription, alteration of the levels of second messengers, and control of motility (1, 2). Many of these systems incorporate a transmembrane receptor that interacts with the environment through an extracytoplasmic ligand-binding domain (LBD) and mediates downstream signaling with a cytoplasmic domain. Major families of such receptors include histidine kinases; chemoreceptors; adenylate, diadenylate, and diguanylate cyclases; cAMP, c-di-AMP, and c-di-GMP phosphodiesterases; and protein kinases and phosphatases (2, 3).

Bacteria experience frequent and rapid variations in environmental pH. This is well illustrated by species that transit the gastrointestinal tract, within which, depending on the compartment, the pH ranges from strongly acidic to neutral. Changes in the pH can trigger chemotaxis (4, 5) or the expression of virulence factors in many pathogens, including Salmonella enterica (6), Streptococcus pyogenes (7), Vibrio cholerae (8), and Pectobacterium caratovorum (9). Rapid exposure to the very low pH of the stomach represents an extreme stress that may cause proteins to unfold or misfold.

Studies in Escherichia coli have shown that exposure to low external pH causes a rapid drop in the pH of the periplasm and cytosol. Several molecular processes – the action of proton efflux pumps, the secretion of ammonia, and the stimulation of proton-consuming decarboxylation reactions – rapidly restore a neutral cytosolic pH. In marked contrast, the pH in the periplasm remains low after exposure to an acidic environment (10–12). The periplasmic pH equilibrates with the environmental pH because the outer membrane is highly permeable to pH-active compounds (10).

Bacteria have evolved an enormous number of different LBDs. For example, more than 80 LBD types have been identified just for chemoreceptors (13), and new ones are regularly being discovered (14, 15). Members of the same LBD group are frequently found in many different receptor types, indicating that LBDs are modules that recombine with different signaling domains to evolve proteins with different sensor functionalities. For example, 2 of the most abundant LBD types, PAS and dCache, are found in all major receptor types (16, 17). This notion is supported by studies that construct functional chimeric receptors in which LBDs were recombined with receptors of the same (18, 19) or a different type (20, 21). There is also evidence indicating that a given type of LBD is associated with a particular cellular compartment. For example, PAS domains are primarily cytosolic, whereas Cache domains are nearly always extracytosolic (16).

**FIG 1:**
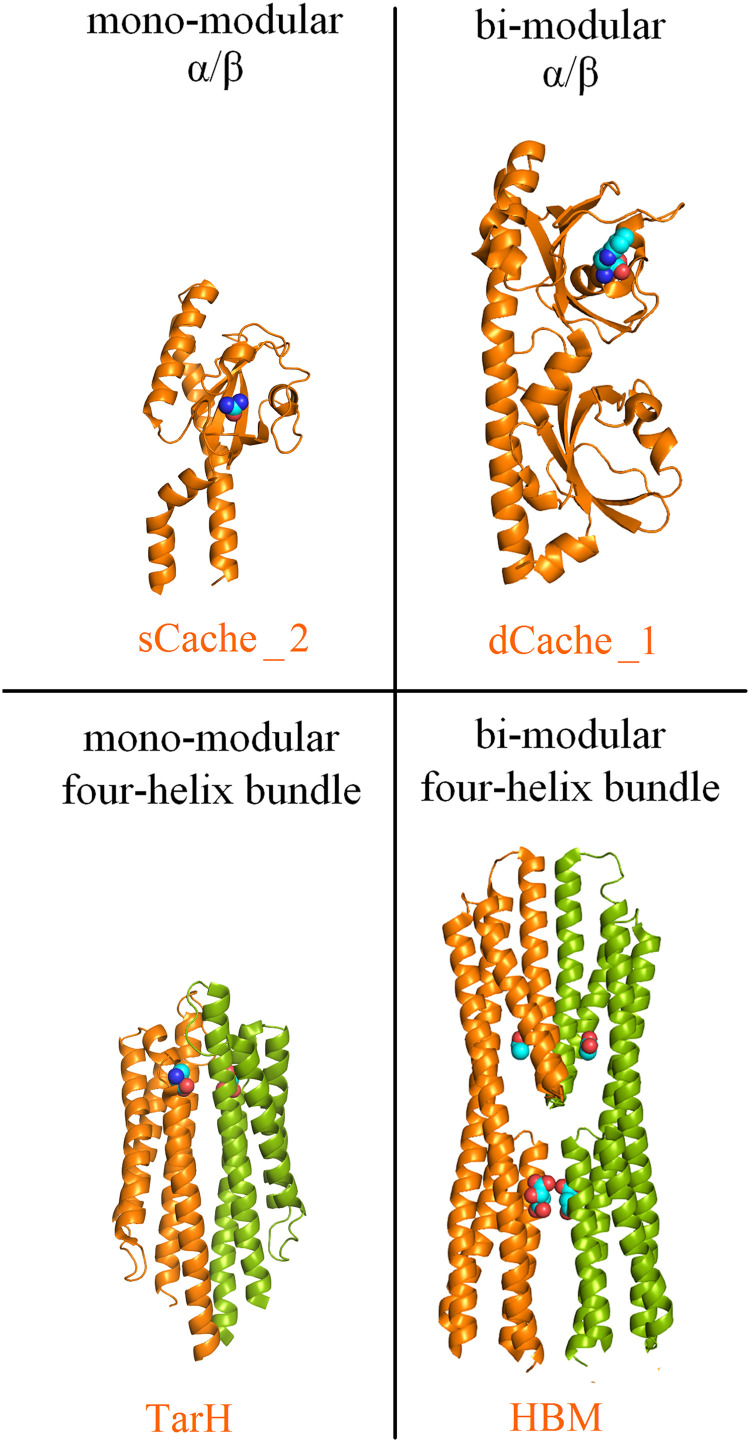
The 4 major structural superfamilies of bacterial ligand-binding domains. Examples of sequence-based LBD superfamilies are shown, and the corresponding Pfam name is provided in orange: the chemoreceptors PscD in complex with acetate (sCache_2) (23), PctA in complex with L-Trp (dCache_1) (24), Tar in complex with L-Asp (TarH) (27) and McpS in complex with malate and acetate (HBM) (28). Bound signal molecules are shown in space-filling mode. Monomers of dimeric LBDs are shown in different colors.

Although sequence-based classification of LBDs, such as that in the Pfam database (22), delineates many LBD types (1, 13, 22), inspection of the increasing number of available LBD 3D structures shows that most belong to 4 different structural superfamilies, namely the mono- and bimodular α/β fold and the mono- and bimodular four-helix bundle fold (1). The structures of representative examples of these 4 structural superfamilies are shown in Fig. 1. Data currently available for sCache and dCache domains, the 2 primary members of the mono- and bimodular α/β domain superfamily, show that ligands bind in a pocket that is formed by a single polypeptide chain (Fig. 1) (23, 24). Individual LBDs of this type are frequently monomeric, and ligand-binding does not alter the state of oligomerization (25, 26). In contrast, members of the mono- and bimodular four-helixbundle superfamily, such as Tar ligand-binding domain homolog (TarH) and helical bimodular (HBM), bind ligands at the dimer interface to establish interactions with both monomers of the dimer (27, 28). Therefore, these domains must dimerize to recognize their ligands, and ligand-binding stabilizes the LBD dimer (29–32). The ability to sense signals under varying environmental conditions implies that these domains must remain associated to recognize their ligands.

Many proteins unfold at extreme pH. A major strategy to counteract the effects of extreme pH in the periplasm is the presence of chaperones that stabilize the native conformation of proteins at low pH (33, 34). The best-studied chaperones are E. coli HdeA and HdeB, which function under highly (pH 1–3) or mildly (pH 4–5) acidic conditions, respectively (33). However, there is little information available about the intrinsic capacity of different receptors to perceive their signals at extreme pH.

In the first part of this report, we address this question by determining the pH dependence of ligand recognition using isothermal titration calorimetry (ITC) to characterize ligand interactions with a selection of proteins (35, 36). In the second part of this report, we investigate signaling output in systems containing the entire receptor protein by studying the pH dependence of chemotaxis responses and Förster Resonance Energy Transfer (FRET) responses of cells containing a single chemoreceptor.

We have studied ligand recognition by periplasmic or cytosolic LBDs and by periplasmic solute-binding proteins (SBPs). Next to their role in providing substrates to different transporter permeases, many SBPs activate different signal transduction receptors by binding to their LBDs (37). A total of 14 different proteins from neutralophilic bacteria that recognize a wide diversity of ligands, including amino acids, purines, polyamines, linear and cyclic organic acids, aromatic compounds, sugars, and auxins, were analyzed. ITC experiments were complemented by analytical ultracentrifugation and differential scanning calorimetry studies to investigate the effect of pH on the oligomeric state and unfolding of the proteins. We show that extracellular LBDs and SBPs recognize their ligands over a very wide pH range, whereas this capacity is reduced for the cytosolic LBDs, suggesting that pH robustness was a major factor in the evolution of transmembrane receptors involved in signal transduction. Robustness of ligand sensing by the periplasmic LBDs of chemoreceptors was confirmed by examining Pseudomonas aeruginosa chemotaxis and analysis of the intracellular chemotaxis signaling pathway using FRET. These results are relevant for the development of biosensors that employ LBDs for analyte sensing.

## RESULTS

To assess the pH dependence of ligand-binding, freshly purified proteins were dialyzed overnight into the buffers described in Table S1 and submitted to microcalorimetric titration. The resulting titration curves are shown in Fig. S1 to S3, and the derived thermodynamic binding parameters are provided in Table S2.

10.1128/mbio.01650-22.1 TABLE S1 Buffers used for microcalorimetric studies. Composition of the dialysis buffers used for each of the proteins analyzed. Dialyzed protein was transferred into the microcalorimeter and ligand solutions were made up in dialysis buffer. Download Table S1, PDF file, 0.09 MB. Copyright © 2022 Monteagudo-Cascales et al. 2022 Monteagudo-Cascales et al. https://creativecommons.org/licenses/by/4.0/ This content is distributed under the terms of the Creative Commons Attribution 4.0 International license.

10.1128/mbio.01650-22.2 TABLE S2 Dissociation constants derived from the microcalorimetric titrations of different proteins with ligands. Highlighted are the pH optima. Download Table S2, PDF file, 0.10 MB. Copyright © 2022 Monteagudo-Cascales et al. 2022 Monteagudo-Cascales et al. https://creativecommons.org/licenses/by/4.0/ This content is distributed under the terms of the Creative Commons Attribution 4.0 International license.

10.1128/mbio.01650-22.5 FIG S1 Microcalorimetric titrations of mono- and bimodular α/β fold ligand-binding domains with ligands at different pH. Upper panels: raw titration data. Lower panels: integrated, dilution heat-corrected and concentration-normalized raw data. The line corresponds to the best fit using the “One-Binding-Site Model” of the MicroCal version of ORIGIN. Download FIG S1, PDF file, 1.3 MB. Copyright © 2022 Monteagudo-Cascales et al. 2022 Monteagudo-Cascales et al. https://creativecommons.org/licenses/by/4.0/ This content is distributed under the terms of the Creative Commons Attribution 4.0 International license.

### Ligand-binding to periplasmic mono- and bimodular α/β ligand-binding domains.

**FIG 2:**
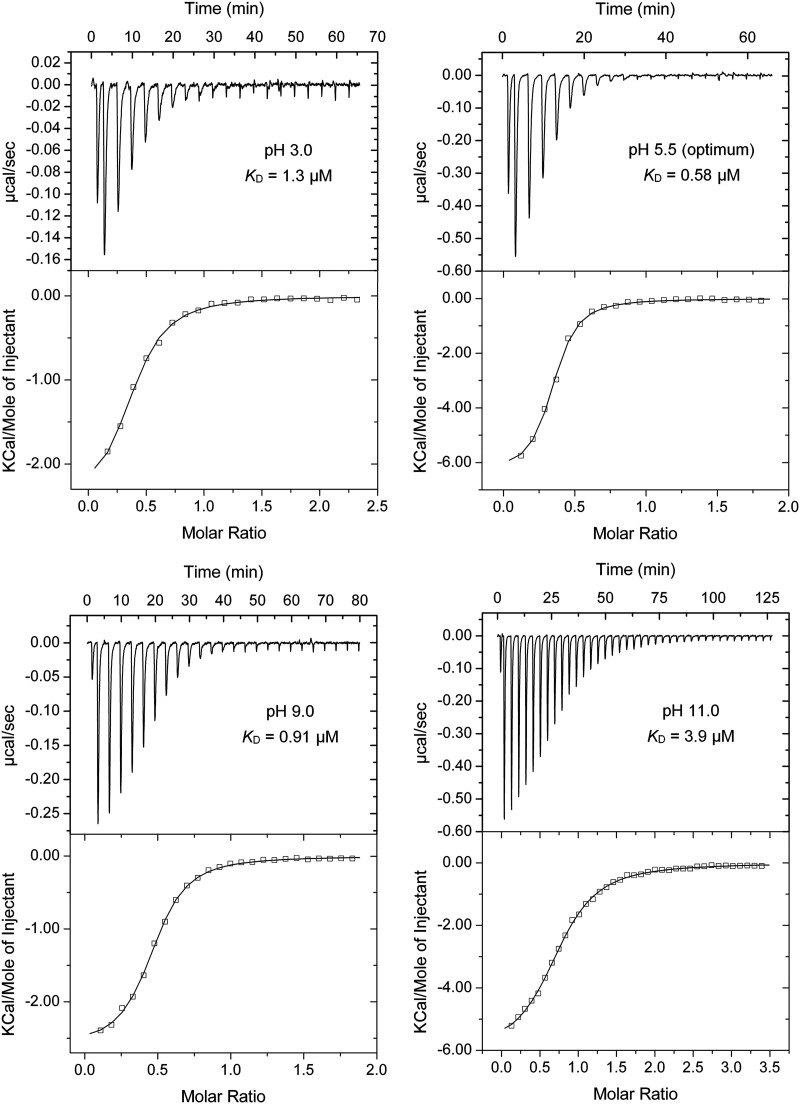
Microcalorimetric titrations of P. aeruginosa PctA-LBD with L-Ala in buffers of different pH. The protein concentration was between 20 and 31 μM and the concentration of L-Ala was 1 mM. Upper panel: raw titration data. The pH and corresponding dissociation constant (*K*_D_) are shown. Lower panel: dilution-heat-corrected and concentration-normalized integrated raw data. Data were fitted with the “One-Binding Site Model” of the MicroCal (Northampton, MA, USA) version of ORIGIN. The derived thermodynamic parameters are shown in Table S2. The dissociation constant pH profile was flat and showed very little variation over the pH range 3.0 to 11.0, for which an average dissociation constant (*K*_D_) of 1.26 ± 0.7 μM was derived. The high affinities at the extreme pH values of 3.0 and 11.0, with *K*_D_ values of 1.3 and 3.9 μM, respectively, are noteworthy (Fig. 2 and Fig. 3A).

**FIG 3:**
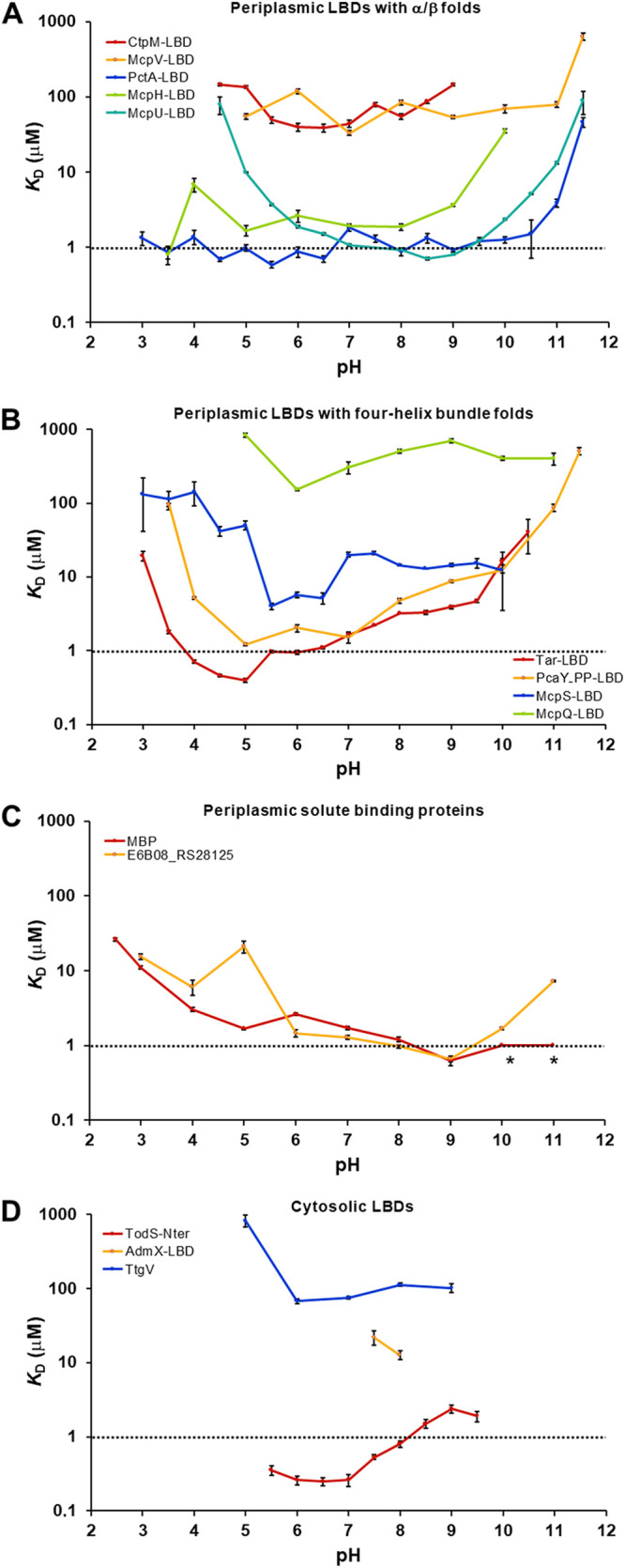
The pH dependence of ligand-binding for different proteins. The dissociation constants (*K*_D_) at different pH values are shown for periplasmic LBDs with α/β folds (A) four-helixbundle folds (B), periplasmic solute-binding proteins (C), and cytosolic LBDs (D). The titration curves and the derived thermodynamic parameters are shown in Fig. S1 to S3 and Table S2. The following ligands were used for the binding studies: CtpM: L-malate, McpV: propionate, PctA: l-alanine, McpH: adenine, McpU: putrescine, Tar: L-aspartate, PcaY_PP: quinate, McpS: L-malate, McpQ: citrate, MBP: D-maltose, E6B08_RS28125: l-ornithine, TodS: toluene, AdmX: indole-3-pyruvic acid, TtgV: benzonitrile. The asterisks indicate high-affinity binding, but data analysis with different models failed.

dCache domains (Fig. 1) form the predominant family of bacterial extracytosolic LBDs (16). They are found in about 15% of all chemoreceptors (38). The dCache LBD chemoreceptor PctA of P. aeruginosa PAO1 is a model to study this domain family (24, 39, 40). Microcalorimetric titrations of PctA-LBD with L-Ala showed an extremely broad pH range of high-affinity binding that stretched over 8.5 pH units, from pH 3.0 to 11.5 (Table 1, and Fig. 2 and 3A). Two other dCache LBDs were analyzed, namely, the purine-specific McpH (41) and polyamine-binding McpU (25). High-affinity ligand-binding was observed over 6.5 and 7.0 pH units for McpH-LBD and McpU-LBD, respectively, indicative of significant pH robustness (Fig. 3A).

**TABLE 1:** Summary of proteins analyzed in this study^*a*^

Protein name	Prot. type^*b*^	Strain	LBD family/pfam family	Ligand	pH range of binding^*c*^	pH optimum	*K*_D_ (μM) at pH optimum	Ref. for purification
LBDs of periplasmic location, α/β folds								
PctA-LBD	CR	P. aeruginosa PAO1	dCache_1/PF02743	L-alanine	3.0-11.5	5.5	0.59	(26)
McpH-LBD	CR	P. putida KT2440	dCache_1/PF02743	Adenine	3.5-10.0	5.0	1.68	(41)
McpU-LBD	CR	P. putida KT2440	dCache_1/PF02743	Putrescine	4.5-11.5	8.5	0.70	(94)
CtpM-LBD	CR	P. aeruginosa PAO1	sCache_2/PF17200	L-malate	4.5-9.0	6.5	38.7	(95)
McpV-LBD	CR	Sinorhizobium meliloti RU11*/*001	sCache_2/PF17200	Propionate	5.0-11.5	7.0	33.1	This study
LBDs of periplasmic location, four-helix bundle folds								
Tar-LBD	CR	E. coli K12	TarH/PF02203	L-aspartate	3.0-10.5	5.0	0.40	This study
PcaY_PP-LBD	CR	P. putida KT2440	TarH/PF02203	Quinate	3.5-11.5	5.0	1.22	(31)
McpS-LBD	CR	P. putida KT2440	HBM/PF16591	L-malate	3.0-10.0	5.5	4.0	(46)
McpQ-LBD	CR	P. putida KT2440	HBM/PF16591	Citrate	5.0–11.0	6.0	153	(47)
Periplasmic solute binding proteins								
MBP	SBP	E. coli K12	SBP_bac_1/PF01547	D-maltose	2.5-11.0	9.0	0.63	This study
E6B08_RS28125	SBP	P. putida 1290	SBP_bac_3/PF00497	L-ornithine	3.0-11.0	9.0	0.66	(50)
LBDs of cytosolic location								
TodS-Nter	SK	P. putida DOT-T1E	PAS_4/PF08448	Toluene	5.5-9.5	6.5	0.25	(96)
AdmX-LBD	TR	Serratia plymuthica A153	LysR_substrate/PF03466	Indole-3-pyruvic acid	7.5-8.0	8.0	12.7	(97)
TtgV	TR	P. putida DOT-T1E	IclR/PF01614	Benzonitrile	5.0-9.0	6.0	67.6	(98)

*^a^*The ITC curves and the derived dissociation constants are shown in Fig. S1 to S3 and Table S2.

*^b^*CR: chemoreceptor; SK: sensor histidine kinase; TR: transcriptional regulator; SBP: solute binding protein.

*^c^*Defined as binding with a *K*_D_ below 1 mM.

The dCache domain likely evolved through the fusion of monomodular sCache domains (16). We studied ligand recognition by 2 sCache family members, the malate-sensing CtpM-LBD and the propionate-sensing McpV-LBD. They bound ligands with high-affinity over a range of 4.5 and 6.5 pH units, respectively (Fig. 3A and Table 1), indicative of a slightly lower pH tolerance than the three dCache domains studied.

### Ligand-binding to periplasmic mono- and bimodular four-helixbundle ligand-binding domains.

**FIG 4:**
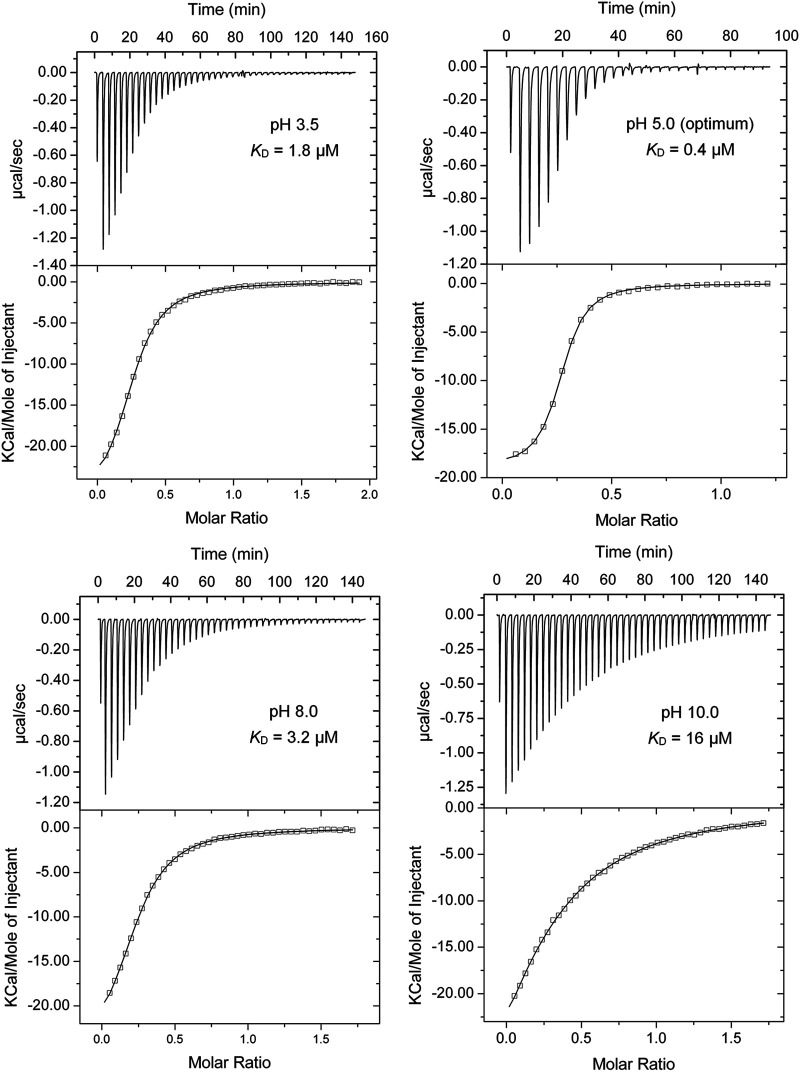
Microcalorimetric titrations of the E. coli Tar-LBD with L-Asp in buffers with different pH values. The protein concentration was between 32 and 37 μM, and the concentration of L-Asp was 300 μM. Upper panel: raw titration data. The pH and the corresponding *K*_D_ are shown. Lower panel: dilution-heat-corrected and concentration-normalized integrated raw data. Data were fitted with the “One-Binding Site Model” of the MicroCal (Northampton, MA, USA) version of ORIGIN. The derived dissociation constants are shown in Table S2.

**FIG 5:**
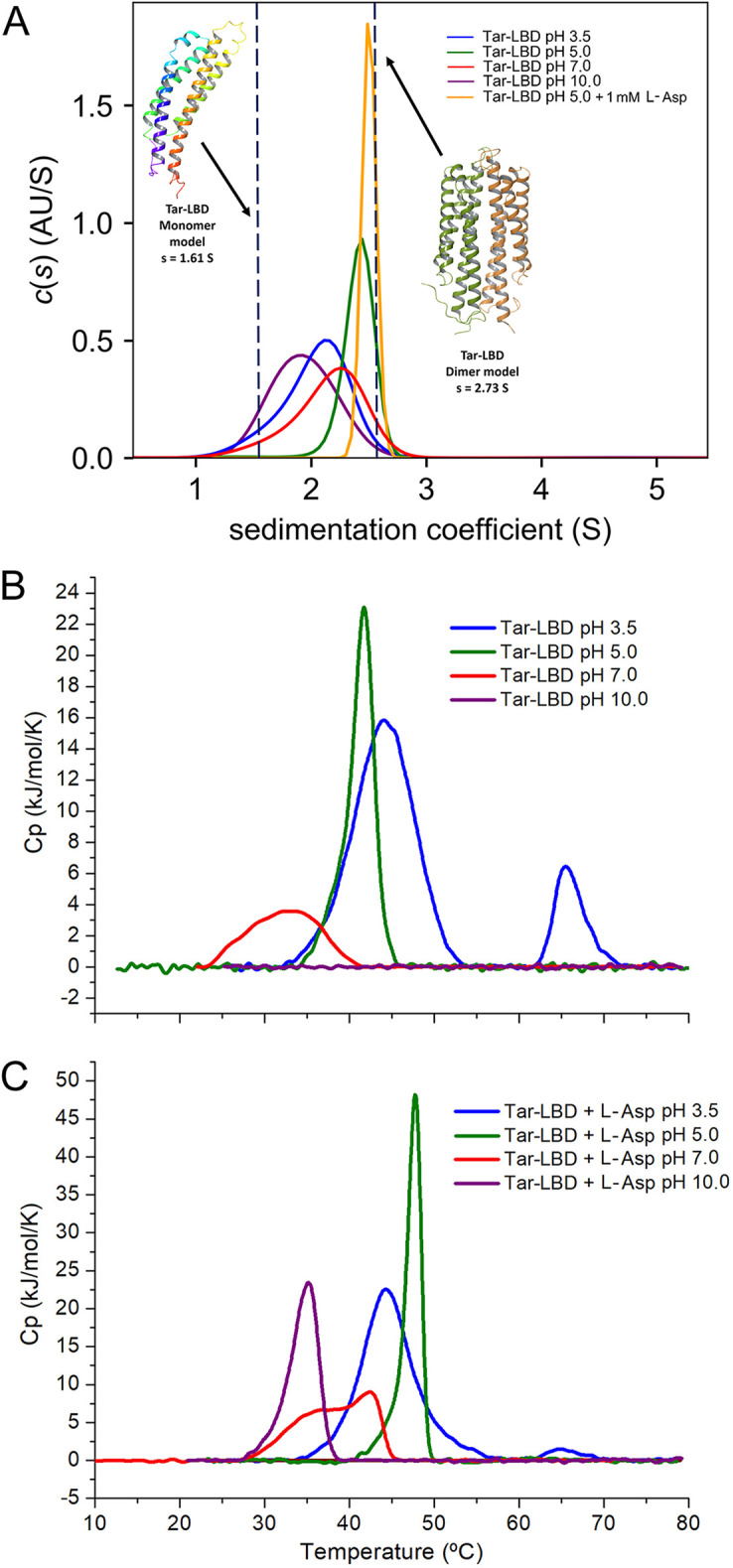
Analysis of the pH dependence of Tar-LBD by biophysical methods. (A) Sedimentation-velocity analytical ultracentrifugation studies at different pH values in the presence and absence of 1 mM L-Asp. The sedimentation-coefficient profiles of ligand-free Tar-LBD in buffers at pH 3.5, 5.0, 7.0, and 10.0 and of Tar-LBD in the presence of L-Asp at pH 5.0. Dashed lines indicate the theoretical sedimentation coefficient estimated from the hydrodynamic models of the Tar-LBD monomer and dimer. (B) and (C) Differential Scanning Calorimetry studies at different pH in the absence (B) and presence (C) of 1 mM L-Asp.

The aspartate chemoreceptor Tar of E. coli has been used in numerous studies to investigate chemotaxis and chemoreceptor function (42–44). Tar has a TarH-type mono-modular four-helix bundle LBD (Fig. 1). We cloned a DNA fragment encoding its LBD into an expression vector and purified the protein. As we observed with the α/β domains, Tar-LBD showed a remarkable pH robustness and bound ligands with high-affinity over 7.5 pH units, from pH 3.0 to 10.5 (Fig. 3B and Fig. 4). Studies at neutral pH have shown that the Tar-LBD must dimerize to detect its ligands, which bind at the dimer interface (28, 46). To assess the oligomeric state of the Tar-LBD at different pH values, we conducted sedimentation-velocity analytical ultracentrifugation (AUC) studies at pH 3.5, 5.0, 7.0 and 10.0 in the presence and absence of 1 mM L-Asp (Fig. 5A and Fig. S4). The assays clearly show that the pH of the medium affects the self-association equilibrium of Tar-LBD. To estimate the sedimentation coefficient expected for the monomeric and dimeric states of the Tar-LBD, a hydrodynamic model was generated from the 3D structure of the protein (pdb ID 4Z9H) (45). Using the HYDROPRO software, the theoretical sedimentation coefficients were s(20,w) = 2.12 S for the monomer and 3.54 S for the dimer (Fig. 5A). For all ligand-free protein samples, a single peak within this range presumably represents rapidly interchanging monomeric and dimeric forms. This single peak implies that rapid interconversion between monomers and dimers occurs much faster than the time scale of the sedimentation velocity experiment. At pH 5.0, the sedimentation coefficient was 3.18 S close to the expected value for the dimer. The sedimentation coefficient was 2.81 S at pH 7.0, 2.67 S at pH 3.5, and 2.53 S at pH 10.0 (Fig. 5A). Thus, the optimum pH for formation of Tar-LBD dimers is 5.0 that coincides with pH optimum of binding (*K*_D_ = 0.4 μM, Fig. 3B and Fig. 4), and the ligand affinities decrease in the same order as the sedimentation coefficients. Upon analyzing all the pH values, it was seen that the addition of 1 mM L-Asp shifted the equilibrium almost completely to the dimeric species (Fig. 5A and Fig. S4).

10.1128/mbio.01650-22.6 FIG S2 Microcalorimetric titrations of mono- and bimodular four-helix bundle fold ligand-binding domains with ligands at different pH. Upper panels: raw titration data. Lower panels: integrated, dilution heat-corrected and concentration-normalized raw data. The line corresponds to the best fit using the “One-Binding-Site Model” of the MicroCal version of ORIGIN. Download FIG S2, PDF file, 2.3 MB. Copyright © 2022 Monteagudo-Cascales et al. 2022 Monteagudo-Cascales et al. https://creativecommons.org/licenses/by/4.0/ This content is distributed under the terms of the Creative Commons Attribution 4.0 International license.

10.1128/mbio.01650-22.7 FIG S3 Microcalorimetric titrations of solute binding proteins and cytosolic ligand-binding domains with ligands at different pH. Upper panels: raw titration data. Lower panels: integrated, dilution heat-corrected and concentration-normalized raw data. The line corresponds to the best fit using the “One-Binding-Site Model” of the MicroCal version of ORIGIN. Download FIG S3, PDF file, 0.9 MB. Copyright © 2022 Monteagudo-Cascales et al. 2022 Monteagudo-Cascales et al. https://creativecommons.org/licenses/by/4.0/ This content is distributed under the terms of the Creative Commons Attribution 4.0 International license.

10.1128/mbio.01650-22.8 FIG S4 Sedimentation-velocity analytical ultracentrifugation studies of Tar-LBD at different pH values in the presence and absence of 1 mM L-Asp. The sedimentation-coefficient profiles of Tar-LBD in buffers at pH 3.5, 7.0 and 10.0 in the absence and presence of L-Asp. Dashed lines indicate the theoretical sedimentation coefficient estimated from the hydrodynamic models of the Tar-LBD monomer and dimer. Download FIG S4, TIF file, 0.6 MB. Copyright © 2022 Monteagudo-Cascales et al. 2022 Monteagudo-Cascales et al. https://creativecommons.org/licenses/by/4.0/ This content is distributed under the terms of the Creative Commons Attribution 4.0 International license.

Differential scanning calorimetry (DSC) provides information on the thermodynamics of protein unfolding. At 5.0, the optimal pH for binding and dimerization, Tar-LBD formed a sharp peak with or without 1 mM L-Asp (Fig. 5B and C, and Table S3), indicative of highly cooperative unfolding. At pH 10.0, at which the lowest amount of dimer and lowest affinity for L-Asp was observed, no cooperative unfolding was observed without L-Asp, and in the presence of L-Asp the protein was least stable. We thus conclude that at all pH values tested, at least some of the protein was present in the dimeric form and capable of binding ligand. There was a general correlation between the affinity (ITC), oligomeric state (AUC), and cooperativity of thermal unfolding (DSC) (Fig. 3B, 4, and 5), suggesting that pH-mediated modulation of the amount of protein dimer is a key event that determines ligand recognition. Analysis of another monomodular four-helix bundle domain, the TarH-type LBD of the quinate-sensing PcaY_PP chemoreceptor of Pseudomonas putida, showed a similarly broad pH profile that extended over 8 pH units (3.5-11.5) (Fig. 3B).

10.1128/mbio.01650-22.3 TABLE S3 Thermodynamic parameters for the thermal unfolding of Tar-LBD at different pH in the absence and presence of 1 mM L-Asp. Download Table S3, PDF file, 0.08 MB. Copyright © 2022 Monteagudo-Cascales et al. 2022 Monteagudo-Cascales et al. https://creativecommons.org/licenses/by/4.0/ This content is distributed under the terms of the Creative Commons Attribution 4.0 International license.

The HBM domain belongs to the superfamily of bimodular four-helix bundle LBDs (Fig. 1) (28). We have studied ligand recognition by 2 characterized HBM-containing P. putida chemoreceptors for organic acids, McpS (46) and McpQ (47). Like the TarH-type LBDs, McpS-LBD, and McpQ-LBD bound their ligands over pH ranges of 7 and 7.5 units, respectively (Table S2 and Fig. 3B).

### The pH robustness of ligand-binding to periplasmic solute-binding proteins.

Bacteria contain SBPs that float freely in the periplasm (Gram-negative bacteria) or that are lipoproteins tethered to outer face of the cell membrane (Gram-positive bacteria). Solute-binding proteins deliver substrates to their cognate transmembrane transporters, but some also activate signal transduction receptors by binding to their LBDs (37). The E. coli maltose-binding protein (MBP) is a primary model to study SBPs (48, 49). We have used E. coli MBP and E6B08_RS28125, an amino acid-sensing SBP of P. putida (50), as models to study the pH robustness of ligand recognition in solute-binding proteins. Like the periplasmic LBDs, MBP and E6B08_RS28125 show extraordinary pH robustness and recognized their ligands, D-maltose and l-ornithine, respectively, over ranges of 8.5 and 8.0 pH units, respectively (Fig. 3C and Fig. S5). Of particular note is the remarkable capacity of MBP to recognize maltose at the strongly acidic pH of 2.5 (*K*_D_ = 26 μM) (Fig. S5).

10.1128/mbio.01650-22.9 FIG S5 Microcalorimetric titrations of the E. coli maltose-binding protein with maltose in buffers with different pH. The protein concentration was between 34 and 40 μM, whereas the maltose concentration was between 0.5 and 2 mM. Upper panel: raw titration data. Shown is the pH and corresponding dissociation constant. Lower panel: dilution heat-corrected and concentration-normalized integrated raw data. Data were fitted with the “One-Binding-Site Model” of the MicroCal (Northampton, MA, USA) version of ORIGIN. Data obtained in pH 11.0 revealed high-affinity binding, but no satisfactory curve fit was obtained using different models for multiphasic interactions. Download FIG S5, TIF file, 1.8 MB. Copyright © 2022 Monteagudo-Cascales et al. 2022 Monteagudo-Cascales et al. https://creativecommons.org/licenses/by/4.0/ This content is distributed under the terms of the Creative Commons Attribution 4.0 International license.

### Analysis of cytosolic ligand-binding domains.

The cytosolic pH is generally maintained in the narrow range of 7.5–7.7 in neutralophilic bacteria (12). To assess the pH robustness of cytosolic sensor domains, we chose members of 3 very populated families for testing: PAS, IclR, and LysR_substrate (1, 22). The PAS domain of the cytosolic sensor kinase TodS recognized its ligand toluene over a relatively narrow range of 4 pH units, and a similar pH dependence was observed for the binding of benzonitrile to the IclR domain of the TtgV transcriptional regulator (Table 1). Furthermore, our analysis of AdmX-LBD, which belongs to the very populated LysR_substrate family of transcriptional regulators (51), revealed indole-3-pyruvic acid binding only at pH values of 7.5 and 8.0 (Fig. 3D and Table S2).

### The pH dependence of P. aeruginosa chemotaxis.

**FIG 6:**
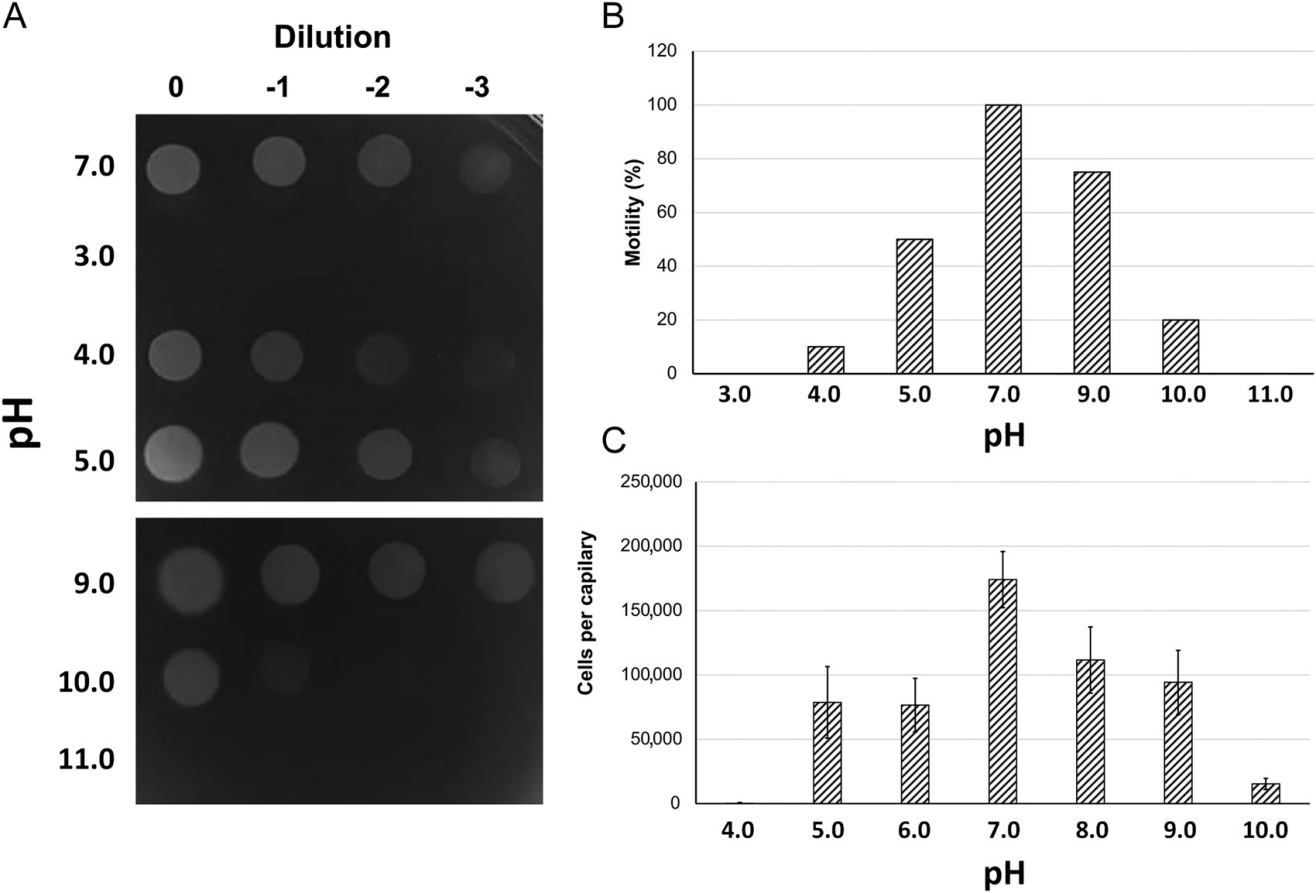
The effect of pH on cell viability, motility, and chemotaxis of P. aeruginosa PAO1. (A) Cell viability following a 1-h incubation in buffers at different pH, (B) Estimation of cell motility following a 1-h incubation in buffers of different pH. The percentage of motile cells is shown with respect to the total cell number. (C) Quantitative capillary chemotaxis assays toward 1 mM l-alanine (PctA ligand) using the chemotaxis buffers at different pH values. The number of bacteria that swam into buffer-containing capillaries was subtracted from the number of bacteria that migrated into chemoeffector containing capillaries. The number of bacteria that swam into buffer-containing capillaries were: pH 4.0: 354 ± 126; pH 5.0: 3,765 ± 1,040; pH 6.0: 6,609 ± 1,744; pH 7.0: 8,352 ± 2,574; pH 8.0: 4,337 ± 1,088; pH 9.0: 11,291 ± 2,415; pH 10.0: 1,873 ± 295. Data are the means and the standard deviations from 3 biological replicates conducted in triplicate.

We evaluated the effect of pH on P. aeruginosa chemotaxis to L-Ala, a ligand of the PctA chemoreceptor, using the quantitative capillary chemotaxis assay. Prior to performing these assays, we evaluated cell viability and motility at different pH values. We grew cells in minimal medium to an OD_660_ of 0.4, washed twice with chemotaxis buffer at the different pH values, resuspended in the corresponding buffer, and incubated for 1 h. Cells were then plated out to assess cell viability. As shown in Fig. 6A, viability after incubation at pH 5.0 to 9.0 was comparable. A significant reduction in viability was observed at pH 4.0 and 10.0, whereas cells were not viable at more extreme pH values. Cell motility after 1-h incubation showed a bell-shaped dependence on buffer pH. Whereas all cells were motile at pH 7.0, reduced motility was observed at pH 5.0 and 9.0 (Fig. 6B). At pH 4.0 and 10.0, motility was strongly decreased, which is expected given that cell survival was compromised at pH 4.0 and 10.0. We then determined the effect of different pH on the chemotactic responses to L-Ala. Optimal responses were observed at pH 7.0 and diminished gradually at lower and higher pH (Fig. 6C). Overall, the pH dependencies of motility and chemotaxis were similar, indicating that the reduction in motility is the reason for the reduction in chemotaxis.

### pH robustness of intracellular pathway response mediated by TarH and HBM receptors.

Measuring signaling output at different pH values by following chemotaxis is limited since pH motor activity is based on proton influx. Consequently, the changes in motility and chemotaxis observed above are likely to be related to differential activity of the motor. Thus, to test pH robustness of ligand sensing *in vivo* in a way that does not depend on cell motility, we performed FRET measurements of the pathway response in E. coli. This FRET assay monitors phosphorylation-dependent interaction between the response regulator CheY fused to yellow fluorescent protein (CheY-YFP) and its phosphatase CheZ fused to cyan fluorescent protein (CheZ-CFP), which depends linearly on the chemotaxis pathway activity (43, 52, 53). This FRET assay was previously used to characterize sensory responses of native E. coli chemoreceptors (54, 55), including their capacity to sense pH (5, 54), as well as of the chemoreceptor LBDs from other bacteria by fusing LBDs to the signaling domain of E. coli receptor Tar (18, 19, 56). Such direct FRET measurement of the chemotaxis pathway response is comparatively fast, which minimizes potential adverse effects of extreme pH on cell physiology, and it is independent of pH effects on motility.

**FIG 7:**
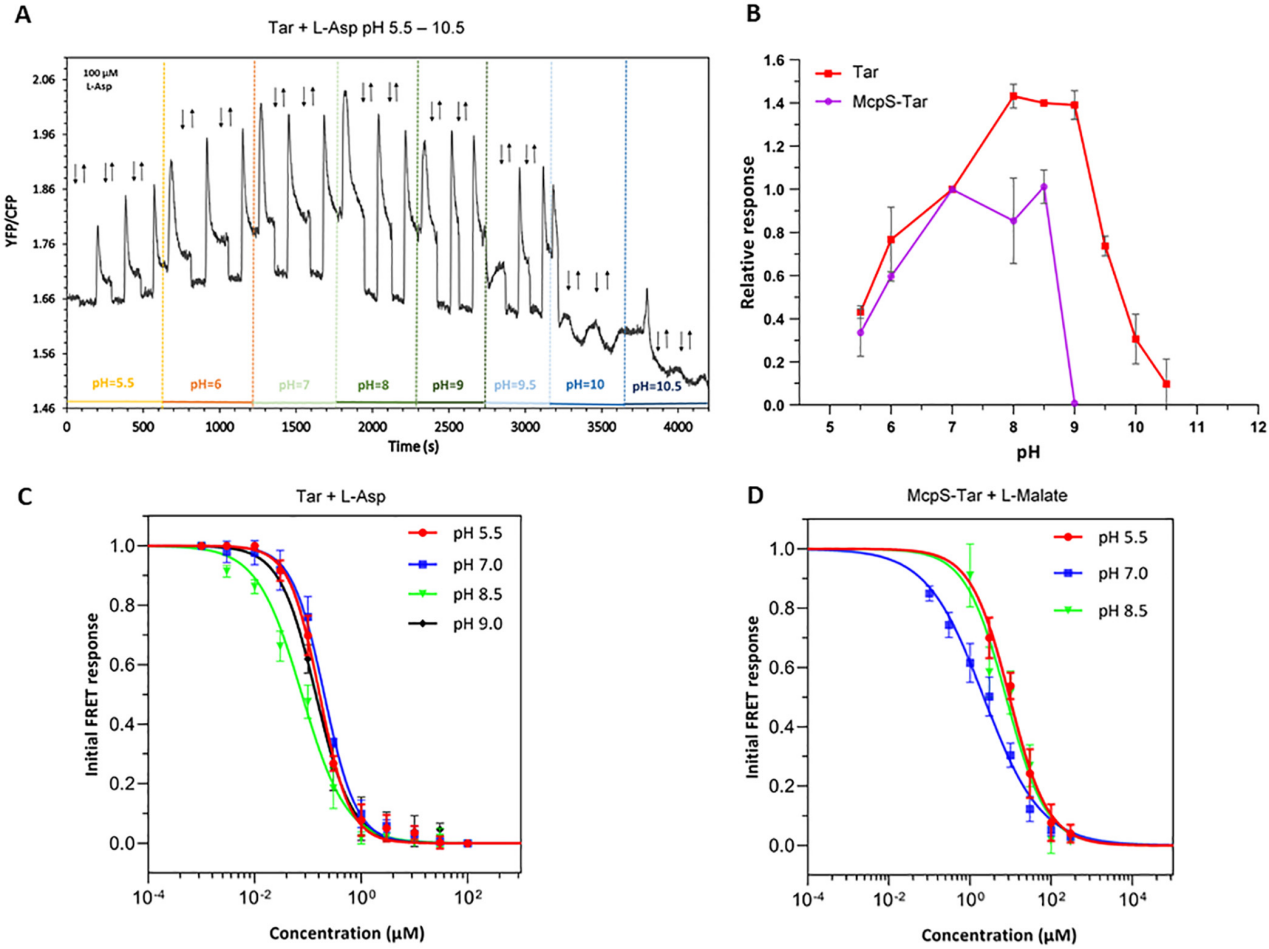
Chemotaxis pathway responses mediated by Tar and McpS-Tar at different pH. (A) FRET measurements of the responses of E. coli strain VS181 expressing CheY-YFP/CheZ-CFP FRET pair and Tar as sole chemoreceptor, to a stepwise addition and subsequent removal of 100 μM L-Asp (indicated by down and up arrows, respectively) at different values of ambient pH (indicated with different colors). The ratio of YFP to CFP fluorescence represents the FRET signal and thus the activity of the chemotaxis pathway. (B) Response amplitudes of the VS181 strain carrying Tar (red) or McpS-Tar (purple) to their specific ligands, 100 μM L-Asp or 100 μM L-malate, respectively, as a function of pH, normalized to the response at pH 7.0. Dose dependence of responses mediated by Tar (C) and by McpS-Tar (D) at different values of ambient pH. The amplitudes of the initial FRET response were calculated from changes in the ratio of YFP/CFP fluorescence after stimulation with the indicated ligand concentrations and normalized to the saturated response. Error bars indicate the standard errors of three independent experiments; wherever they are invisible, error bars are smaller than the symbol size. Data were fitted using Hill equation.

To investigate the pH dependence of responses mediated by the LBDs of E. coli Tar and P. putida McpS chemoreceptors, either Tar or a McpS-Tar chimeric receptor that was constructed by fusing McpS-LBD (including transmembrane helices) to the signaling domain (including HAMP domain) of Tar, were expressed as the sole chemoreceptor in a receptorless E. coli background strain VS181 also carrying the FRET pair encoded by the pVS88 plasmid. Responses to stepwise additions of respective ligands were measured by monitoring the ratio of YFP to CFP fluorescence that is indicative of FRET. We observed that over a broad range of background pH values of the medium, from 5.5 to 10.5, Tar-expressing cells exhibited an attractant response to a stepwise addition of a saturating concentration (100 μM) of L-Asp, seen as rapid reduction in the ratio of YFP/CFP fluorescence due to attractant-mediated pathway inhibition (Fig. 7A and B). Subsequent removal of attractant after partial adaptation of cells elicited a repellent response, which is seen as an increased YFP/CFP ratio (52). The amplitude of the response to L-Asp was maximal at neutral and slightly alkaline pH, but it dropped at both low and high pH, possibly reflecting adverse effects of extreme pH on signaling or on the fluorescence readout. Of note, repellent responses were also observed upon an increase in the background pH, consistent with Tar-mediated pH sensing (5). Similarly, FRET responses were observed over 3 pH units, from 5.5 to 8.5, for the McpS-Tar hybrid stimulated with L-malate (Fig. S6A and Fig. 7B). This range of pH tolerance for McpS-Tar was narrower compared to Tar, consistent with the ITC data (Fig. 3B).

10.1128/mbio.01650-22.10 FIG S6 FRET measurements of responses mediated by McpS-Tar and Tar at different pH. (A) Responses of E. coli strain VS181 expressing McpS-Tar as a sole receptor to a stepwise addition and subsequent removal of 100 μM L-malate (indicated by down and up arrows, respectively) at different ambient pH (indicated with different colors). Dose responses of buffer-adapted E. coli harboring Tar (B) or McpS-Tar (C) toward their specific ligands (L-Asp for Tar and L-malate for McpS-Tar) at different pH. Download FIG S6, TIF file, 0.2 MB. Copyright © 2022 Monteagudo-Cascales et al. 2022 Monteagudo-Cascales et al. https://creativecommons.org/licenses/by/4.0/ This content is distributed under the terms of the Creative Commons Attribution 4.0 International license.

To determine the effect of pH on the signaling output *in vivo*, we measured the dose responses for both receptors at different values of background pH. Ligand sensitivity of both receptors showed little pH dependence within the tested range, with only slightly higher apparent sensitivity (i.e., lower values of EC_50_, the ligand concentration that elicits a half-maximal response) at intermediate pH (Fig. 7C and D, Fig. S6B and C, and Table 2). This was again in a general agreement with effects of pH on ligand-binding to Tar-LBD and McpS-LBD (Fig. 3B), including similar values of *K*_D_ and EC_50_ at the optimal pH (Tables 1 and 2, and Fig. 7C and D), although for both LBDs the pH optimum of the *in vivo* response is shifted toward higher pH values compared to the biochemical data. One possible explanation for this difference is that the periplasmic pH might be lower than the pH of the medium.

**TABLE 2:** The EC_50_ (half-maximal effective concentration) values for the FRET titration of E. coli cells harboring Tar or McpS-Tar as the sole chemoreceptors with different concentrations of L-Asp and L-malate, respectively

	EC_50_ (μM)	
pH	Tar	McpS-Tar
5.5	0.166 ± 0.02	9.378 ± 1.9
7.0	0.202 ± 0.02	2.178 ± 0.41
8.5	0.077 ± 0.01	7.659 ± 1.9
9.5	0.141 ± 0.02	

To summarize, our results strongly suggest that not only ligand-binding but also the signaling responses mediated by chemoreceptors are robust within the broad range of pH. Notably, Tar maintains its response over the entire range of pH values that support growth of E. coli, thus further confirming physiological relevance of the observed pH robustness of ligand-binding.

## DISCUSSION

The capacity of bacteria to sense environmental cues and adapt their metabolism and lifestyle accordingly is critical for survival (2). To this end, bacteria have evolved a large arsenal of different families of transmembrane receptors that bind signals at extracytoplasmic LBDs (2). LBDs are ligand-binding modules that contain all the molecular determinants for ligand recognition. The same type of LBD is shared by all the members of a given receptor family.

Large changes in environmental pH are among the environmental challenges to which bacteria are exposed. The outer membrane of Gram-negative bacteria is highly permeable for pH-active compounds, and the periplasmic pH is very close to that of the external medium (10). The central question addressed in this work is to what degree alterations in the pH modulate the capacity of LBDs to recognize their cognate ligands. Much experimental data exists for the microcalorimetric analyses of different ligand – LBD interactions at neutral pH (1, 57). However, there has been no systematic evaluation of the pH robustness of these interactions.

This study analyzed the pH dependence of ligand recognition for 9 periplasmic LBDs, 2 periplasmic SBPs, and 3 cytosolic LBDs. The average range of pH values at which at least moderate binding was seen with the periplasmic domains/proteins was 7.1 ± 1.2 pH units. This was significantly broader than the corresponding range for the cytosolic domains (3.0 ± 2.1 pH units). Thus, periplasmic LBDs show significantly greater pH robustness than their cytosolic equivalents (Table 1). A clear tendency has been observed for the pH optima of the periplasmic LBDs. Of the 9 periplasmic domains analyzed, 7 had a pH optimum in the slightly acidic pH range and 5 proteins recognized their ligands with highest affinity at pH 5.0 or 5.5 (Fig. 3 and Table S2).

Numerous studies have assessed the pH dependence of cytosolic enzymes. Typically, enzymes show activity over a range of 3 to 5 pH units, as exemplified by a glutathione *S*-transferase (58), D-amino acid dehydrogenase (59) and pyridoxal kinase (each pH 5.0–9.0) (60), cystathione synthase (pH 6.0–9.0) (61), amylase (pH 6.5–10.0) (62), galactosidase (pH 5.5–8.5) (63), or a lactase (pH 3.0–5.0) (64). The width of activities is thus similar to the pH dependence observed for cytosolic LBDs and significantly inferior to the pH robustness of extracytoplasmic LBDs, which this study reveals frequently retain significant binding activity over 7 or 8 pH units.

Several strategies have been identified that permit bacteria to cope with extreme pH in the periplasm, such as chaperones for protein refolding or the secretion of CO_2_ (converted rapidly into bicarbonate) or NH_3_ into the periplasm to serve as buffering agents (11, 12). Our data suggest that the evolution of intrinsic pH robustness of extracellular sensor domains is another mechanism by which bacteria cope with extremes in environmental pH.

The demonstration that the pH robustness of sensing at extracytoplasmic sensor domains is superior to that of their cytosolic equivalents leads to the question of the corresponding structural determinants. There are several possibilities to advance the knowledge in this area. There are thousands of 3D structures of extra- and intracytosolic sensor domains available at the protein data bank (65). Large-scale computational approaches can be used to determine different parameters in extra- and intracytosolic sensor domains like (i) the overall amino acid composition and that of the ligand-binding pocket, particularly the content in titratable amino acids, (ii) the number of intramolecular interactions such as hydrogen bonds, salt bridges and pi-stacking interactions per residue as potential indicators of structural robustness. Alternatively, selected structures of extra- and intracytosolic sensor domains can analyzed by sophisticated computational approaches like the Molecular Transfer Model that includes the findings of Tanford et al. who have shown that it is possible to predict the protein stability at different pH, taking into account the pKa values of titratable groups (66, 67). There are a number of studies that indicate the importance of the pKa values of titratable amino acid residues for protein stability at different pH (68–70). This notion is illustrated by the study of Mishra et al. showing that the perturbed pKa of a single ionizable residue functions as a pH-dependent protein stability switch (71).

Importantly, the pH robustness of sensor domains in which ligands interact with a single protein chain (i.e., mono- and bimodular α/β folds) was comparable to domains in which ligands bind at the dimer interface and in which the sensor domain must be dimeric for binding (i.e., mono- and bimodular four-helixbundle folds). The pH robustness of the latter domain superfamily indicates that this domain family has evolved to be at least partially dimeric over a wide pH range to maintain that capacity to bind ligands. This notion is supported by analytical ultracentrifugation studies of Tar-LBD showing that samples at all pH values tested are mixtures of monomeric and dimeric species (Fig. 5A).

The most abundant extracytosolic LBDs are the dCache and four-helixbundle LBD (16, 22, 38). We have analyzed in detail the sensor domains of 2 central model proteins of both families, namely, PctA (24, 39, 40) and Tar (42–44). PctA-LBD was found to possess remarkably constant binding parameters, with *K*_D_ values between pH 3.5 to 10.5 remaining between 0.86 to 1.50 μM. Such binding behavior is ideally suited for the construction of robust biosensors. Tar-LBD was the periplasmic LBD with highest affinity, with a *K*_D_ of 400 nM at pH 5.0.

Most proteins analyzed in our study are chemoreceptor sensor domains, and we have therefore investigated P. aeruginosa chemotaxis to L-Ala (recognized by PctA) over a pH range. The dependence of cell motility and chemotaxis on pH showed a similar behavior, suggesting that the pH-mediated reduction in chemotaxis is related to the reduction in motility. Significant chemotaxis was only observed over the pH 5.0 to 9.0. This range is thus narrower than the range of strong L-Ala binding by PctA-LBD, which stretched from pH 3.0 to 11.5 (Fig. 3).

This situation raises the question of why PctA-LBD binds L-Ala over such a wide range of pH? The chemotaxis assay involved the exposure of cells to a given pH for about 1 h (including washing steps and the actual assay). It can be hypothesized that bacteria maintain their capacity to sense during a short-term exposure to extreme pH, such as for example when swimming through a heterogeneous environment characterized by drastic but brief local changes in pH. The evolution of the observed pH robustness in ligand recognition may thus have been driven by the capacity to maintain the sensing capacity during short-term exposure to extreme pH.

The notion that the effect of extreme pH on motility and motor function may be the cause for the restricted capacity to perform chemotaxis over a relatively narrow pH range is supported by FRET measurements, in which the output is unrelated to motility and motor function. Microcalorimetric studies have shown that Tar-LBD is able to bind L-Asp up to pH 10.5. In close analogy, FRET responses were also observed up to pH 10.5. The agreement of the pH dependence of signaling input and output demonstrates that signal sensing, even at a strongly alkaline pH like 10.0 to 10.5, triggers a response. In addition, the Tar EC_50_ values showed little variation over the pH range analyzed, indicating that the observed pH robustness of sensing corresponds to a robust output.

Our study has important implications for the development of biosensors, which are devices that measure biological or chemical reactions by generating signals proportional to the concentration of an analyte (72). Biosensor development is a dynamic and rapidly expanding field of research (73). Currently, more than 8000 publications are published annually that contain the term biosensor in the title or abstract. This number has almost doubled in the last 10 years. The biosensor market has been evaluated as being 19.6 billion USD in 2019 and is estimated to grow at an enormous 7.9% annually to reach 36 billion USD by 2027 (74).

The COVID-19 pandemic has impressively demonstrated the usefulness and importance of biosensors (75). Optical biosensors, like those based on surface plasmon resonance, are the most common biosensor type (76). Typically, these sensors contain an immobilized biomolecule, frequently a protein that captures the analyte. The enormous diversity of ligand specificities found among extracytoplasmic LBDs (1) makes them an abundant reservoir for biosensor construction. There is an almost unlimited number of different ligands that bind to extracytosolic LBDs, which can thus be used for the development of novel biosensors. These compounds include clinically important compounds such as the neurotransmitters epinephrine (77), γ-aminobutyric acid (GABA) (56), histamine (78) or acetylcholine (79), antimicrobial peptides (80), amino acids (40), organic acids (46), sugars (81), fatty acids (82), quorum-sensing molecules (39), plant hormones (83), aromatic hydrocarbons (84), purines (41), polyamines (25), quaternary amines (85), nitrate (86), inorganic phosphate (87), and specific metal cations (88) or oxanions (30). The demonstration that extracytosolic LBDs maintain their capacity to sense their ligands over a wide pH range will permit the construction of biosensors that are robust and able to withstand changes in the analyte medium.

## MATERIALS AND METHODS

### Strains and plasmids.

The strains, plasmids, and oligonucleotides used in this study are provided in Table S4.

10.1128/mbio.01650-22.4 TABLE S4 Strains, plasmids, and oligonucleotides used in this study. Download Table S4, PDF file, 0.1 MB. Copyright © 2022 Monteagudo-Cascales et al. 2022 Monteagudo-Cascales et al. https://creativecommons.org/licenses/by/4.0/ This content is distributed under the terms of the Creative Commons Attribution 4.0 International license.

### Construction of expression plasmids for Tar-LBD, McpV-LBD, and MBP.

A DNA fragment encoding the Tar-LBD (amino acids 31 to 188) was amplified from genomic DNA of E. coli K12 and cloned into the NdeI and BamHI sites of pET28b(+) to generate pET28-Tar-LBD. A DNA fragment encoding amino acids 27 to 396 (full-length sequence lacking signal peptide) of E. coli K12 MBP was amplified from genomic DNA and cloned into the NdeI and XhoI sites of pET28b(+), resulting in pET28-MBP. A DNA fragment encoding the McpV-LBD (amino acids 33 to 189) was amplified from plasmid pBS377 and cloned into the NdeI and HindIII sites of pET28b(+) to generate pET28-McpV-LBD. All plasmids were verified by sequencing.

### Overexpression and purification of proteins.

With the exception of Tar-LBD, McpV-LBD, and MBP, proteins were overexpressed and purified according to the references indicated in Table 1. For the generation of Tar-LBD, McpV-LBD, and MBP, Escherichia coli BL21(DE3) containing plasmids pET28-Tar-LBD, pET28-McpV-LBD, or pET28-MBP, respectively, were grown in 2 L Erlenmeyer flasks containing 400 to 500 mL of LB medium supplemented with 50 μg mL^−1^ kanamycin at 30°C. At an OD_600_ of 0.5, the growth temperature was lowered to 16°C (Tar-LBD) or 18°C (McpV-LBD and MBP) and growth continued for another 30 min (Tar-LBD) or 15 min (McpV-LBD and MBP) prior to the induction of protein expression by the addition of β-D-1-thiogalactopyranoside (IPTG) to a final concentration of 0.1 mM (Tar-LBD and McpV-LBD) or 0.5 mM (MBP). Growth was continued overnight prior to cell harvest by centrifugation at 10 000 × *g* for 30 min at 4°C. All subsequent manipulations were made at 4°C. Tar-LBD, McpV-LBD and MBP pellets were resuspended in buffer A (20 mM Tris/HCl, 0.1 mM EDTA, 300 mM NaCl, 10 mM imidazole, 5% (vol/vol) glycerol, pH 8.0), B (30 mM Tris/HCl, 500 mM NaCl, 10 mM imidazole, 5% (vol/vol) glycerol, pH 8.0) or C (30 mM Tris/HCl, 300 mM NaCl, 10 mM imidazole, 5% (vol/vol) glycerol, pH 8.0), respectively, and broken by French press treatment at 1000 lb/in^2^. After centrifugation at 20 000 × *g* for 1 h, the supernatant was loaded onto 5 mL HisTrap columns (Amersham Bioscience), equilibrated with buffers A, B, or C. The columns were washed with 5 column volumes of buffers A, B, or C containing 35 mM imidazole. Protein was eluted by applying a 35–500 mM imidazole gradient in buffers A, B, or C. Protein-containing fractions were pooled and dialyzed for immediate analysis.

### Isothermal titration calorimetry.

Freshly purified proteins were dialyzed into the buffers specified in Table S1 and introduced into the sample cell of an VP-microcalorimeter (MicroCal). Proteins at 15 to 100 μM were titrated with 0.25 to 10 mM ligand solutions that were made up in dialysis buffer. For pH-active ligands, the pH of the ligand solutions was adjusted to that of the dialysis buffer by the addition of concentrated HCl or NaOH. Titrations of MBP and McpV-LBD were conducted at 15°C to favor endothermic contributions to binding heats, whereas the remaining titrations were carried out at 25°C. Raw titration data were integrated, corrected for ligand-dilution heats and normalized with the ligand concentrations. Resulting data were fitted with the “One Binding site model” of the MicroCal version of ORIGIN. Provided are the dissociation constants derived from one experiment and the error of curve-fitting.

### Analytical ultracentrifugation.

Experiments were conducted on a ProteomeLab XL-I instrument (Beckman-Coulter) equipped with interference and absorbance optics. Sedimentation was carried out at 42,000 rpm and 10°C in an eight-hole Ti-50 Beckman Coulter rotor and monitored with absorbance optics at 280 nm in continuous mode. Samples were dialyzed into 4 different buffers, namely, 10 mM citric acid/sodium citrate, pH 3.5; 10 mM citric acid/sodium citrate, pH 5.0; 3 mM Tris, 3 mM PIPES, 3 mM MES, pH 7.0, and 10 mM Na_2_CO_3_/NaHCO_3,_ pH 10.0. For the analysis of the protein in the absence and presence of its ligand, dialyzed samples were diluted with either dialysis buffer or a stock solution of L-Asp in dialysis buffer to a final protein concentration of 35 μM and a ligand concentration of 1 mM. Density and viscosity of the buffer were calculated with SEDNTERP (89). Data preparation of native samples was performed with Sedfit v. 15.01b (90), using the non-interacting discrete species continuous Svedberg distribution model (c[S]). After fitting, data were exported to GUSSI v. 1.3.2 (91) for the preparation of Figures. To assign the sedimentation coefficient observed to the corresponding oligomeric species, theoretical *s* values were calculated via HYDROPRO (92) hydrodynamic modeling software, applied to the 3D structure of the Tar-LBD monomer and dimer (pdb ID 4H9Z), with a 2.9 Å radius for the primary elements.

### Differential scanning calorimetry.

Experiments were conducted on a MicroCal DSC-PEAQ system (Malvern Panalytical) at a scan rate of 90°C/h over a temperature range of 10°C to 85°C. Calorimetric cells (operating volume 0.134 mL) were kept under pressure (60 lb/in^2^) to prevent sample degassing. Buffer-buffer baselines obtained after each assay were subtracted from the signal obtained from each of the samples. Protein samples were prepared as detailed in the “Analytical ultracentrifugation” section. The calorimetric enthalpies were estimated by integration of the transition peaks after subtracting the buffer-buffer baselines and fitting the curves to a “Non-two-state” model using MicroCal DSC-PEAQ software. For graphical clarity of the plots, baselines were manually curated and raw data were smoothed with the Savitzky-Golay algorithm.

### Cell viability and motility.

Overnight cultures of P. aeruginosa PAO1 grown in M9 minimal medium (93) supplemented with 6 mg/mL Fe-citrate and 15 mM glucose, were used to inoculate fresh medium to an OD_660_ of 0.1. The cells were grown at 37°C to OD_660_ of 0.4, collected by centrifugation (1,667 × *g* for 5 min at room temperature), washed gently twice with chemotaxis buffer (50 mM KH_2_PO_4_/K_2_HPO_4_, 20 mM EDTA, 0.05% [vol/vol] glycerol) at different pH and resuspended in fresh chemotaxis buffer. After 1 h of incubation at room temperature without shaking, samples were inspected with a light microscope and the percentage of motile bacteria with respect to the total cell number determined. Serial dilutions were then plated out, incubated overnight at 30°C, and plates inspected visually.

### Capillary chemotaxis assays.

Chemotaxis assays were performed as previously described (79) with some modifications. When cultures reached an OD_660_ of 0.4, the cells were gently washed twice with chemotaxis buffer at different pH and then resuspended in fresh buffer. The chemoeffector solutions were prepared in the corresponding chemotaxis buffer. Data are the means and standard deviations of at least 3 biological replicates conducted in triplicate.

### FRET measurements.

FRET measurements were performed as described previously (52–54). Cultures of receptorless E. coli strain VS181 expressing Tar or McpS-Tar from respective plasmids induced by sodium salicylate and the CheY-YFP/CheZ-CFP FRET pair from plasmid pVS88 induced by IPTG, were inoculated with 200 μL of the overnight culture into 10 mL tryptone broth (TB; 1% tryptone and 0.5% NaCl) supplemented with ampicillin, chloramphenicol, 50 μM IPTG, and 2 μM sodium salicylate and grown in a rotary shaker at 34°C and 275 rpm. Cells were harvested at OD_600_ of 0.5 by centrifugation, washed with tethering buffer (10 mM KH_2_PO_4_/K_2_HPO_4_, 0.1 mM EDTA, 1 μM methionine, 10 mM sodium lactate, pH 7.0), resuspended in 10 mL tethering buffer and kept at 4°C. For measurements at different pH, the pH values of tethering buffer were adjusted by the addition of diluted HCl or NaOH, and working solutions of compounds were prepared using tethering buffer with corresponding pH. For microscopy, cells were attached to poly-lysine-coated coverslips for 10 min and mounted into a flow chamber that was maintained under constant flow of 0.3 mL/min of tethering buffer using a syringe pump (Harvard Apparatus) that was also used to add or remove compounds of interest. FRET measurements were performed on an upright fluorescence microscope (Zeiss AxioImager.Z1) equipped with photon counters (Hamamatsu). CFP fluorescence was excited and CFP and YFP fluorescence signals were recorded and analyzed as described previously (52, 54).
