# Supplementary material for: The pH Robustness of Bacterial Sensing
Source: mBio. 2022 Sep 26;13(5):e01650-22. doi: 10.1128/mbio.01650-22 (PMC9600550; doi:10.1128/mbio.01650-22)
Supplement: TABLE S1 [file mbio.01650-22-s0001.pdf]

Table S1) Buffers used for microcalorimetric studies. Composition of the dialysis buffers used for each of the proteins analysed. Dialyzed proteins were transferred into the microcalorimeter and ligand solutions were made up in dialysis buffer.

pH	Buffer composition
**Periplasmic LBDs, α/β folds**	
**CtpM-LBD**	
4.0	10 mM citric acid/sodium citrate, 150 mM NaCl, 10 % (v/v) (v/v) glycerol
4.5	10 mM citric acid/sodium citrate, 150 mM NaCl, 10 % (v/v) glycerol
5.0	10 mM citric acid/sodium citrate, 150 mM NaCl, 10 % (v/v) glycerol
5.5	10 mM citric acid/sodium citrate, 150 mM NaCl, 10 % (v/v) glycerol
6.0	3 mM Tris, 3 mM PIPES, 3 mM MES, 150 mM NaCl, 10 % (v/v) glycerol
6.5	3 mM Tris, 3 mM PIPES, 3 mM MES, 150 mM NaCl, 10 % (v/v) glycerol
7.0	3 mM Tris, 3 mM PIPES, 3 mM MES, 150 mM NaCl, 10 % (v/v) glycerol
7.5	3 mM Tris, 3 mM PIPES, 3 mM MES, 150 mM NaCl, 10 % (v/v) glycerol
8.0	3 mM Tris, 3 mM PIPES, 3 mM MES, 150 mM NaCl, 10 % (v/v) glycerol
8.5	3 mM Tris, 3 mM PIPES, 3 mM MES, 150 mM NaCl, 10 % (v/v) glycerol
9.0	3 mM Tris, 3 mM PIPES, 3 mM MES, 150 mM NaCl, 10 % (v/v) glycerol
9.5	10 mM Na_2CO_3/$NaHCO_3$, 150 mM NaCl, 10 % (v/v) glycerol
**McpV-LBD**	
4.0	10 mM citric acid/sodium citrate, 150 mM NaCl, 10 % (v/v) glycerol
4.5	10 mM citric acid/sodium citrate, 150 mM NaCl, 10 % (v/v) glycerol
5.0	10 mM citric acid/sodium citrate, 150 mM NaCl, 10 % (v/v) glycerol
6.0	3 mM Tris, 3 mM PIPES, 3 mM MES, 150 mM NaCl, 10 % (v/v) glycerol
7.0	3 mM Tris, 3 mM PIPES, 3 mM MES, 150 mM NaCl, 10 % (v/v) glycerol
8.0	3 mM Tris, 3 mM PIPES, 3 mM MES, 150 mM NaCl, 10 % (v/v) glycerol
9.0	3 mM Tris, 3 mM PIPES, 3 mM MES, 150 mM NaCl, 10 % (v/v) glycerol
10.0	10 mM Na_2CO_3/$NaHCO_3$, 150 mM NaCl, 10 % (v/v) glycerol
11.0	10 mM Na_2CO_3/$NaHCO_3$, 150 mM NaCl, 10 % (v/v) glycerol
11.5	10 mM Na_2PO_4/NaOH,150 mM NaCl, 10 % (v/v) glycerol
12.0	10 mM KCl/NaOH, 150 mM NaCl, 10 % (v/v) glycerol
**McpH-LBD**	
3.0	10 mM citric acid/sodium citrate
3.5	10 mM citric acid/sodium citrate
4.0	10 mM citric acid/sodium citrate
5.0	10 mM citric acid/sodium citrate
6.0	3 mM Tris, 3 mM PIPES, 3 mM MES
7.0	3 mM Tris, 3 mM PIPES, 3 mM MES
8.0	3 mM Tris, 3 mM PIPES, 3 mM MES
9.0	3 mM Tris, 3 mM PIPES, 3 mM MES
10.0	10 mM Na_2CO_3/$NaHCO_3$
10.5	10 mM Na_2CO_3/$NaHCO_3$
**PctA-LBD**	
2.5	20 mM acetic acid/HCl
3.0	10 mM citric acid/sodium citrate, 150 mM NaCl, 10 % (v/v) glycerol
3.5	10 mM citric acid/sodium citrate, 150 mM NaCl, 10 % (v/v) glycerol
4.0	10 mM citric acid/sodium citrate, 150 mM NaCl, 10 % (v/v) glycerol
4.5	10 mM citric acid/sodium citrate, 150 mM NaCl, 10 % (v/v) glycerol
5.0	10 mM citric acid/sodium citrate, 150 mM NaCl, 10 % (v/v) glycerol
5.5	10 mM citric acid/sodium citrate, 150 mM NaCl, 10 % (v/v) glycerol
6.0	3 mM Tris, 3 mM PIPES, 3 mM MES, 150 mM NaCl, 10 % (v/v) glycerol
6.5	3 mM Tris, 3 mM PIPES, 3 mM MES, 150 mM NaCl, 10 % (v/v) glycerol
7.0	3 mM Tris, 3 mM PIPES, 3 mM MES, 150 mM NaCl, 10 % (v/v) glycerol

7.5	3 mM Tris, 3 mM PIPES, 3 mM MES, 150 mM NaCl, 10 % (v/v) glycerol
8.0	3 mM Tris, 3 mM PIPES, 3 mM MES, 150 mM NaCl, 10 % (v/v) glycerol
8.5	3 mM Tris, 3 mM PIPES, 3 mM MES, 150 mM NaCl, 10 % (v/v) glycerol
9.0	3 mM Tris, 3 mM PIPES, 3 mM MES, 150 mM NaCl, 10 % (v/v) glycerol
9.5	10 mM $Na_2CO_3/NaHCO_3$, 150 mM NaCl, 10 % (v/v) glycerol
10.0	10 mM $Na_2CO_3/NaHCO_3$, 150 mM NaCl, 10 % (v/v) glycerol
10.5	10 mM $Na_2CO_3/NaHCO_3$, 150 mM NaCl, 10 % (v/v) glycerol
11.0	10 mM Na_2PO_4/NaOH,150 mM NaCl, 10 % (v/v) glycerol
11.5	10 mM Na_2PO_4/NaOH,150 mM NaCl, 10 % (v/v) glycerol
12.0	10 mM Na_2PO_4/NaOH,150 mM NaCl, 10 % (v/v) glycerol
**McpU-LBD**	
4.0	10 mM citric acid/sodium citrate, 150 mM NaCl, 10 % (v/v) glycerol
4.5	10 mM citric acid/sodium citrate, 150 mM NaCl, 10 % (v/v) glycerol
5.0	10 mM citric acid/sodium citrate, 150 mM NaCl, 10 % (v/v) glycerol
5.5	10 mM citric acid/sodium citrate, 150 mM NaCl, 10 % (v/v) glycerol
6.0	3 mM Tris, 3 mM PIPES, 3 mM MES,150 mM NaCl, 10 % (v/v) glycerol
6.5	3 mM Tris, 3 mM PIPES, 3 mM MES,150 mM NaCl, 10 % (v/v) glycerol
7.0	3 mM Tris, 3 mM PIPES, 3 mM MES,150 mM NaCl, 10 % (v/v) glycerol
7.5	3 mM Tris, 3 mM PIPES, 3 mM MES,150 mM NaCl, 10 % (v/v) glycerol
8.0	3 mM Tris, 3 mM PIPES, 3 mM MES,150 mM NaCl, 10 % (v/v) glycerol
8.5	3 mM Tris, 3 mM PIPES, 3 mM MES,150 mM NaCl, 10 % (v/v) glycerol
9.5	3 mM Tris, 3 mM PIPES, 3 mM MES,150 mM NaCl, 10 % (v/v) glycerol
10.0	10 mM $Na_2CO_3/NaHCO_3$, 150 mM NaCl, 10 % (v/v) glycerol
10.5	10 mM $Na_2CO_3/NaHCO_3$, 150 mM NaCl, 10 % (v/v) glycerol
11.0	10 mM $Na_2CO_3/NaHCO_3$, 150 mM NaCl, 10 % (v/v) glycerol
11.5	10 mM Na_2PO_4/NaOH,150 mM NaCl, 10 % (v/v) glycerol
12.0	10 mM Na_2PO_4/NaOH,150 mM NaCl, 10 % (v/v) glycerol
**Periplasmic LBDs, four-helix bundle folds**	
**Tar-LBD**	
2.5	20 mM acetic acid/HCl
3.0	10 mM citric acid/sodium citrate
3.5	10 mM citric acid/sodium citrate
4.0	10 mM citric acid/sodium citrate
4.5	10 mM citric acid/sodium citrate
5.0	10 mM citric acid/sodium citrate
5.5	10 mM citric acid/sodium citrate
6.0	3 mM Tris, 3 mM PIPES, 3 mM MES
6.5	3 mM Tris, 3 mM PIPES, 3 mM MES
7.0	3 mM Tris, 3 mM PIPES, 3 mM MES
7.5	3 mM Tris, 3 mM PIPES, 3 mM MES
8.0	3 mM Tris, 3 mM PIPES, 3 mM MES
8.5	3 mM Tris, 3 mM PIPES, 3 mM MES
9.0	3 mM Tris, 3 mM PIPES, 3 mM MES
9.5	10 mM $Na_2CO_3/NaHCO_3$
10.0	10 mM $Na_2CO_3/NaHCO_3$
10.5	10 mM $Na_2CO_3/NaHCO_3$
11.0	10 mM $Na_2CO_3/NaHCO_3$
**PcaY_PP-LBD**	
3.0	10 mM citric acid/sodium citrate
3.5	10 mM citric acid/sodium citrate
4.0	10 mM citric acid/sodium citrate
5.0	10 mM citric acid/sodium citrate
6.0	3 mM Tris, 3 mM PIPES, 3 mM MES

7.0	3 mM Tris, 3 mM PIPES, 3 mM MES
8.0	3 mM Tris, 3 mM PIPES, 3 mM MES
9.0	3 mM Tris, 3 mM PIPES, 3 mM MES
10.0	10 mM $Na_2CO_3/NaHCO_3$
11.0	10 mM $Na_2CO_3/NaHCO_3$
11.5	10 mM KCl/NaOH
12.0	10 mM KCl/NaOH
**McpS-LBD**	
2.5	20 mM acetic acid/HCl
3.0	10 mM citric acid/sodium citrate
3.5	10 mM citric acid/sodium citrate
4.0	10 mM citric acid/sodium citrate
4.5	10 mM citric acid/sodium citrate
5.0	10 mM citric acid/sodium citrate
5.5	10 mM citric acid/sodium citrate
6.0	3 mM Tris, 3 mM PIPES, 3 mM MES
6.5	3 mM Tris, 3 mM PIPES, 3 mM MES
7.0	3 mM Tris, 3 mM PIPES, 3 mM MES
7.5	3 mM Tris, 3 mM PIPES, 3 mM MES
8.0	3 mM Tris, 3 mM PIPES, 3 mM MES
8.5	3 mM Tris, 3 mM PIPES, 3 mM MES
9.0	3 mM Tris, 3 mM PIPES, 3 mM MES
9.5	10 mM $Na_2CO_3/NaHCO_3$
10.0	10 mM $Na_2CO_3/NaHCO_3$
10.5	10 mM $Na_2CO_3/NaHCO_3$
**McpQ-LBD**	
3.5	10 mM acetic acid/ sodium acetate, 10 mM $MgCl_2$
4.0	10 mM acetic acid/ sodium acetate, 10 mM $MgCl_2$
5.0	10 mM acetic acid/ sodium acetate, 10 mM $MgCl_2$
6.0	3 mM Tris, 3 mM PIPES, 3 mM MES, 10 mM $MgCl_2$
7.0	3 mM Tris, 3 mM PIPES, 3 mM MES, 10 mM $MgCl_2$
8.0	3 mM Tris, 3 mM PIPES, 3 mM MES, 10 mM $MgCl_2$
9.0	3 mM Tris, 3 mM PIPES, 3 mM MES, 10 mM $MgCl_2$
10.0	10 mM $Na_2CO_3/NaHCO_3$, 10 mM $MgCl_2$
11.0	10 mM $Na_2CO_3/NaHCO_3$, 10 mM $MgCl_2$
11.5	10 mM KCl/NaOH, 10 mM $MgCl_2$
**Periplasmic solute binding proteins**	
**E6B08_RS28125**	
2.5	20 mM acetic acid/HCl
3.0	10 mM citric acid/sodium citrate
4.0	10 mM citric acid/sodium citrate
5.0	10 mM citric acid/sodium citrate
6.0	3 mM Tris, 3 mM PIPES, 3 mM MES
7.0	3 mM Tris, 3 mM PIPES, 3 mM MES
8.0	3 mM Tris, 3 mM PIPES, 3 mM MES
9.0	3 mM Tris, 3 mM PIPES, 3 mM MES
10.0	10 mM $Na_2CO_3/NaHCO_3$
11.0	10 mM $Na_2CO_3/NaHCO_3$
11.5	10 mM $Na_2HPO_4/NaOH$
**MBP**	
2.0	20 mM acetic acid/HCl
2.5	20 mM acetic acid/HCl
3.0	10 mM citric acid/sodium citrate

4.0	10 mM citric acid/sodium citrate
5.0	10 mM citric acid/sodium citrate
6.0	3 mM Tris, 3 mM PIPES, 3 mM MES
7.0	3 mM Tris, 3 mM PIPES, 3 mM MES
8.0	3 mM Tris, 3 mM PIPES, 3 mM MES
9.0	3 mM Tris, 3 mM PIPES, 3 mM MES
10.0	10 mM $Na_2CO_3/NaHCO_3$
11.0	10 mM $Na_2CO_3/NaHCO_3$
11.5	10 mM $Na_2HPO_4/NaOH$
**Cytosolic LBDs**	
**TodS-Nter**	
5.0	10 mM citric acid/sodium citrate, 2 mM DDT, 2 mM $MgCl_2$, 0.1 mM EDTA
5.5	10 mM citric acid/sodium citrate, 2 mM DDT, 2 mM $MgCl_2$, 0.1 mM EDTA
6.0	3 mM Tris, 3 mM PIPES, 3 mM MES, 2 mM DDT, 2 mM $MgCl_2$, 0.1 mM EDTA
6.5	3 mM Tris, 3 mM PIPES, 3 mM MES, 2 mM DDT, 2 mM $MgCl_2$, 0.1 mM EDTA
7.0	3 mM Tris, 3 mM PIPES, 3 mM MES, 2 mM DDT, 2 mM $MgCl_2$, 0.1 mM EDTA
7.5	3 mM Tris, 3 mM PIPES, 3 mM MES, 2 mM DDT, 2 mM $MgCl_2$, 0.1 mM EDTA
8.0	3 mM Tris, 3 mM PIPES, 3 mM MES, 2 mM DDT, 2 mM $MgCl_2$, 0.1 mM EDTA
8.5	3 mM Tris, 3 mM PIPES, 3 mM MES, 2 mM DDT, 2 mM $MgCl_2$, 0.1 mM EDTA
9.0	3 mM Tris, 3 mM PIPES, 3 mM MES, 2 mM DDT, 2 mM $MgCl_2$, 0.1 mM EDTA
9.5	10 mM $Na_2CO_3/NaHCO_3$, 2 mM DDT, 2 mM $MgCl_2$, 0.1 mM EDTA
10.0	10 mM $Na_2CO_3/NaHCO_3$, 2 mM DDT, 2 mM $MgCl_2$, 0.1 mM EDTA
**AdmX-LBD**	
7.0	3 mM Tris, 3 mM PIPES, 3 mM MES, 1 mM 2-mercaptoethanol
7.5	3 mM Tris, 3 mM PIPES, 3 mM MES, 1 mM 2-mercaptoethanol
8.0	3 mM Tris, 3 mM PIPES, 3 mM MES, 1 mM 2-mercaptoethanol
8.5	3 mM Tris, 3 mM PIPES, 3 mM MES, 1 mM 2-mercaptoethanol
**TtgV**	
4.5	10 mM citric acid/sodium citrate, 100 mM NaCl, 10 % (v/v) glycerol, 1 mM 2-mercaptoethanol, 8 mM magnesium acetate
5.0	10 mM citric acid/sodium citrate, 100 mM NaCl, 10 % (v/v) glycerol, 1 mM 2-mercaptoethanol, 8 mM magnesium acetate
6.0	3 mM Tris, 3 mM PIPES, 3 mM MES, 100 mM NaCl, 10 % (v/v) glycerol, 1 mM 2-mercaptoethanol, 8 mM magnesium acetate
7.0	3 mM Tris, 3 mM PIPES, 3 mM MES, 100 mM NaCl, 10 % (v/v) glycerol, 1 mM 2-mercaptoethanol, 8 mM magnesium acetate
8.0	3 mM Tris, 3 mM PIPES, 3 mM MES, 100 mM NaCl, 10 % (v/v) glycerol, 1 mM 2-mercaptoethanol, 8 mM magnesium acetate
9.0	3 mM Tris, 3 mM PIPES, 3 mM MES, 100 mM NaCl, 10 % (v/v) glycerol, 1 mM 2-mercaptoethanol, 8 mM magnesium acetate
10.0	10 mM $Na_2CO_3/NaHCO_3$, 100 mM NaCl, 10 % (v/v) glycerol, 1 mM 2-mercaptoethanol, 8 mM magnesium acetate
