# Supplementary material for: The pH Robustness of Bacterial Sensing
Source: mBio. 2022 Sep 26;13(5):e01650-22. doi: 10.1128/mbio.01650-22 (PMC9600550; doi:10.1128/mbio.01650-22)
Supplement: TABLE S2 [file mbio.01650-22-s0002.pdf]

Table S2. Dissociation constants derived from the microcalorimetric titrations of different proteins with ligands. SD: standard deviation. The pH optima are highlighted.

LBDs of periplasmic location, α/β folds										
	**CtpM-LBD**		**McpV-LBD**		**McpH-LBD**		**PctA-LBD**		**McpU-LBD**	
pH	K_D (μM)	SD	K_D (μM)	SD	K_D (μM)	SD	K_D (μM)	SD	K_D (μM)	SD
2.5										
3.0							1.33	0.3		
3.5					0.80	0.2	0.86	0.2		
4.0					6.85	1	1.38	0.3		
4.5	147	122					0.69	0.1	78.8	21
5.0	136	32	55.3	5	**1.68**	**0.3**	0.98	0.1	9.90	0.2
5.5	49.7	3					**0.59**	**0.1**	3.70	0.1
6.0	39.5	2	119	9	2.61	0.4	0.87	0.1	1.87	0.1
6.5	**38.7**	**2**					0.70	0.1	1.51	0.1
7.0	44.0	2	**33.1**	**3**	1.91	0.1	1.82	0.2	1.07	0.1
7.5	80.0	6					1.31	0.1		
8.0	55.0	2	84.7	7	1.86	0.2	0.87	0.1	0.93	0.1
8.5	86.4	10					1.32	0.2	**0.70**	**0.1**
9.0	145	20	53.8	3	3.61	0.1	0.92	0.1	0.80	0.1
9.5							1.20	0.1	1.22	0.1
10.0			70.4	9	35.7	2	1.27	0.1	2.35	0.1
10.5							1.50	0.8	5.11	0.1
11.0			79.4	6			3.88	0.5	13.3	0.1
11.5			641	68			46.6	7	89.2	31

LBDs of periplasmic location, four-helix bundle folds										
	**Tar-LBD**		**PcaY_PP-LBD**		**McpS-LBD**		**McpQ-LBD**			
pH	K_D (μM)	SD	K_D (μM)	SD	K_D (μM)	SD	K_D (μM)	SD		
2.5										
3.0	19.4	3			131	90				
3.5	1.80	0.1	95.4	5	113	32				
4.0	0.71	0.1	5.18	0.2	143	50				
4.5	0.46	0.1			42.0	6				
5.0	**0.40**	**0.1**	**1.22**	**0.1**	50.0	7	840	59		
5.5	0.98	0.1			**4.00**	**0.4**				
6.0	0.96	0.1	2.03	0.2	5.70	0.5	**153**	**5**		
6.5	1.10	0.1			5.20	0.9				
7.0	1.64	0.1	1.52	0.3	20.0	1	308	59		
7.5	2.20	0.1			21.1	1				
8.0	3.20	0.1	4.78	0.4	14.4	0.4	505	28		
8.5	3.30	0.2			13.0	0.3				
9.0	3.90	0.2	8.77	0.3	14.4	0.7	699	42		
9.5	4.70	0.2			15.6	2				
10.0	16.1	2	12.2	1	12.5	9	406	30		
10.5	40.7	20								
11.0			86.2	9			401	73		
11.5			510	53						

Periplasmic solute binding proteins				
	MBP		E6B08_RS28125	
pH	K_D (μM)	SD	K_D (μM)	SD
2.5	26.3	1		
3.0	11.0	0.6	15.4	1
3.5				
4.0	3.05	0.2	6.02	1
4.5				
5.0	1.67	0.1	20.9	4
5.5				
6.0	2.65	0.1	1.47	0.2
6.5				
7.0	1.71	0.1	1.28	0.1
7.5				
8.0	1.20	0.1	0.98	0.1
8.5				
9.0	**0.63**	**0.1**	**0.66**	**0.1**
9.5				
10.0	1.00		1.69	0.1
10.5				
11.0	1.00		7.24	0.2
11.5				

LBDs of cytosolic location						
	TodS-Nter		AdmX-LBD		TtgV	
pH	K_D (μM)	SD	K_D (μM)	SD	K_D (μM)	SD
2.5						
3.0						
3.5						
4.0						
4.5						
5.0					826	153
5.5	0.35	0.1				
6.0	0.26	0.1			**67.6**	**5**
6.5	**0.25**	**0.1**				
7.0	0.26	0.1			75.8	3
7.5	0.53	0.1	22.0	5		
8.0	0.79	0.1	**12.7**	**2**	113	6
8.5	1.50	0.2				
9.0	2.40	0.3			102	15
9.5	1.90	0.3				
10.0						
10.5						
11.0						
11.5						
