# Supplementary material for: The pH Robustness of Bacterial Sensing
Source: mBio. 2022 Sep 26;13(5):e01650-22. doi: 10.1128/mbio.01650-22 (PMC9600550; doi:10.1128/mbio.01650-22)
Supplement: FIG S1 [file mbio.01650-22-s0005.pdf]

Fig S1. Microcalorimetric titrations of mono- and bimodular α/β fold ligand binding domains with different ligands at different pH.

L-malate recognition by CtpM-LBD

pH 4.5

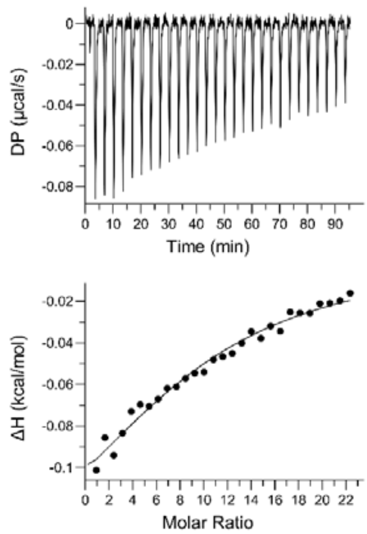

pH 5

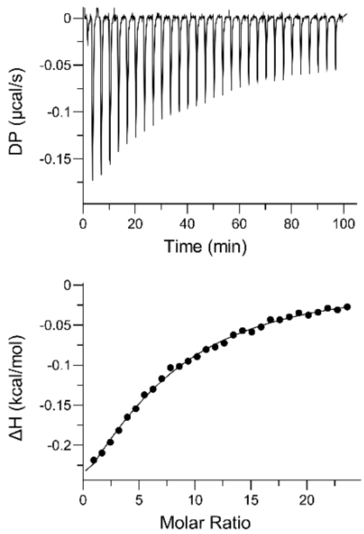

pH 5.5

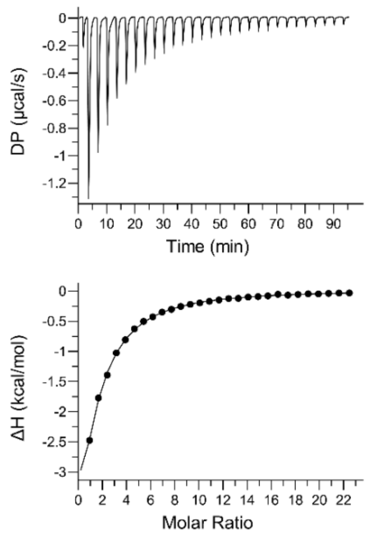

pH 6

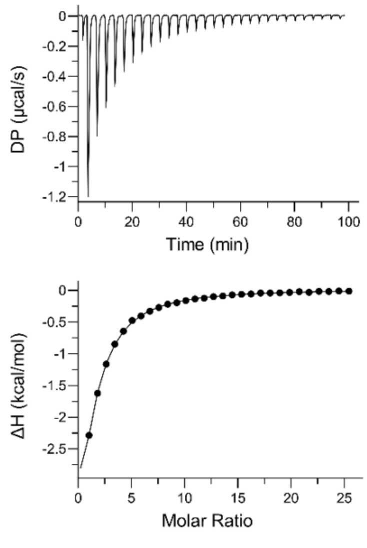

pH 6.5

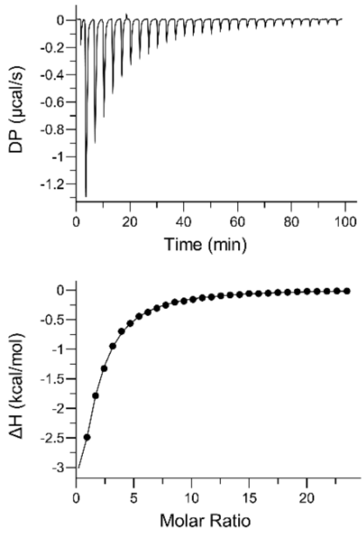

pH 7

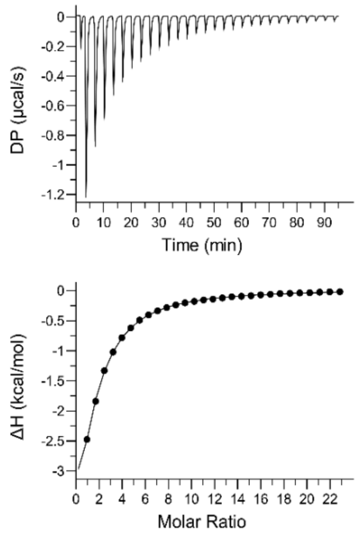

pH 7.5

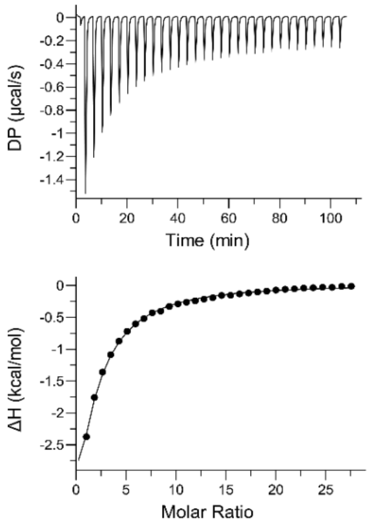

pH 8

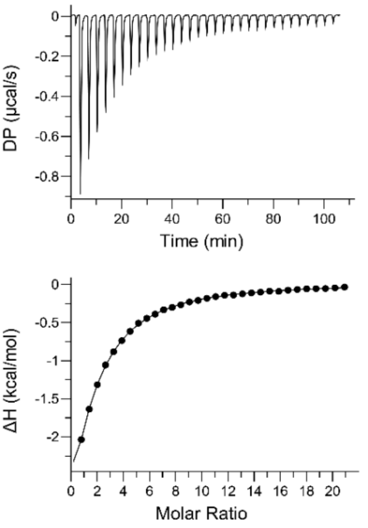

pH 8.5

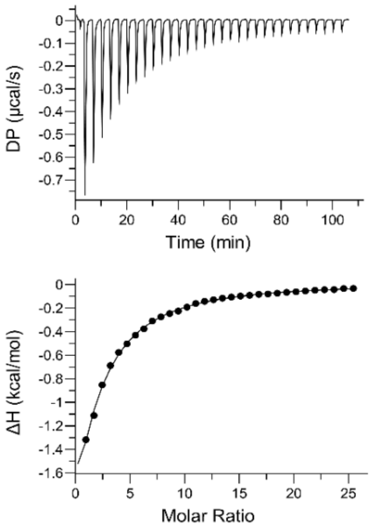

<table>
<tr><th>pH 9</th><th>pH 9.5</th></tr>
<tr><td>
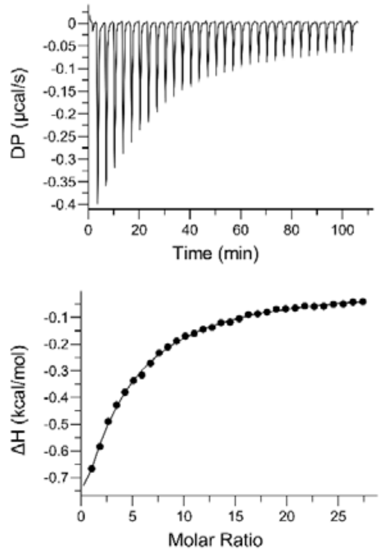

</td><td>
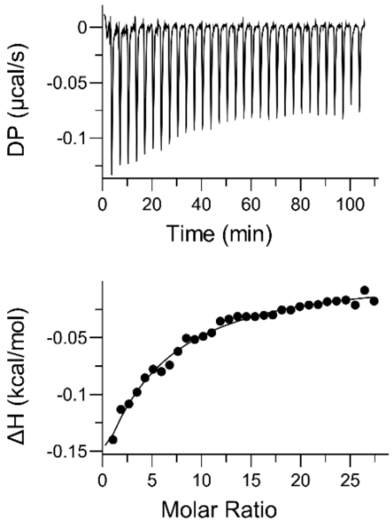

</td></tr>
</table>

Propionate recognition by McpV-LBD

pH 5.0

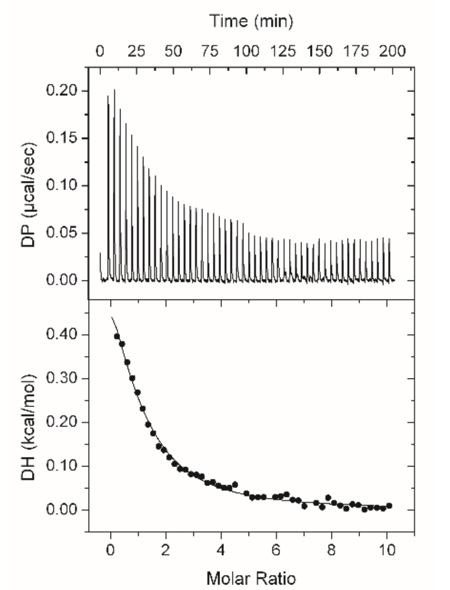

pH 6.0

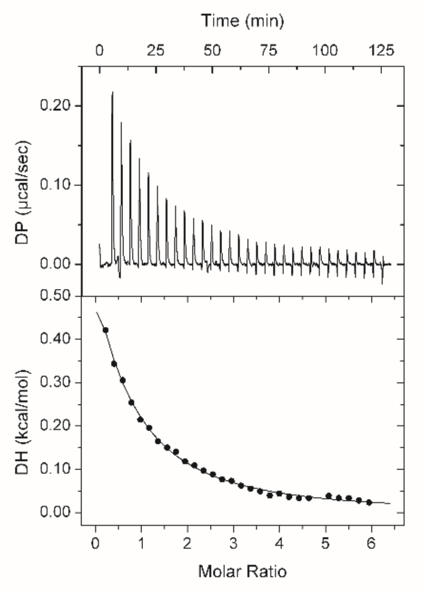

pH 7.0

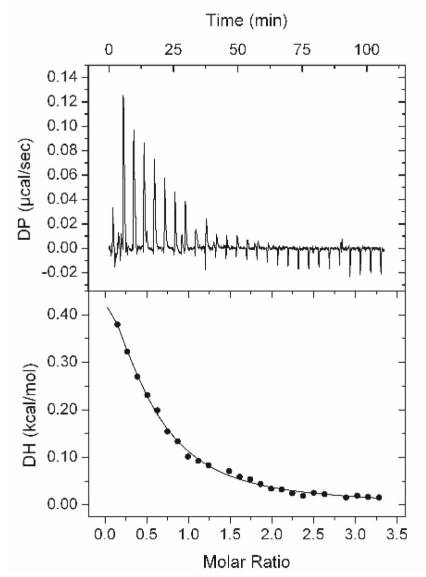

pH 8.0

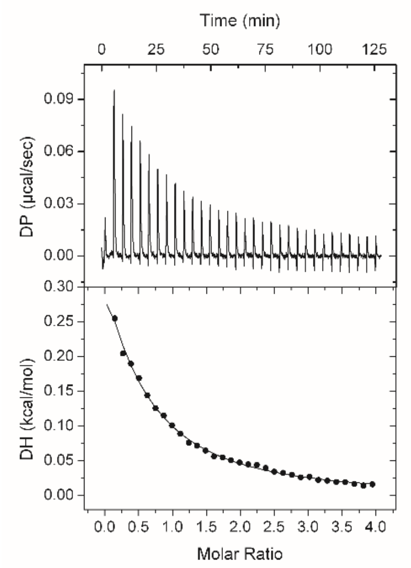

pH 9.0

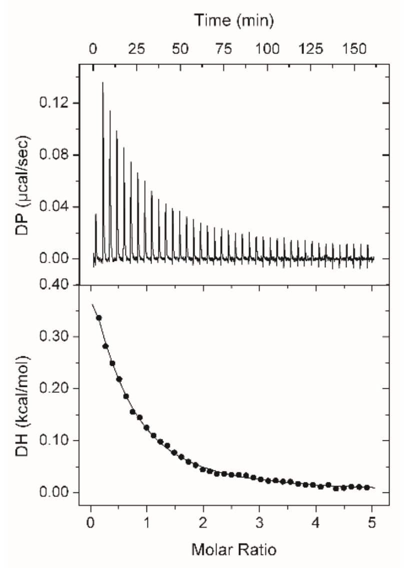

pH 10.0

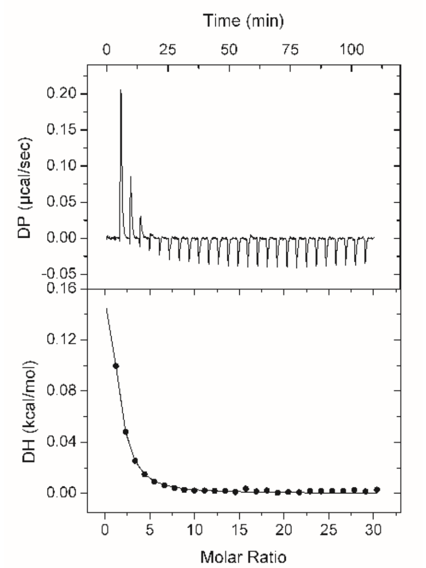

pH 11.0

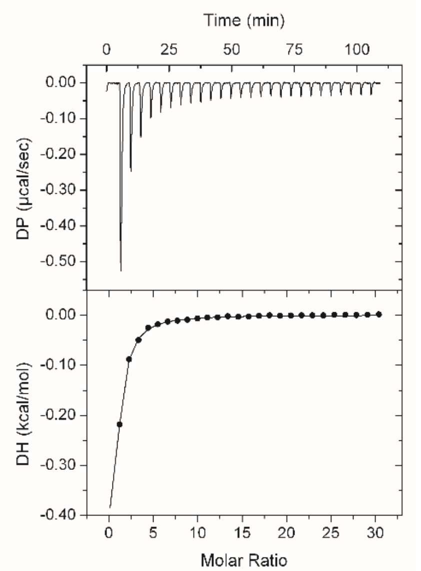

pH 11.5

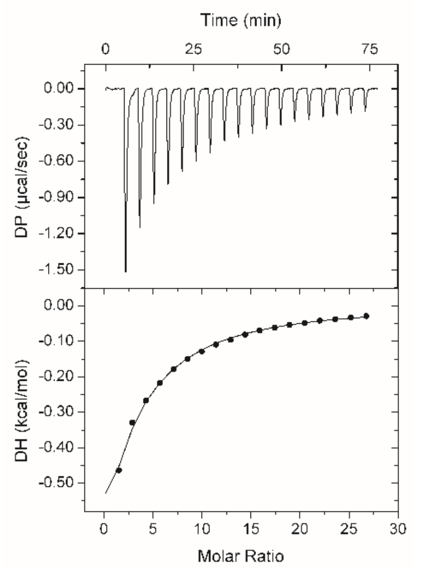

L-alanine recognition by PctA-LBD

pH 3.0

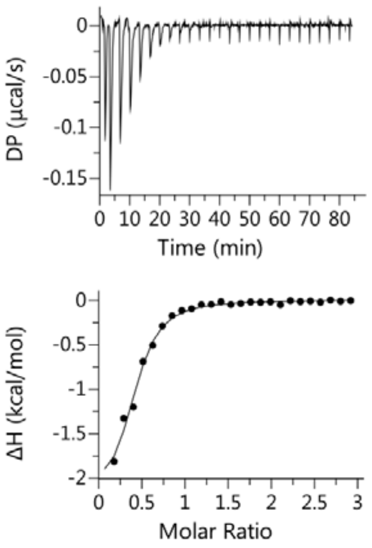

pH 3.5

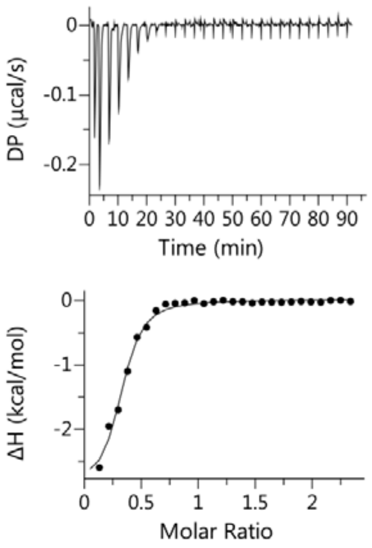

pH 4.0

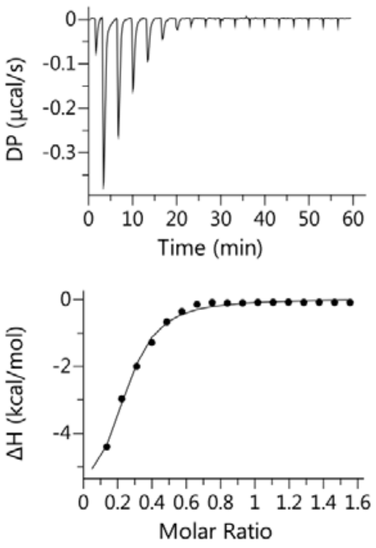

pH 4.5

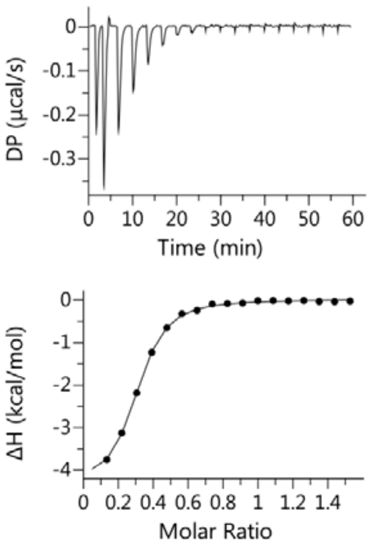

pH 5.0

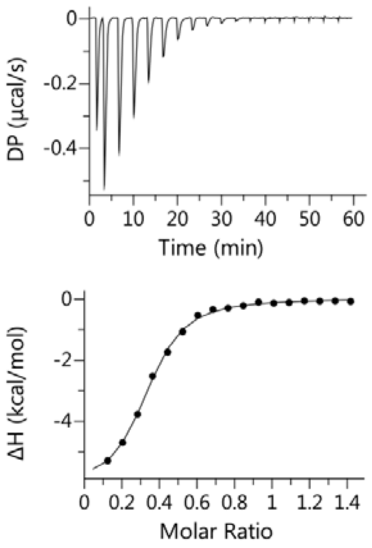

pH 5.5

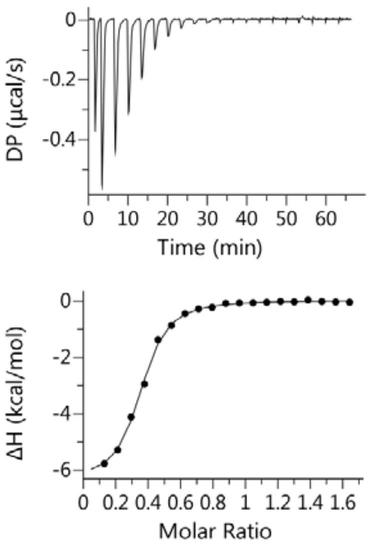

pH 6.0

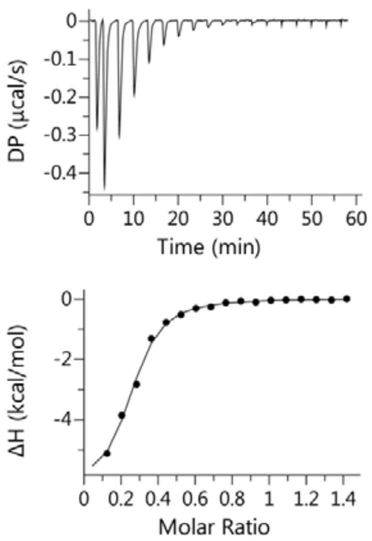

pH 6.5

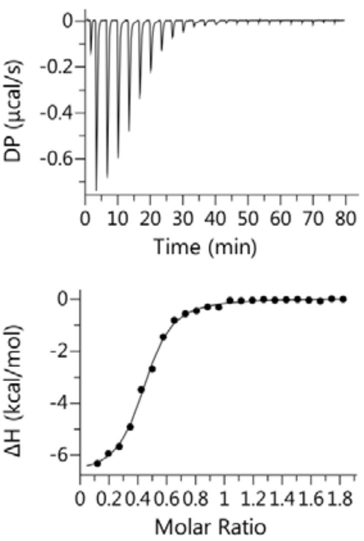

pH 7.0

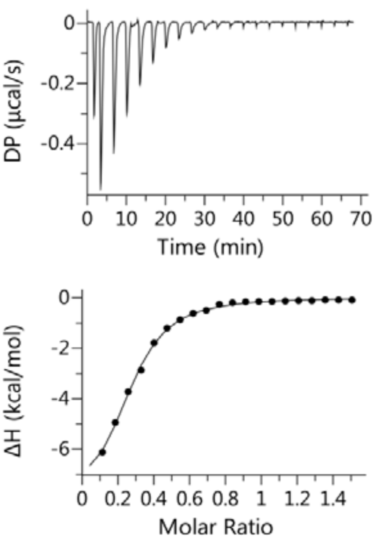

pH 7.5

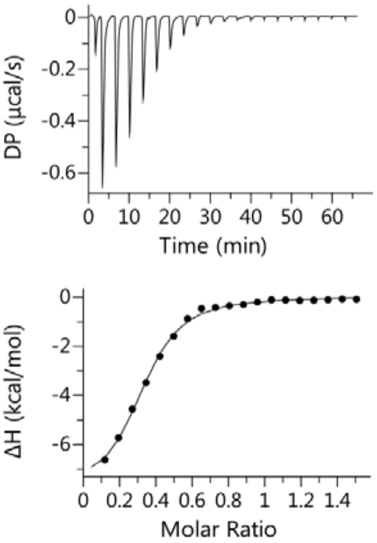

pH 8.0

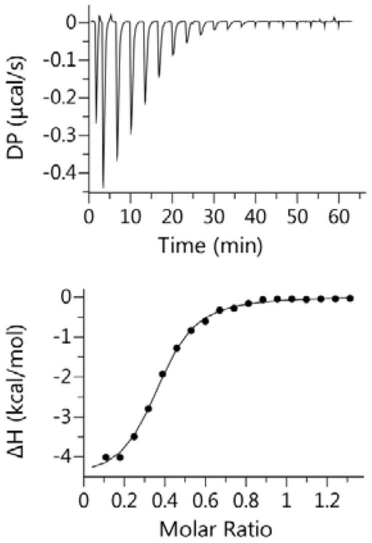

pH 8.5

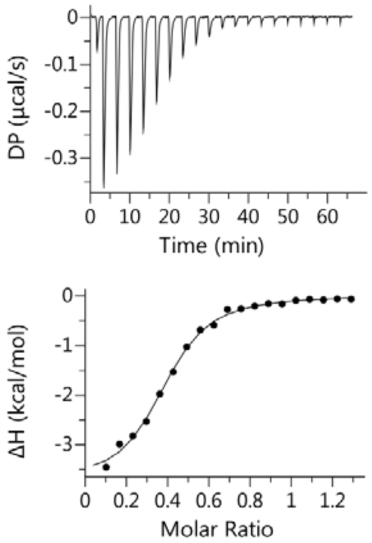

pH 9.0

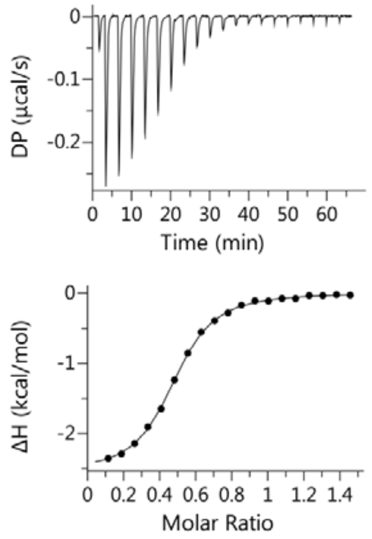

pH 9.5

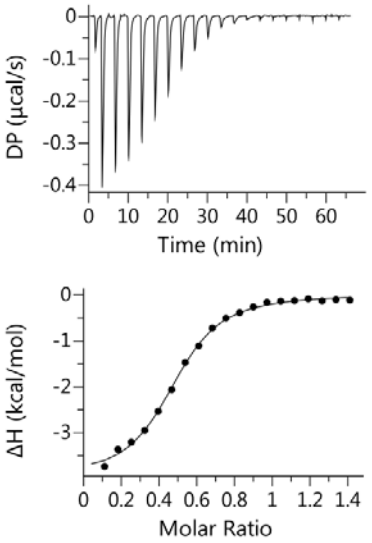

pH 10.0

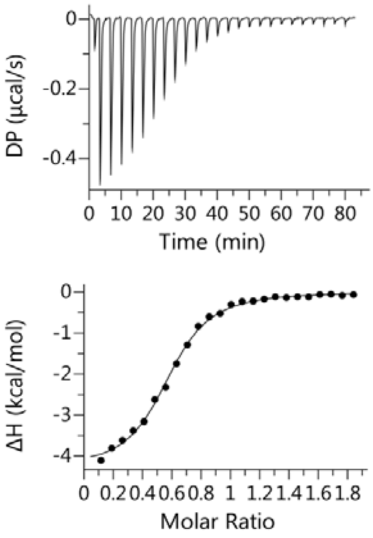

pH 10.5

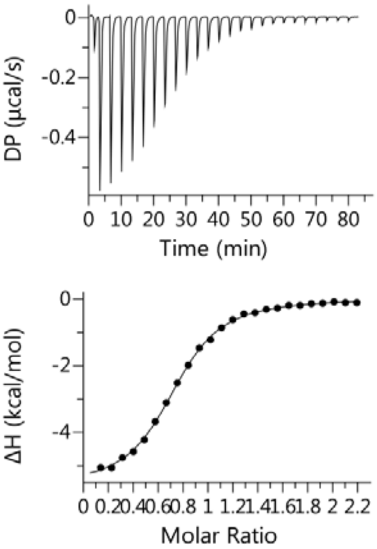

pH 11.0

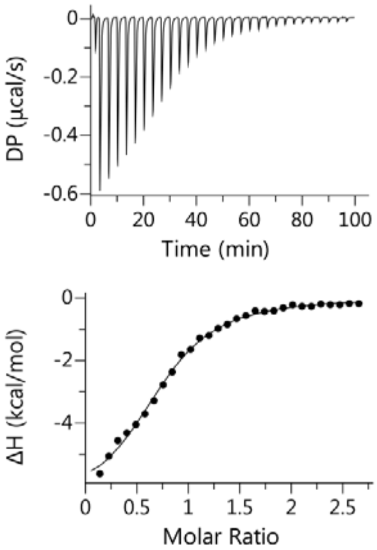

pH 11.5

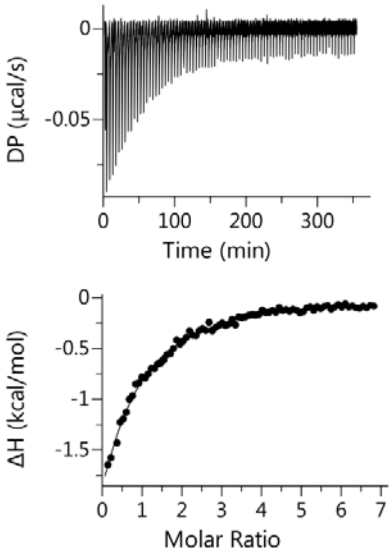

Adenine recognition by McpH-LBD

pH 3.5

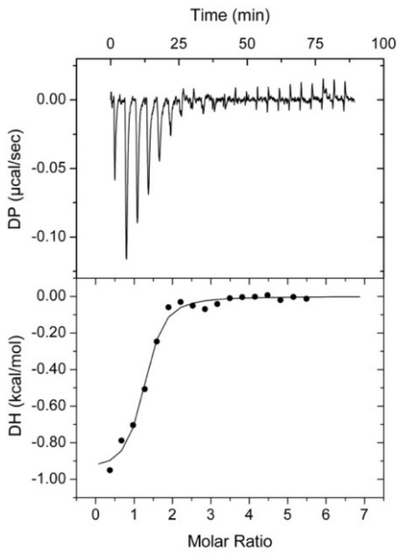

pH 4.0

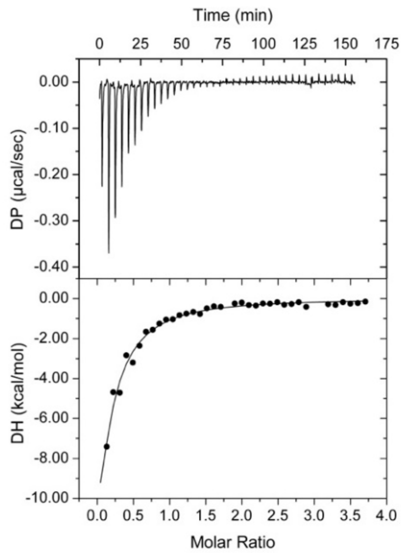

pH 5.0

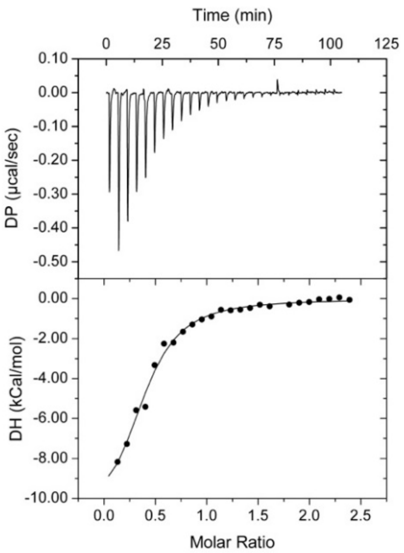

pH 6.0

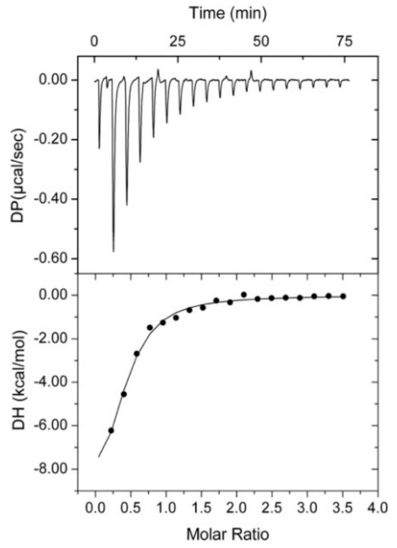

pH 7.0

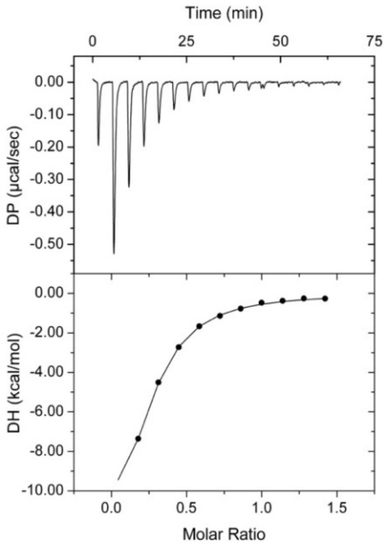

pH 8.0

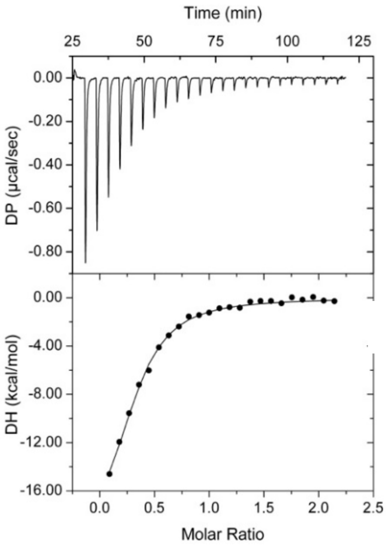

pH 9.0

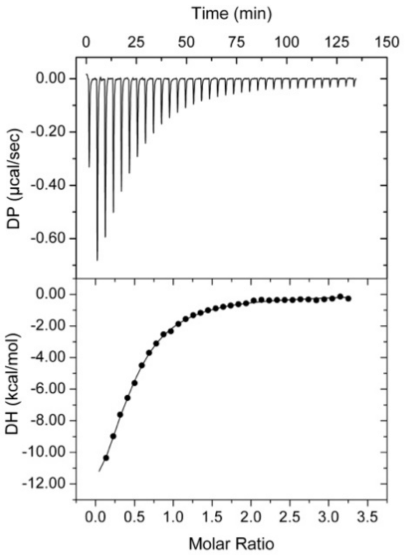

pH 10.0

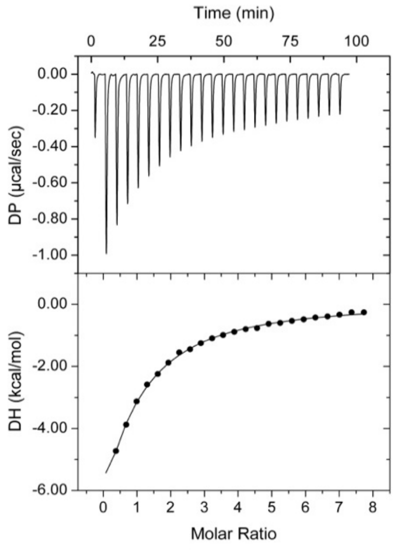

Putrescine recognition by McpU-LBD

pH 4.5	pH 5.0	pH 5.5
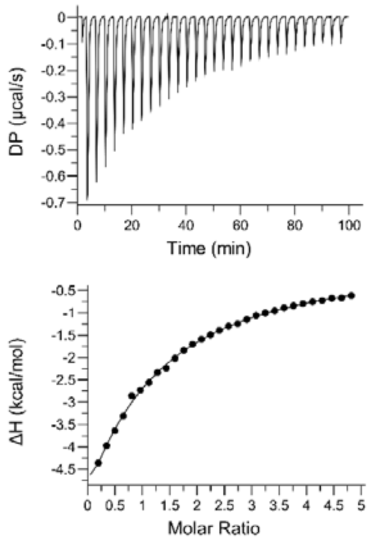	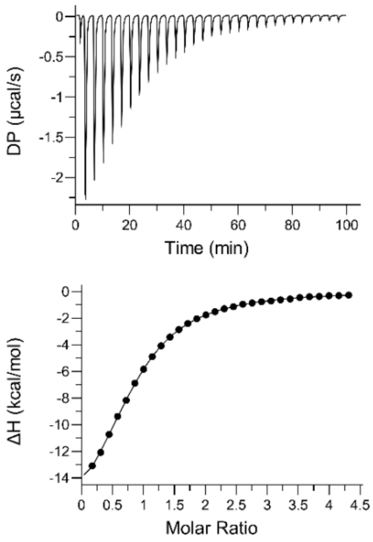	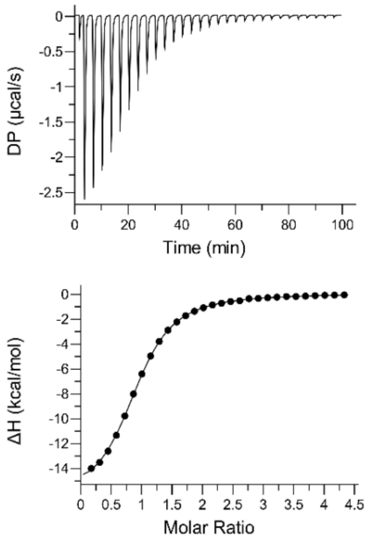
pH 6.0	pH 6.5	pH 7.0
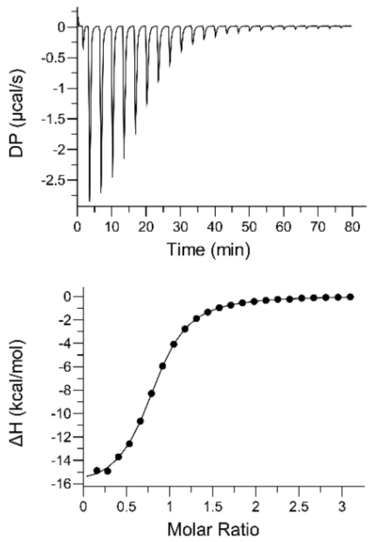	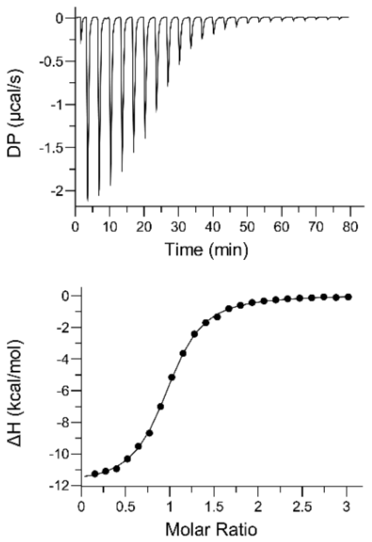	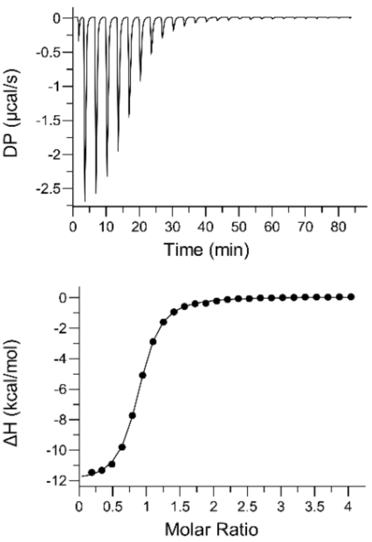
pH 8.0	pH 8.5	pH 9.0
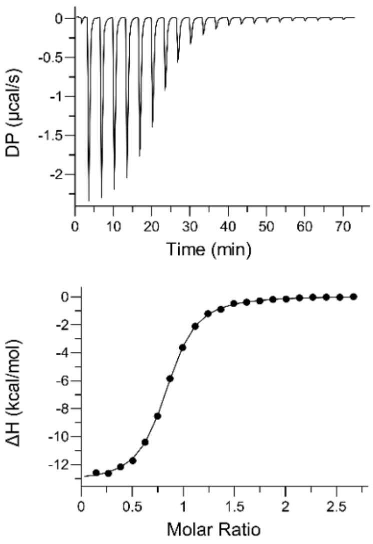	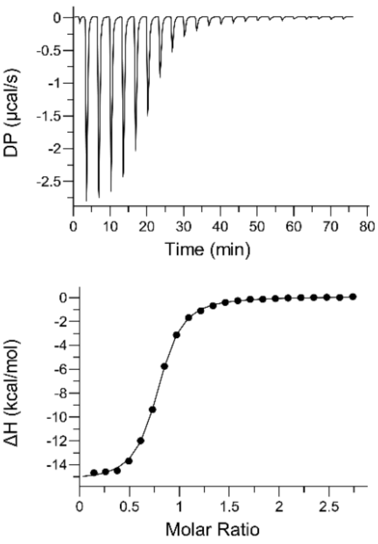	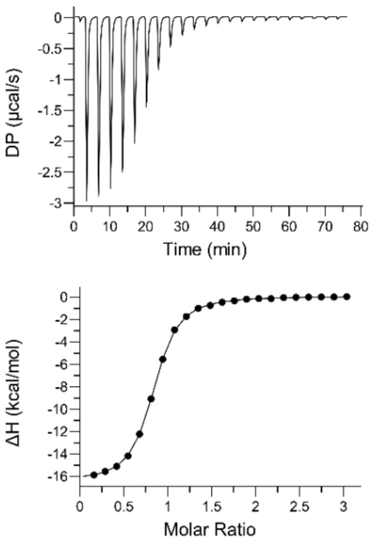

<table>
<tr><th>pH 9.5</th><th>pH 10.0</th><th>pH 10.5</th></tr>
<tr><td>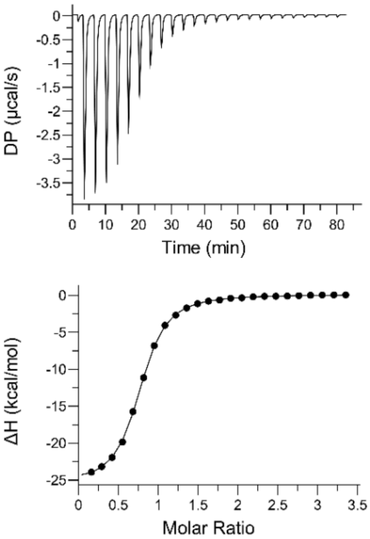
</td><td>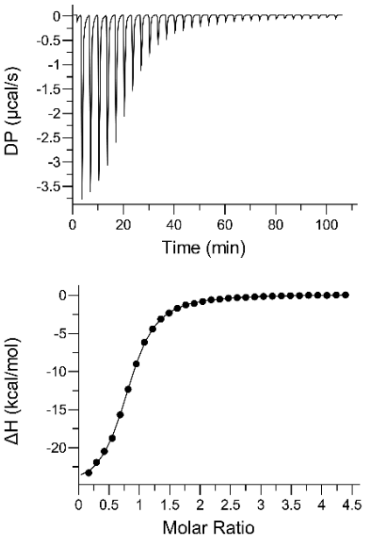
</td><td>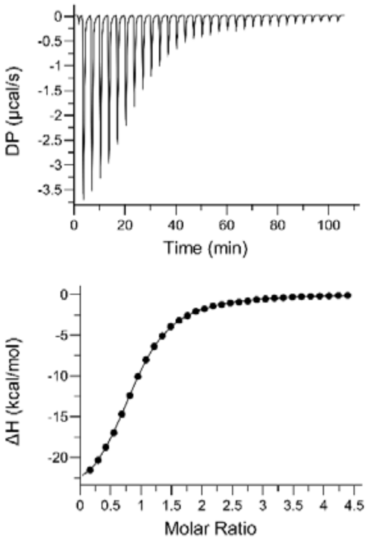
</td></tr>
<tr><th>pH 11.0</th><th>pH 11.5</th><th></th></tr>
<tr><td>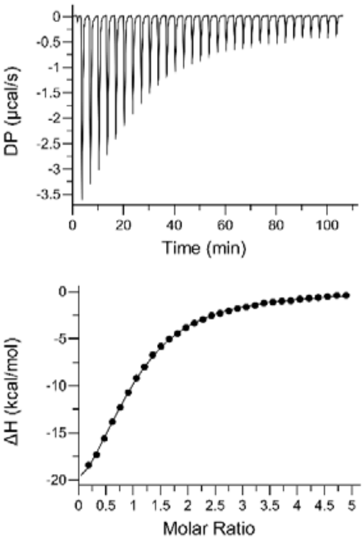
</td><td>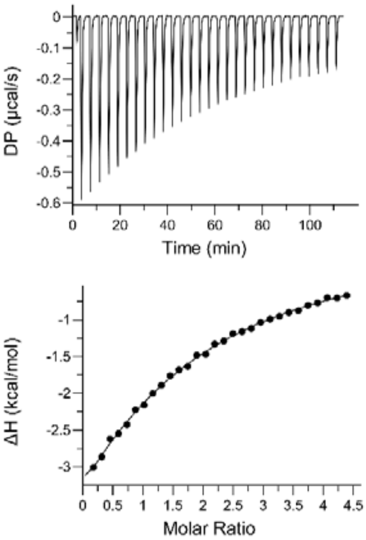
</td><td></td></tr>
</table>
