# Supplementary material for: The pH Robustness of Bacterial Sensing
Source: mBio. 2022 Sep 26;13(5):e01650-22. doi: 10.1128/mbio.01650-22 (PMC9600550; doi:10.1128/mbio.01650-22)
Supplement: FIG S2 [file mbio.01650-22-s0006.pdf]

Fig S2. Microcalorimetric titrations of mono- and bimodular four-helix bundle fold ligand binding domains with different ligands at different pH.

L-aspartate recognition by Tar-LBD

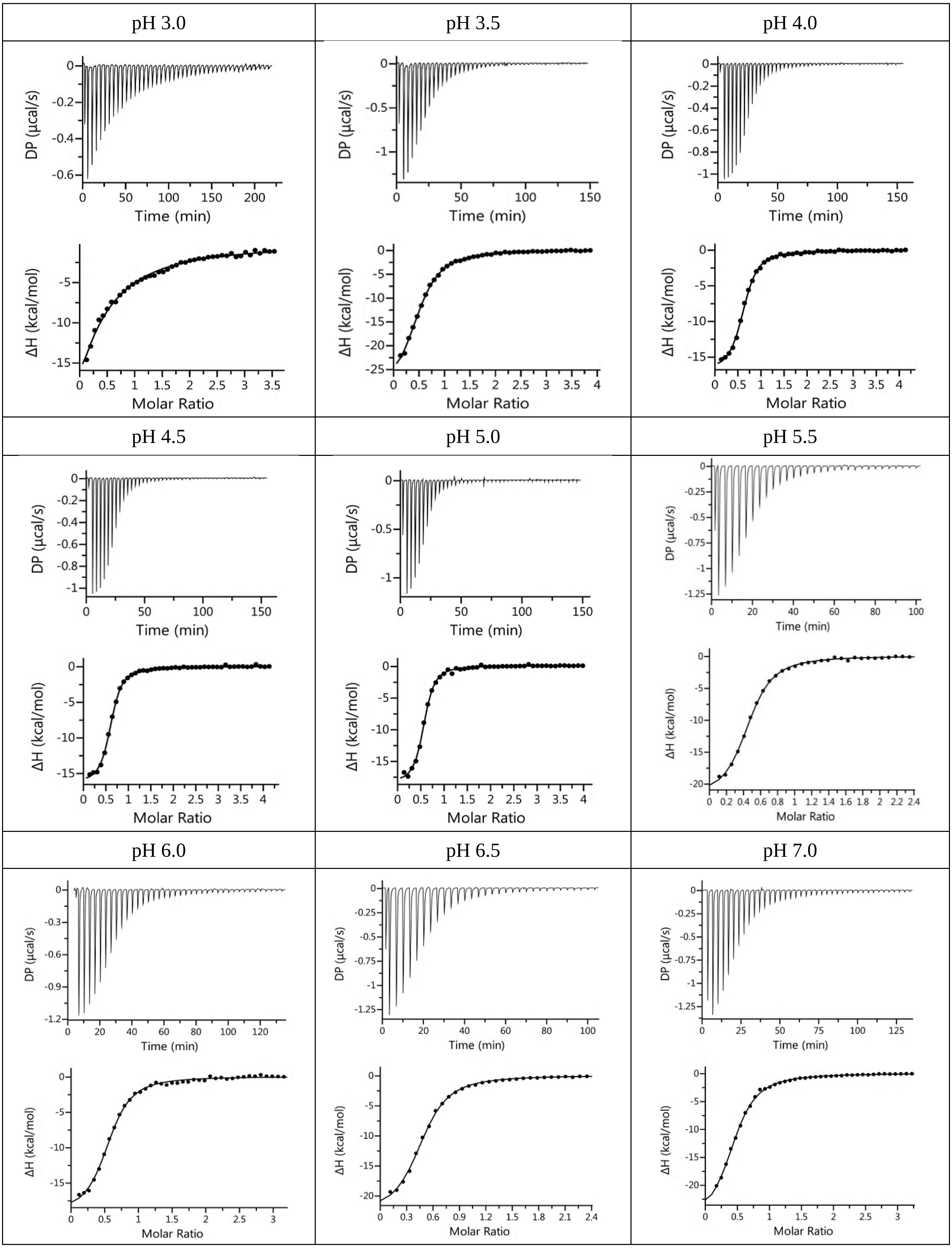

pH 7.5

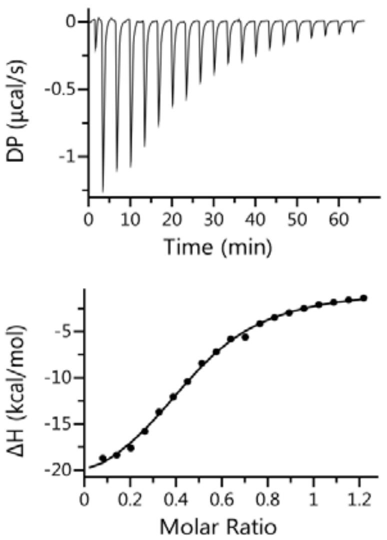

pH 8.0

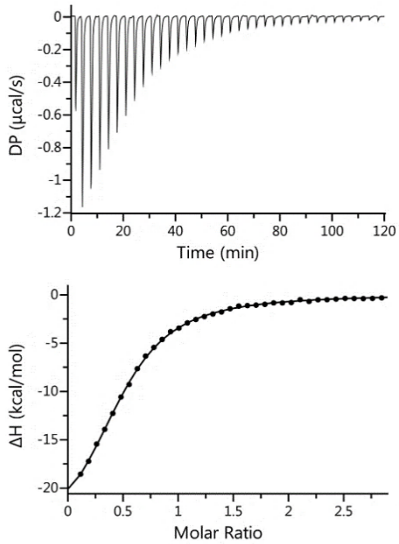

pH 8.5

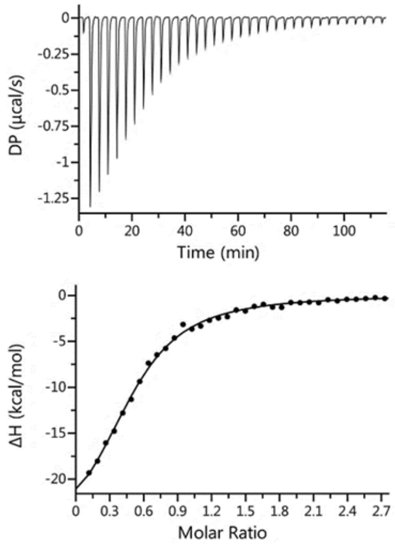

pH 9.0

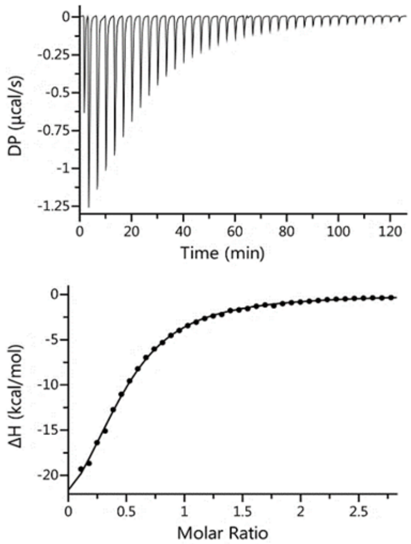

pH 9.5

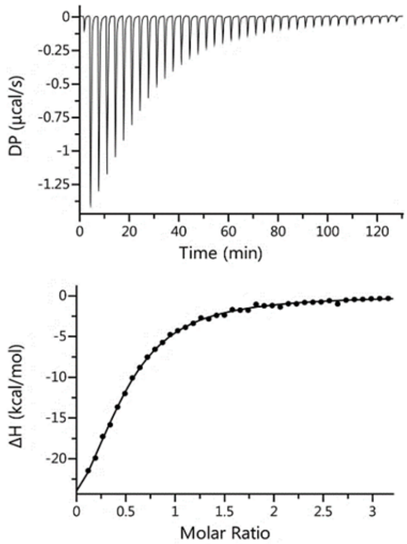

pH 10.0

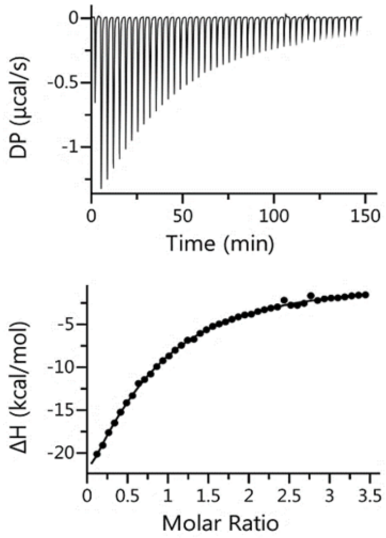

pH 10.5

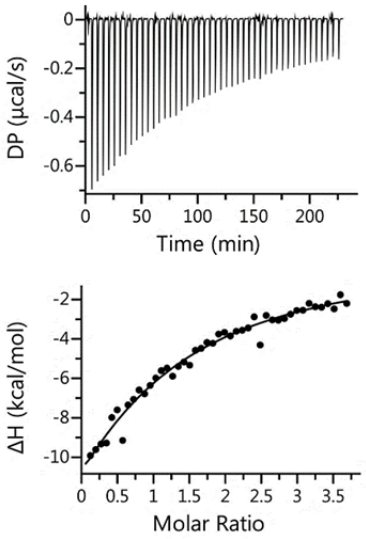

Quinate recognition by PcaY_PP-LBD

pH 3.5

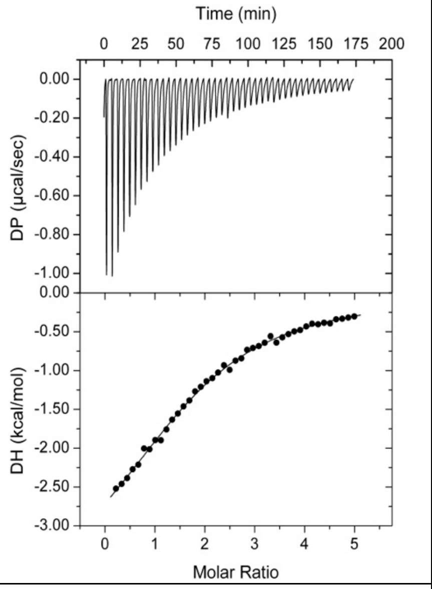

pH 4.0

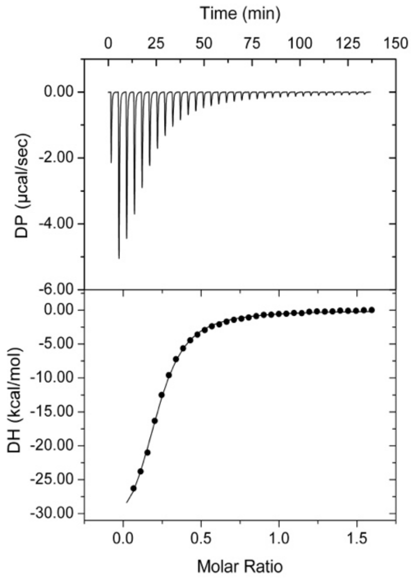

pH 5.0

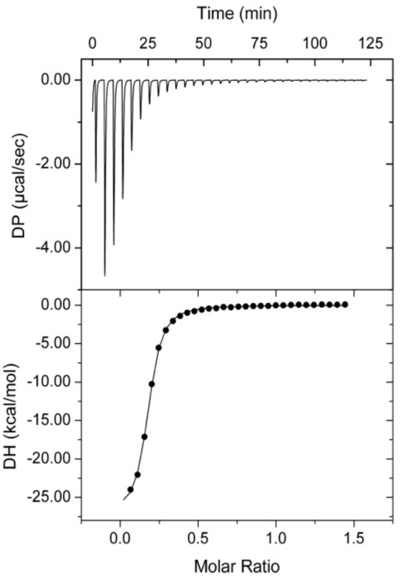

pH 6.0

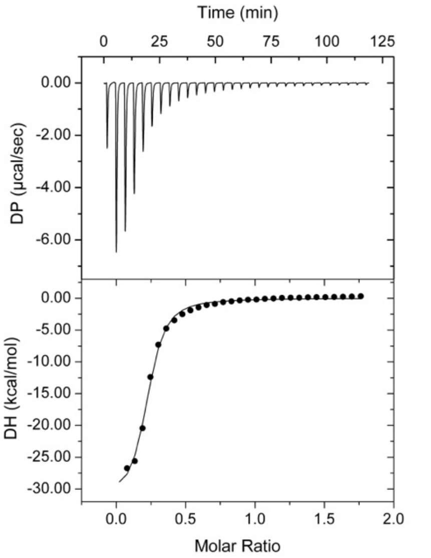

pH 7.0

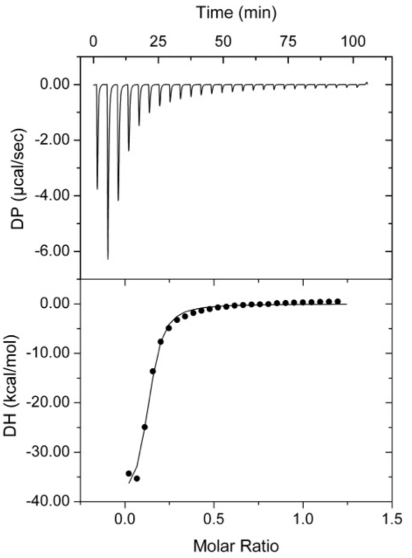

pH 8.0

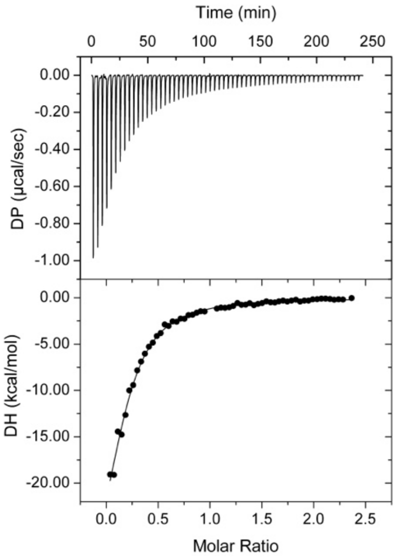

pH 9.0

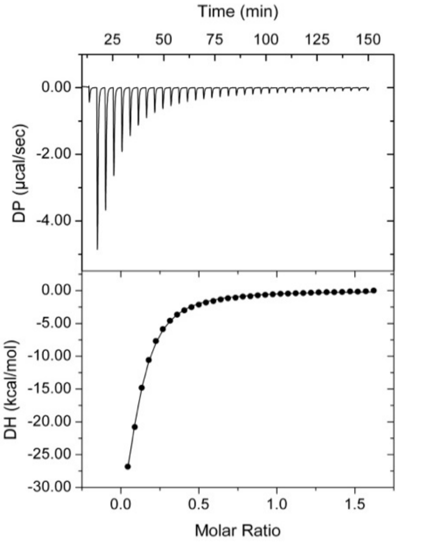

pH 10.0

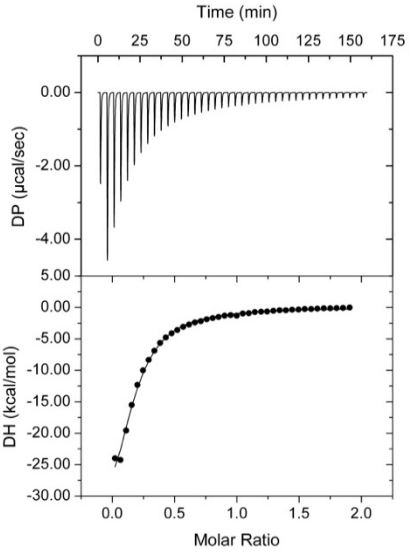

pH 11.0

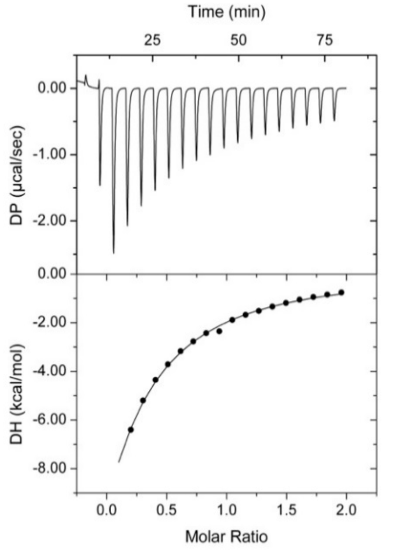

pH 11.5
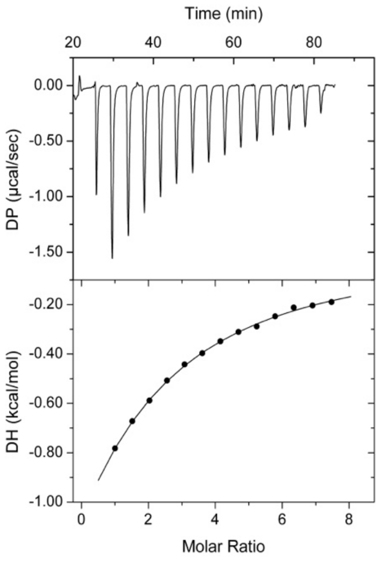

Time (min)
20
40
60
80
0.00
-0.50
-1.00
-1.50
DP (µcal/sec)
-0.20
-0.40
-0.60
-0.80
-1.00
DH (kcal/mol)
0
2
4
6
8
Molar Ratio

L-malate recognition by McpS-LBD

pH 3.0

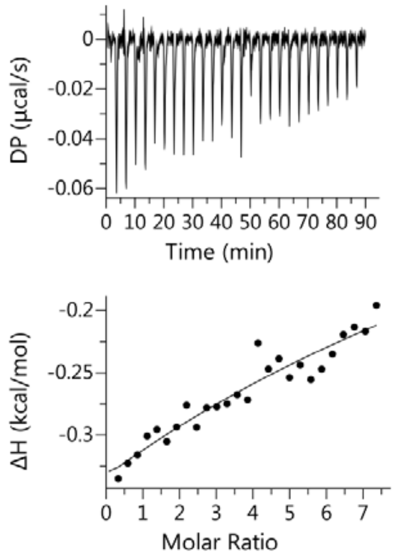

pH 3.5

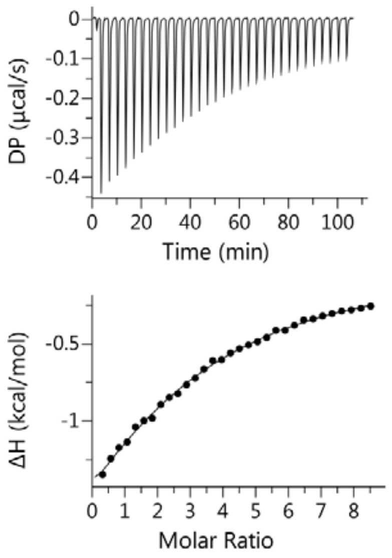

pH 4.0

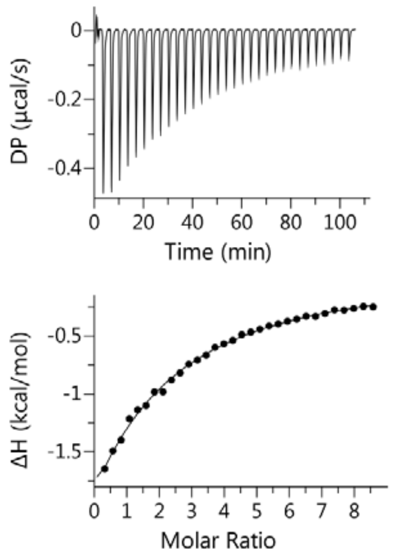

pH 4.5

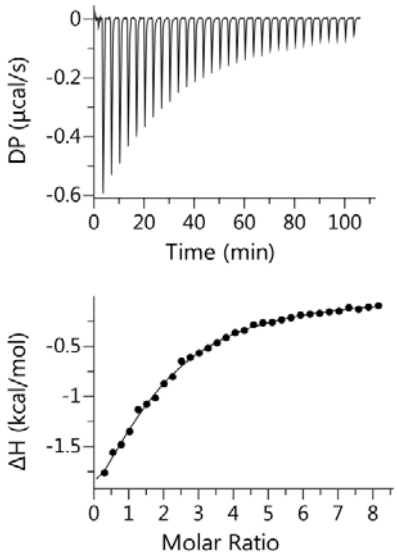

pH 5.0

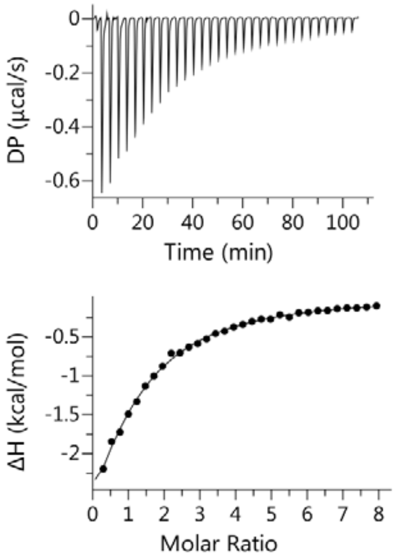

pH 5.5

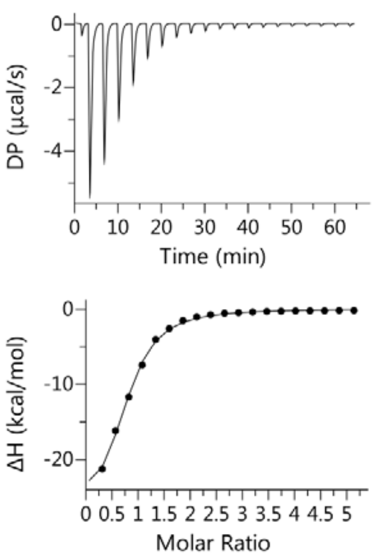

pH 6.0

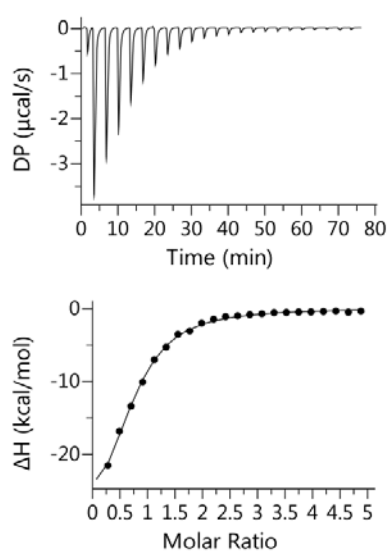

pH 6.5

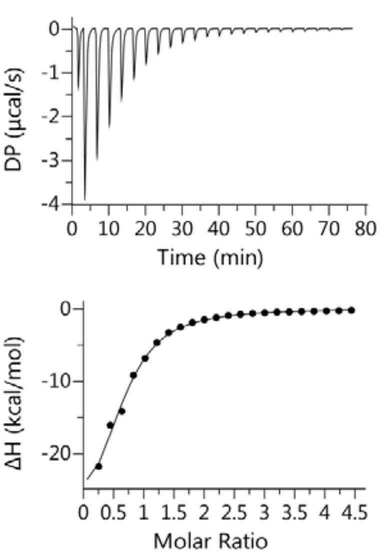

pH 7.0

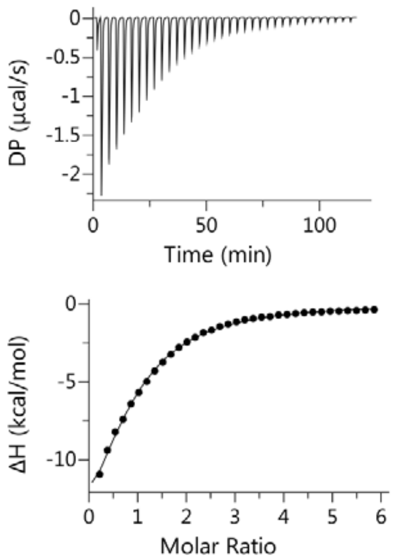

pH 7.5

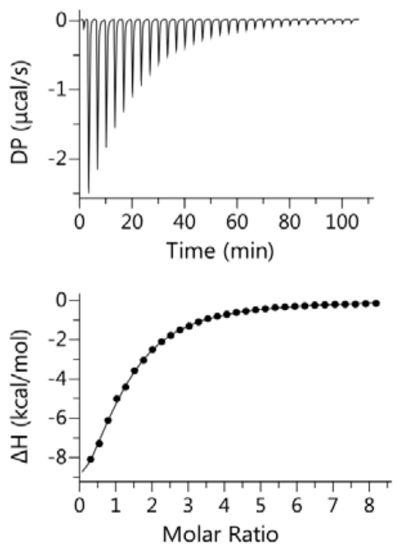

pH 8.0

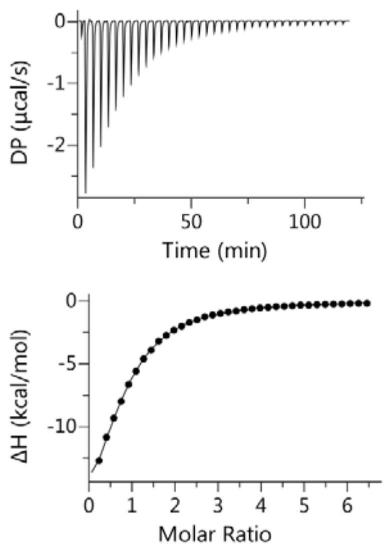

pH 8.5

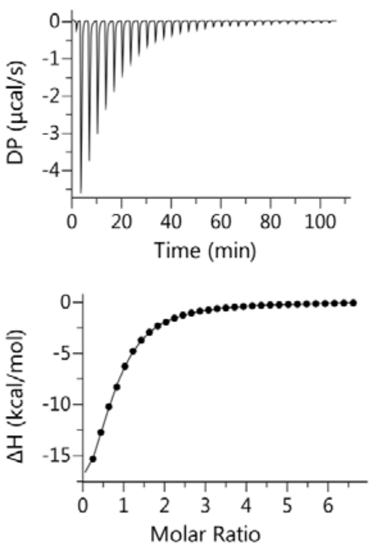

pH 9.0

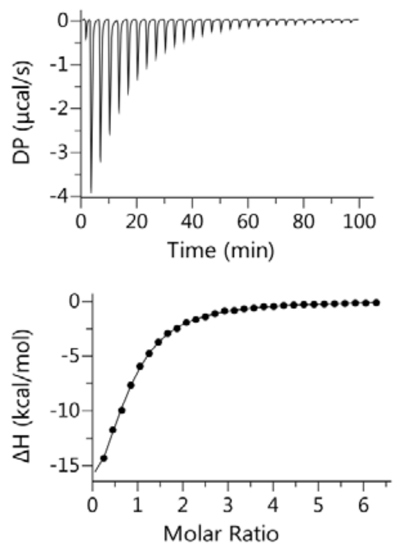

pH 9.5

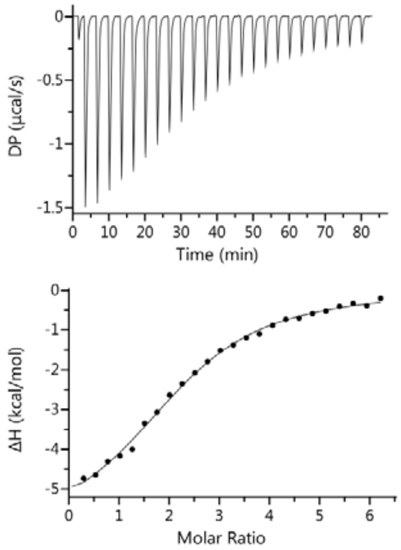

pH 10.0

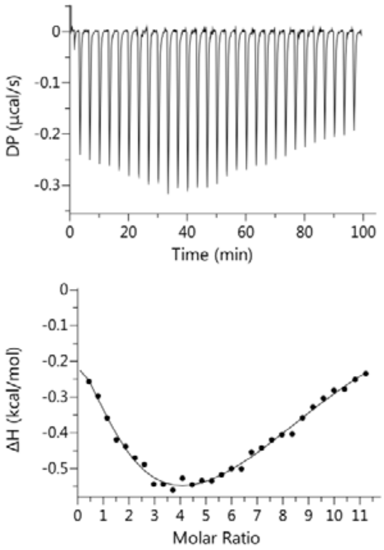

Citrate recognition by McpQ-LBD

<table>
<tr><td>pH 4.0</td><td>pH 5.0</td><td>pH 6.0</td></tr>
<tr><td>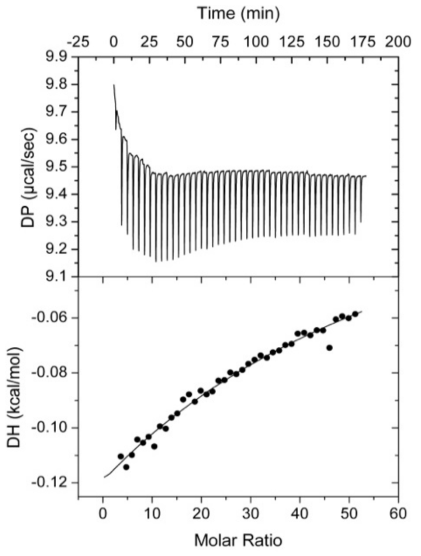
</td><td>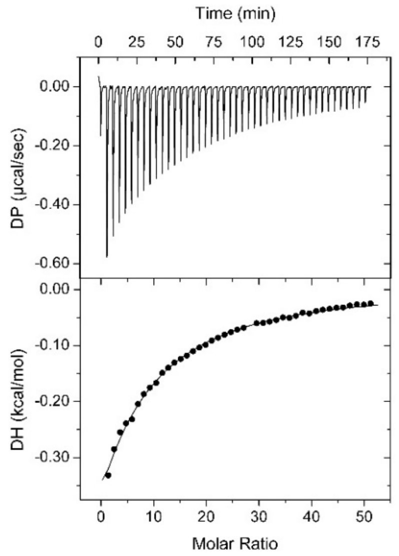
</td><td>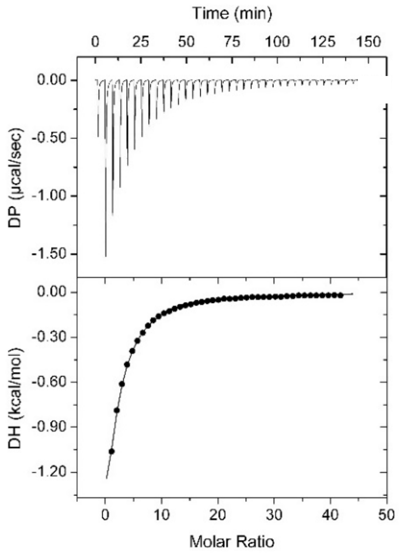
</td></tr>
<tr><td>pH 7.0</td><td>pH 8.0</td><td>pH 9.0</td></tr>
<tr><td>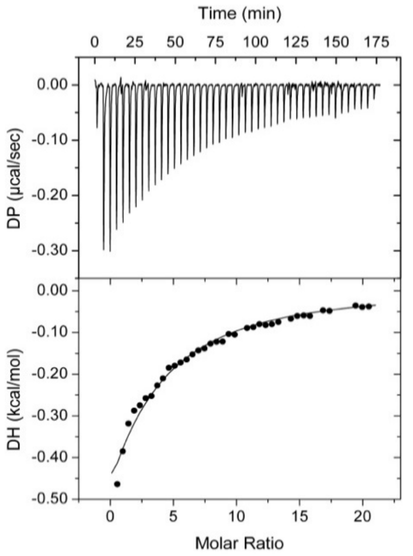
</td><td>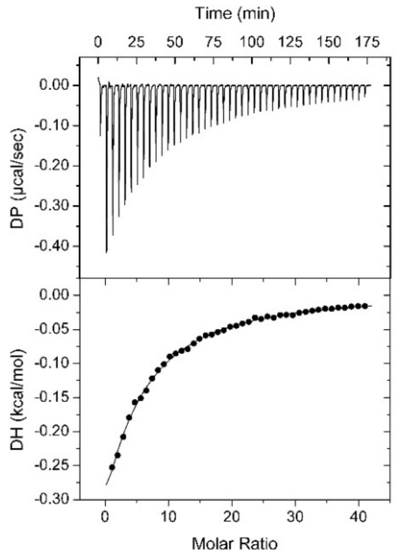
</td><td>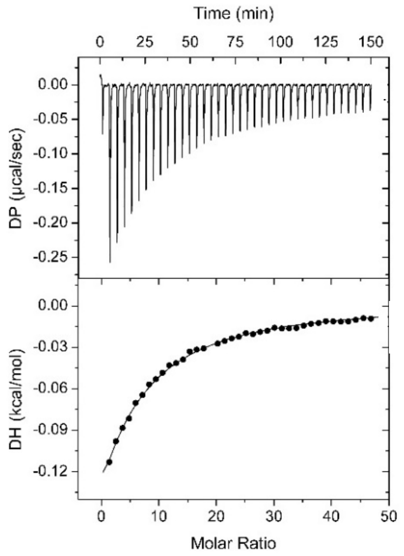
</td></tr>
<tr><td>pH 10.0</td><td>pH 11.0</td><td></td></tr>
<tr><td>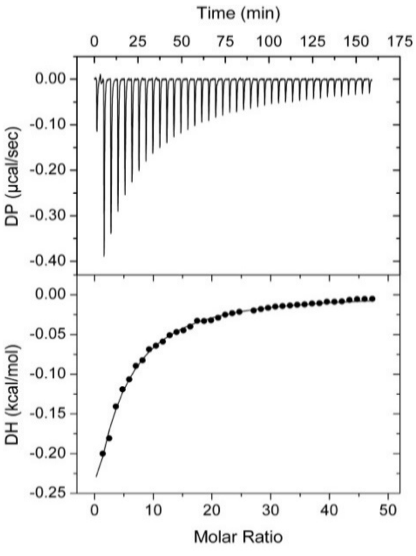
</td><td>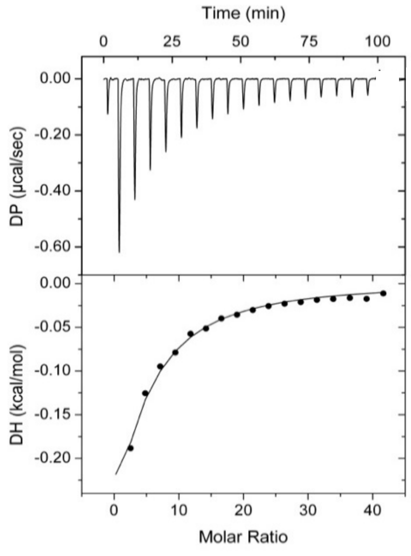
</td><td></td></tr>
</table>
