# Supplementary material for: The pH Robustness of Bacterial Sensing
Source: mBio. 2022 Sep 26;13(5):e01650-22. doi: 10.1128/mbio.01650-22 (PMC9600550; doi:10.1128/mbio.01650-22)
Supplement: FIG S3 [file mbio.01650-22-s0007.pdf]

Fig. S3. Microcalorimetric titrations of solute binding proteins and cytosolic ligand-binding domains with ligands at different pH.

D-maltose recognition by the maltose binding protein (MBP)

pH 2.5

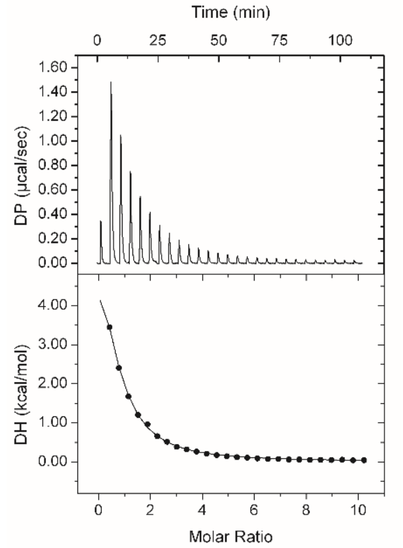

pH 3.0

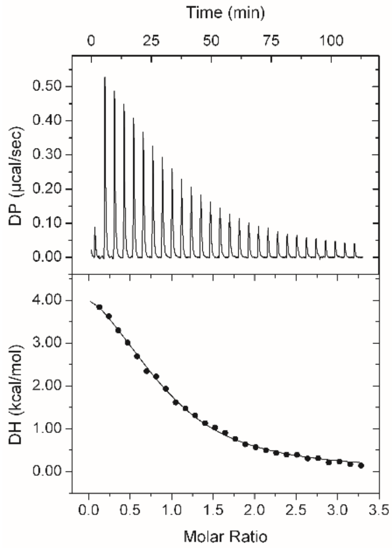

pH 4.0

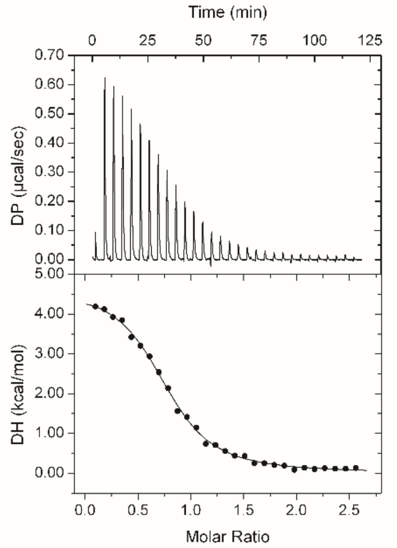

pH 5.0

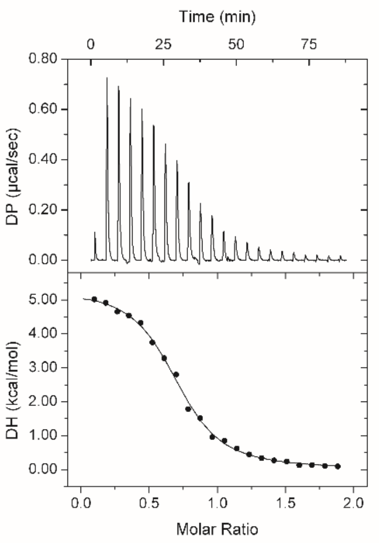

pH 6.0

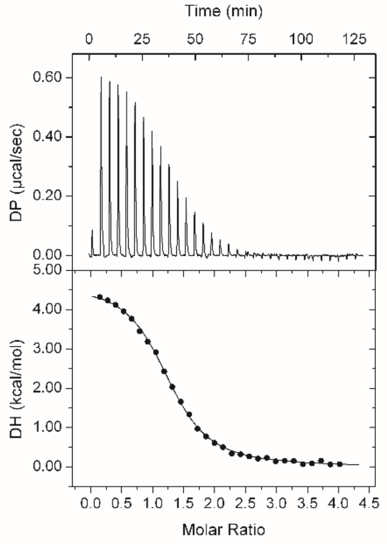

pH 7.0

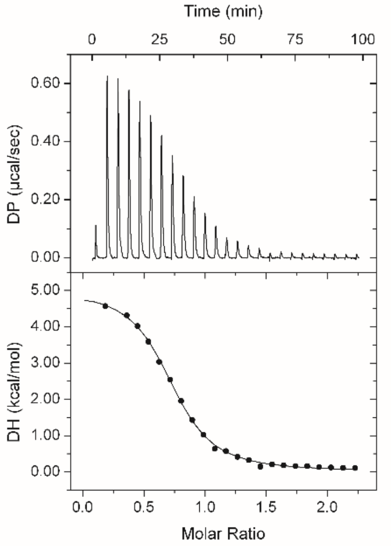

pH 8.0

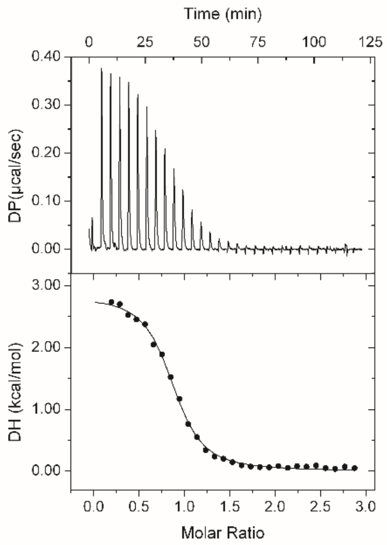

pH 9.0

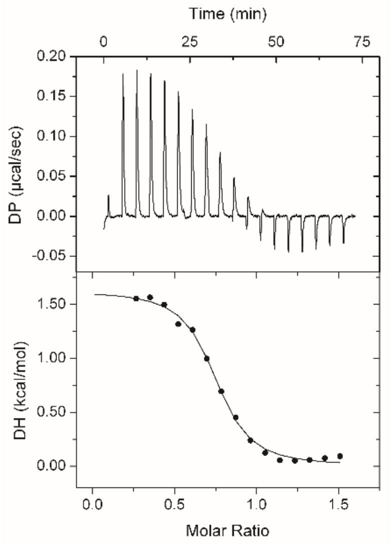

pH 10.0*

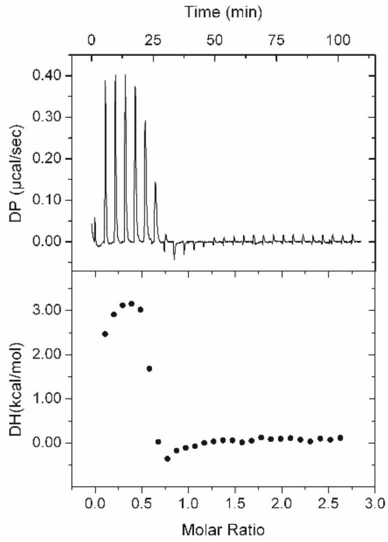

pH 11.0*

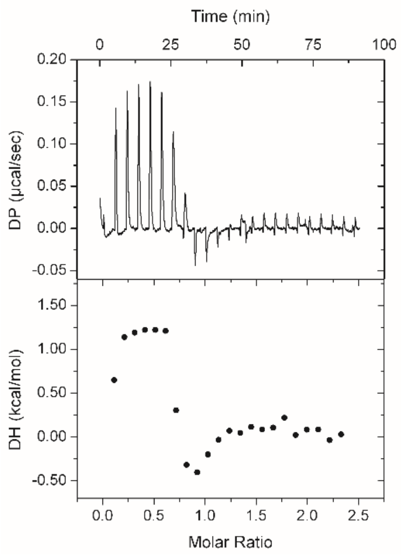

* Data could not be analysed in a satisfactory manner. There was a rapid transition from binding to saturation, indicative of a high-affinity binding. This binding was therefore defined as K_D below 1 µM.

L-ornithine recognition by E6B08_RS28125

pH 3.0

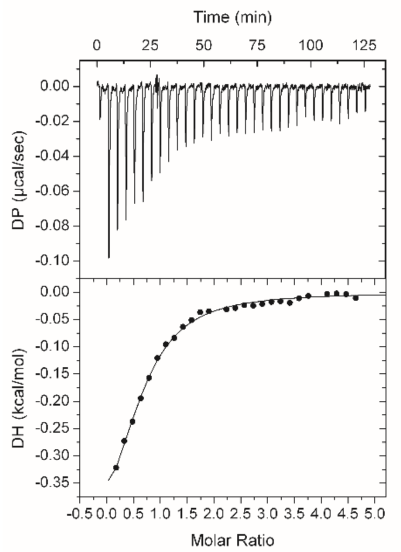

pH 4.0

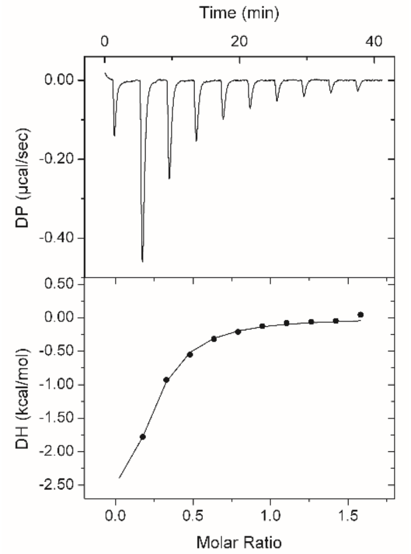

pH 5.0

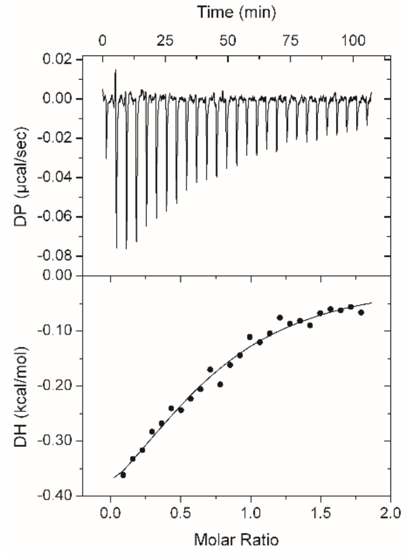

pH 6.0

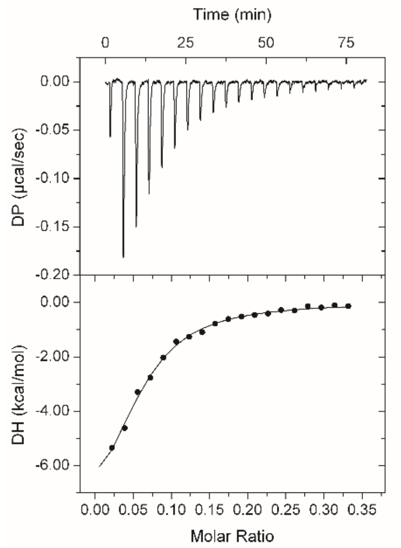

pH 7.0

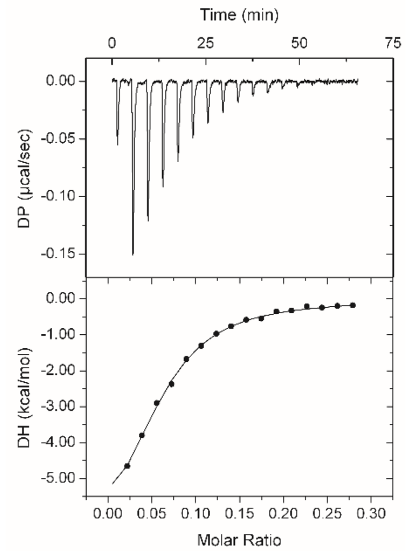

pH 8.0

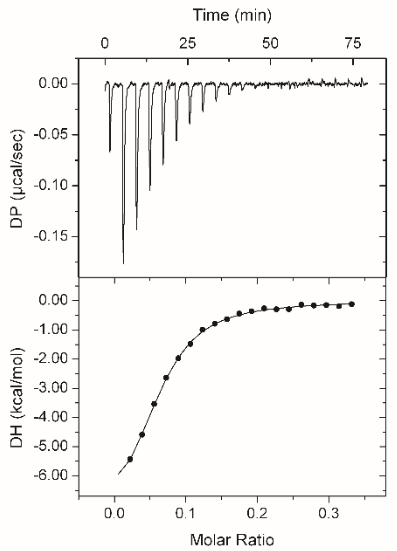

pH 9.0

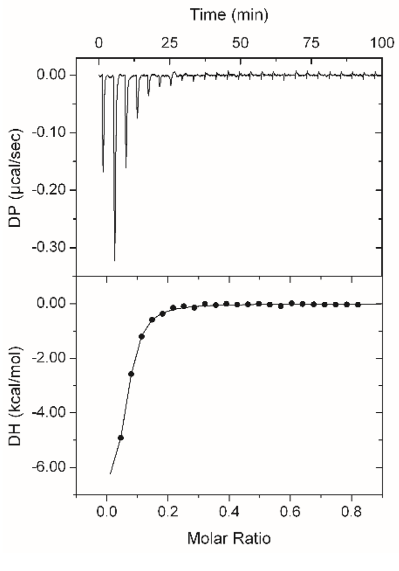

pH 10.0

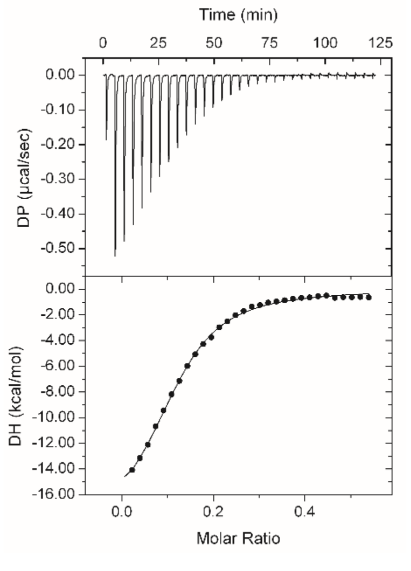

pH 11.0

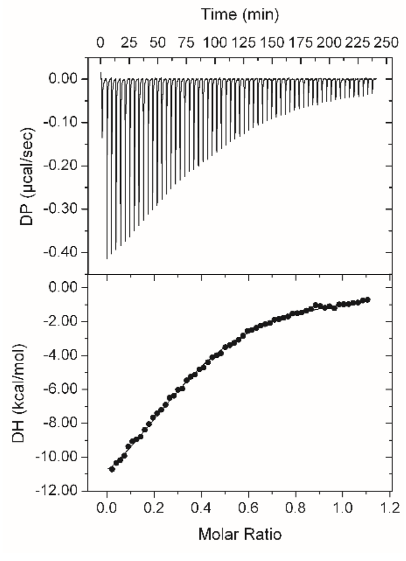

Toluene recognition by TodS-Nter

pH 5.5

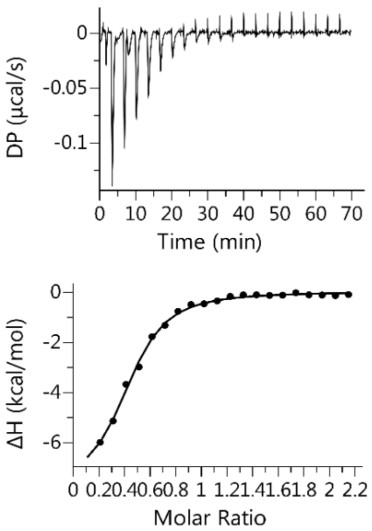

pH 6.0

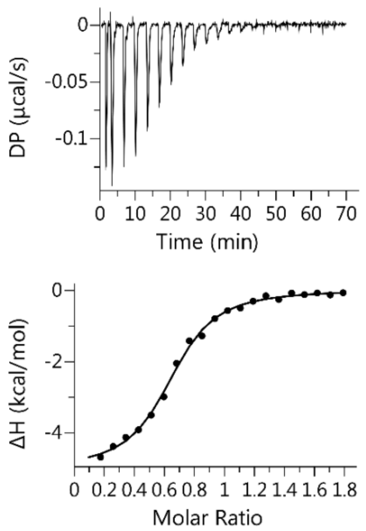

pH 6.5

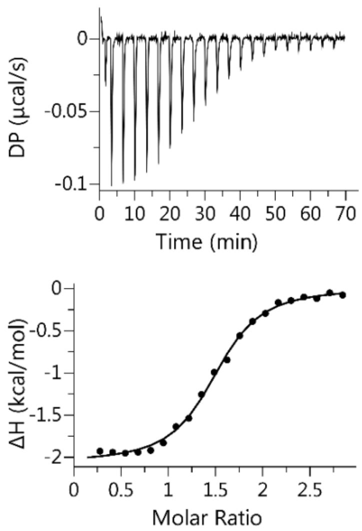

pH 7.0

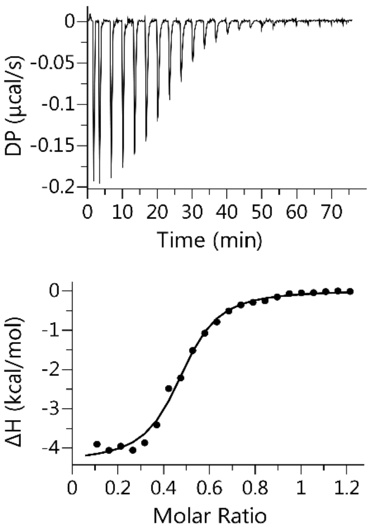

pH 7.5

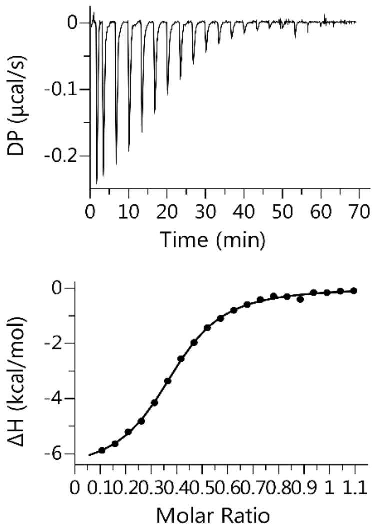

pH 8.0

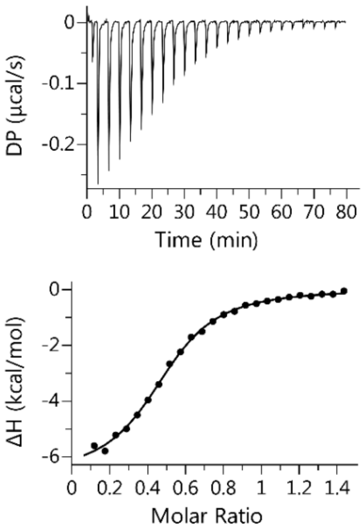

pH 9.0

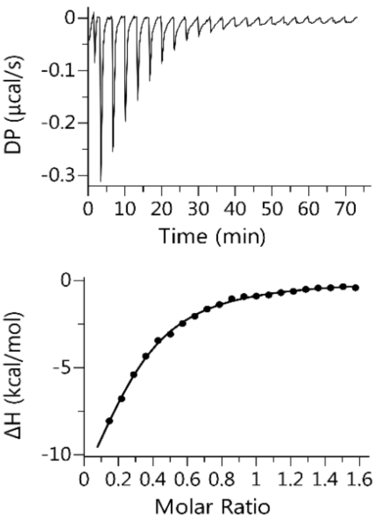

pH 9.5

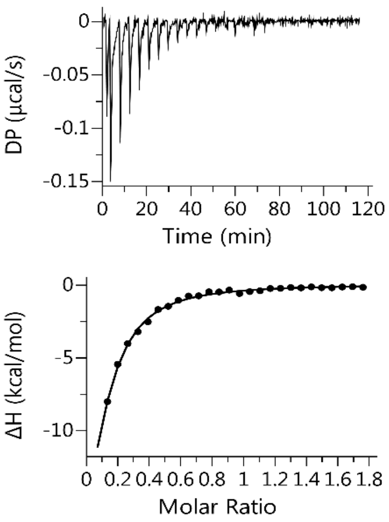

Indole-3-pyruvic acid recognition by AdmX-LBD

pH 7.5	pH 8.0
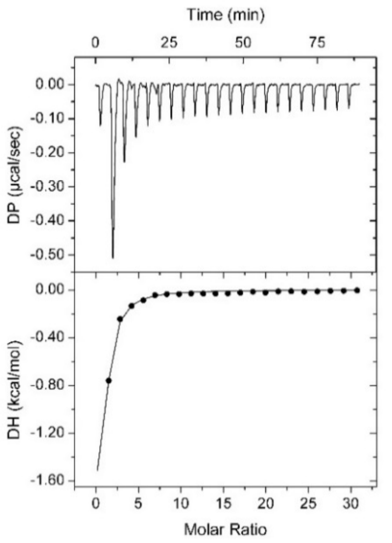	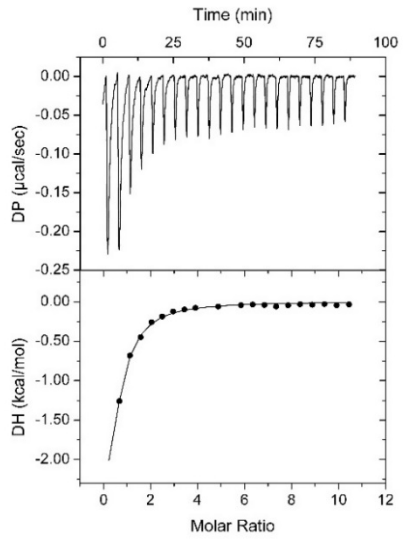

Benzonitrile recognition by TtgV

pH 5.0	pH 6.0	pH 7.0
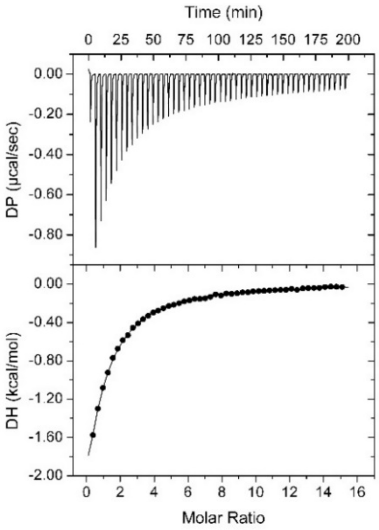	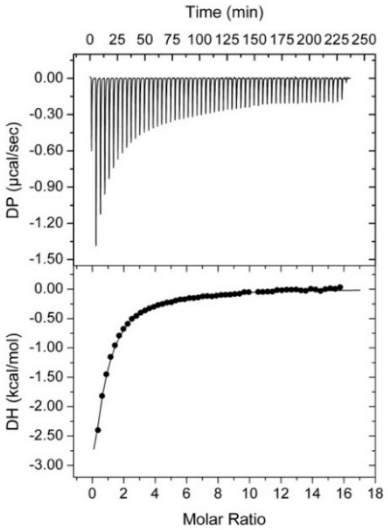	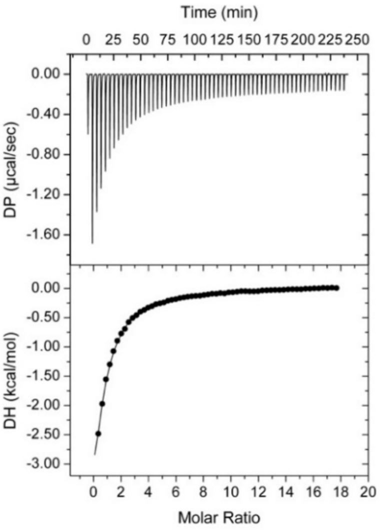
pH 8.0	pH 9.0	
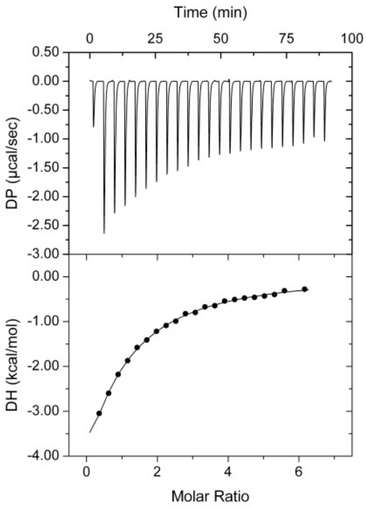	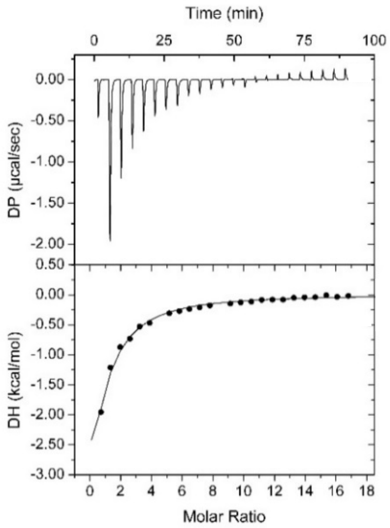	
