# Supplementary figures and images for: The pH Robustness of Bacterial Sensing
Source: mBio. 2022 Sep 26;13(5):e01650-22. doi: 10.1128/mbio.01650-22 (PMC9600550; doi:10.1128/mbio.01650-22)

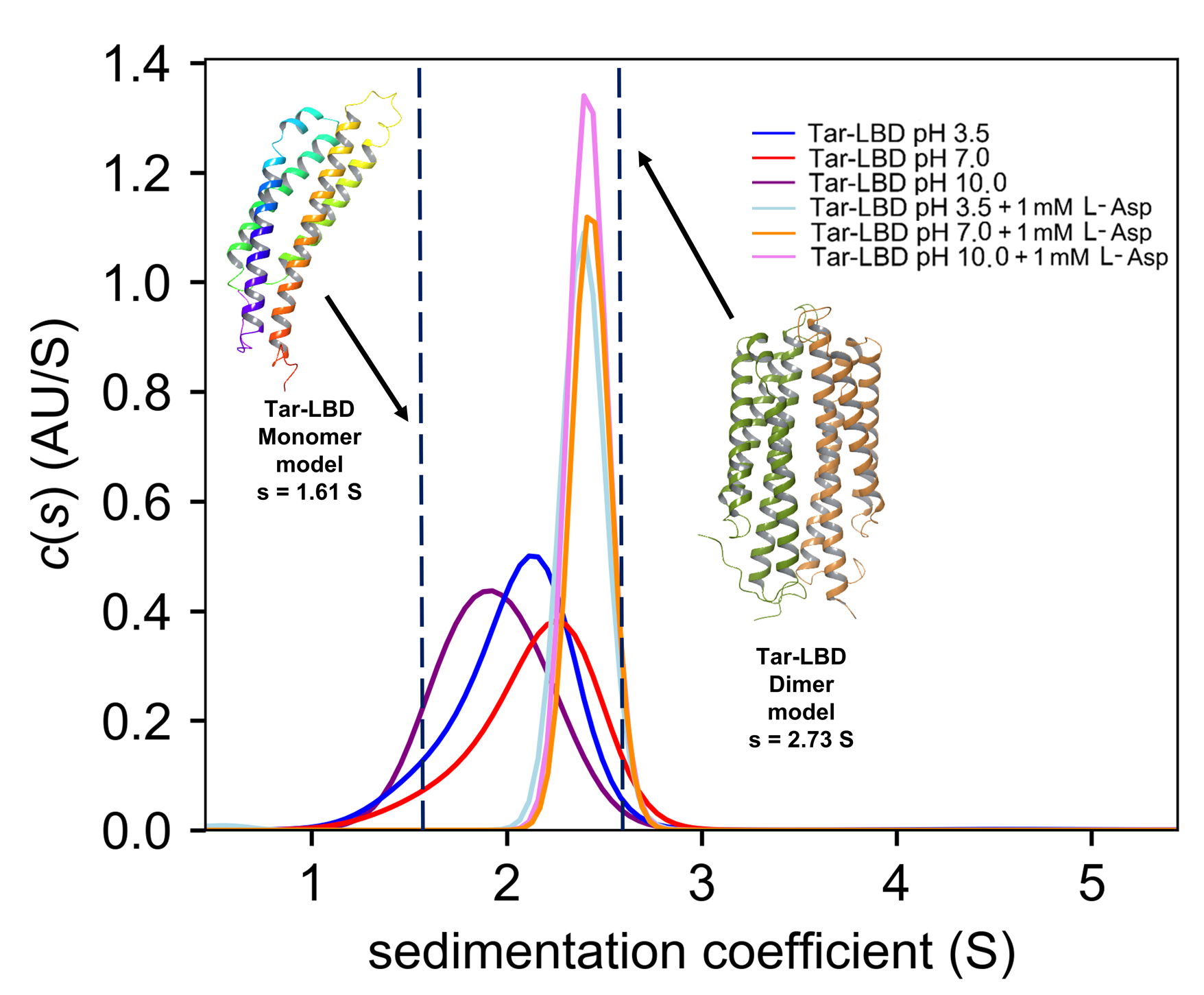

Supplement: FIG S4 [file mbio.01650-22-s0008.tif]

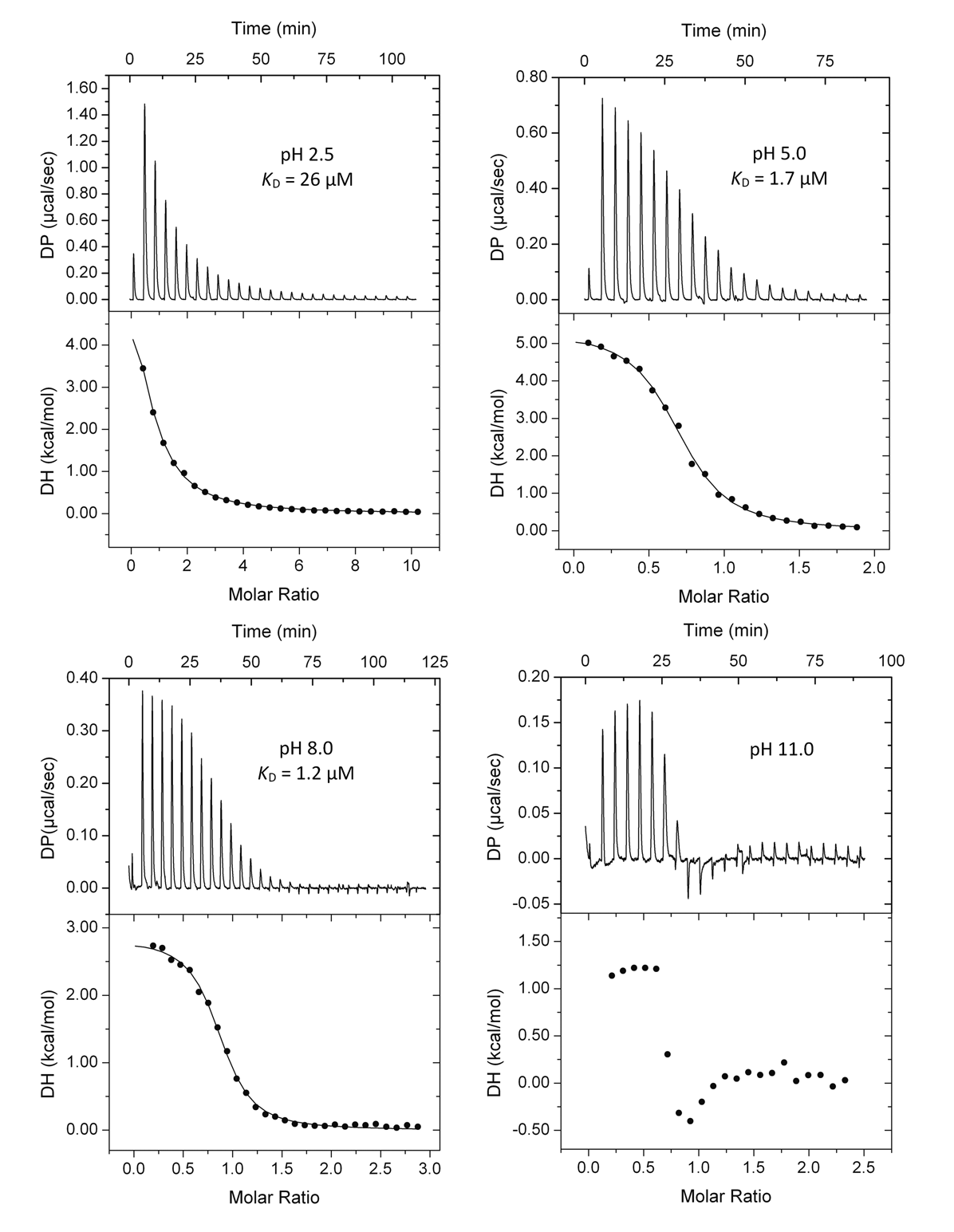

Supplement: FIG S5 [file mbio.01650-22-s0009.tif]

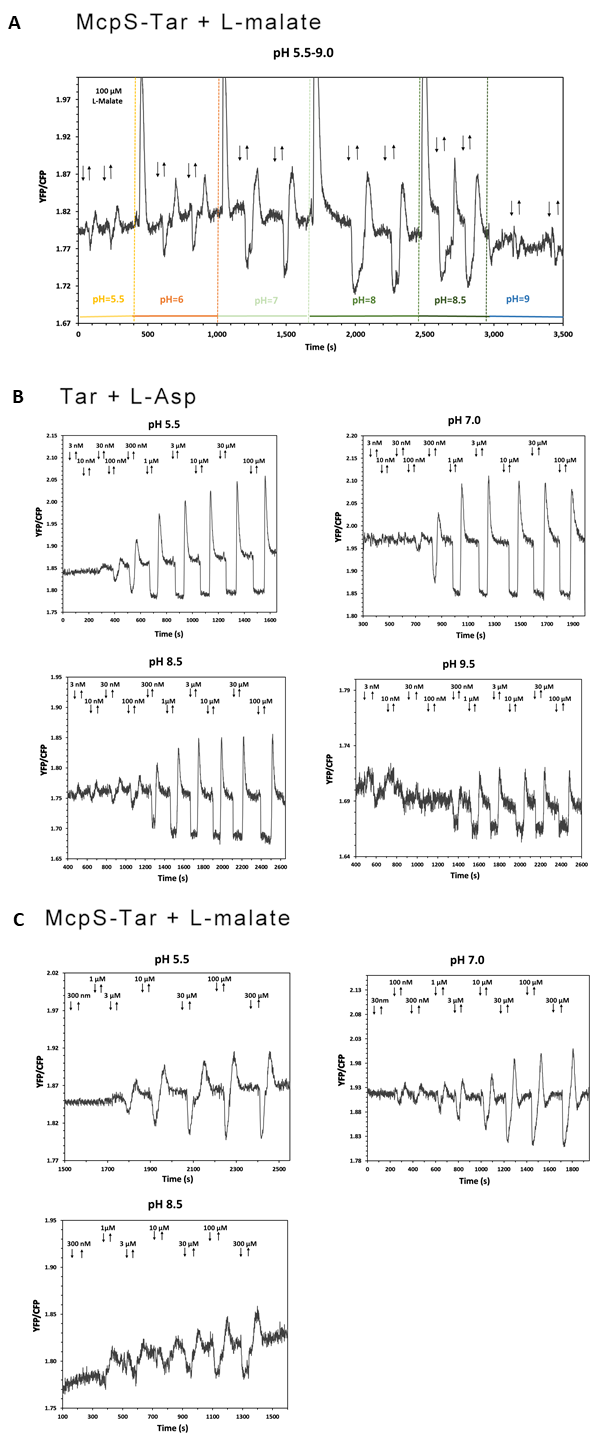

Supplement: FIG S6 [file mbio.01650-22-s0010.tif]
