# Supplementary material for: The pH Robustness of Bacterial Sensing
Source: mBio. 2022 Sep 26;13(5):e01650-22. doi: 10.1128/mbio.01650-22 (PMC9600550; doi:10.1128/mbio.01650-22)
Supplement: TABLE S3 [file mbio.01650-22-s0003.pdf]

Table S3) Thermodynamic parameters for the thermal unfolding of Tar-LBD at different pH in the absence and presence of 1 mM L-Asp.

pH	Ligand	Tm (ºC)	Δ*H* (kcal/mol)
3.5	-	44.16<br>66.17	157<br>34.8
5.0	-	41.71	88.4
7.0	-	32.65	53.0
10.0	-	No unfolding events	
3.5	1 mM L-Asp	44.28<br>64.51	184<br>8.7
5.0	1 mM L-Asp	47.73	106.4
7.0	1 mM L-Asp	36.48<br>42.20	79.3<br>26.2
10.0	1 mM L-Asp	35.11	98.6
