# Supplementary material for: The pH Robustness of Bacterial Sensing
Source: mBio. 2022 Sep 26;13(5):e01650-22. doi: 10.1128/mbio.01650-22 (PMC9600550; doi:10.1128/mbio.01650-22)
Supplement: TABLE S4 [file mbio.01650-22-s0004.pdf]

Table S4) Strains, plasmids and oligonucleotides used in this study.

Strains and plasmids	Genotype or relevant characteristics[a]	Reference
**Strains**		
Escherichia coli BL21(DE3)	F^- *ompT gal dcm lon* $hsdS_B(r_B^- m_B^-)$ λ(DE3 [*lacI lacUV5-T7p07 ind1 sam7 nin5*]) [$malB^+$]$_{K-12}$($λ^S$)	(1)
E. coli DH5α	F^- *endA1 glnV44 thi-1 recA1 relA1 gyrA96 deoR nupG purB20* φ80d*lacZ*ΔM15 Δ(*lacZYA-argF*)U169, hsdR17($r_K^- m_K^+$), $λ^-$	(2)
E. coli VS181	*E. coli* RP437; Δ(*cheYcheZ*)Δ*aer*Δ*tsr*Δ(*tar-tap*) Δ*trg*	(3)
**Plasmids**		
pET28b(+)	Protein expression plasmid; Km^R	Novagen
pET28-PctA-LBR	Km^R; pET28b(+) derivative containing a DNA fragment encoding the PctA-LBD	(4)
pET28_LBD_McpH	Km^R; pET28b(+) derivative containing a DNA fragment encoding the McpH-LBD	(5)
pET28b-LBDMcpU	Km^R; pET28b(+) derivative containing a DNA fragment encoding the McpU-LBD	(6)
pET28b-PA2652LBD	Km^R; pET28b(+) derivative containing a DNA fragment encoding the CtpM (PA2652)-LBD	(7)
pBS377	Ap^R, pQE60 derivative containing a DNA fragment encoding the McpV-LBD	(8)
pET28b-McpV-LBD	Km^R; pET28b(+) derivative containing a DNA fragment encoding the McpV-LBD	This study
pET28-Tar-LBD	Km^R; pET28b(+) derivative containing a DNA fragment encoding the Tar-LBD	This study
pET28-PcaY_PP-LBD	Km^R; pET28b(+) derivative containing a DNA fragment encoding the PcaY_PP-LBD	(9)
pETMcpS	Km^R; pET28b(+) derivative containing a DNA fragment encoding the McpS-LBD	(10)
pET28b-McpQ-LBD	Km^R; pET28b(+) derivative containing a DNA fragment encoding the McpQ-LBD	(11)
pET28-MBP	Km^R; pET28b(+) derivative containing a DNA fragment encoding the *E. coli* MBP	This study
pMAMV385	Km^R; pET28b(+) derivative containing a DNA fragment encoding the E6B08_RS28125	(12)
pNTodS	Km^R; pET28b(+) derivative containing a DNA fragment encoding the PAS1 and autokinase1 domain of TodS	(13)
pMAMV235	Km^R; pET28b(+) derivative containing a DNA fragment encoding AdmX-LBD	(14)
pANA126	Km^R; pET28b(+) derivative containing a DNA fragment encoding TtgV	(15)
pVS88	Ap^R ; CheY-EYFP / CheZ-ECFP expression plasmid	(3)
pKG116	Protein expression vector; Cm^R	(16)
pSB13	Cm^R; pKG116 derivative encoding Tar receptor, with T->C768 nucleotide substitution to remove the *Nde*I restriction site	(17)
pSB10	Cam^R; pKG116 derivative encoding hybrid McpS[1-	(18)

	295]-Tar[200-553] receptor	

Oligonucleotides

Name	Sequence (5’-3’)	Purpose
McpV-LBD-f	ATCCATATGCAGGACAAGCTGGTTGCCG	Construction of pET28b-McpV-LBD
McpV-LBD-f	TATAAGCTTTTACTGCCAGGCGCGCTCGC	
Tar-LBD-f	TAATCATATGTCTTCCCTTCACCATAGCCAG	Construction of pET28-Tar-LBD
Tar-LBD-r	TAATGGATCCTCATCGGTAATCATCTGCGTTG	
MBP-f	GGAATTCCATATGAAAATAAAAACAGGTGCACG	Construction of pET28-MBP
MBP-r	CCCGCTCGAGTTACTTGGTGATACGAGTCTGCG	

[a]Ap, ampicillin; Km, kanamycin; Tc, tetracycline. Cm, chloramphenicol.

References

1. Jeong, H., Barbe, V., Lee, C. H., Vallenet, D., Yu, D. S., Choi, S. H., Couloux, A., Lee, S. W., Yoon, S. H., Cattolico, L., Hur, C. G., Park, H. S., Segurens, B., Kim, S. C., Oh, T. K., Lenski, R. E., Studier, F. W., Daegelen, P. & Kim, J. F. (2009). Genome sequences of *Escherichia coli* B strains REL606 and BL21(DE3). *J Mol Biol.* 394, 644-652.
2. Woodcock, D. M., Crowther, P. J., Doherty, J., Jefferson, S., DeCruz, E., Noyer-Weidner, M., Smith, S. S., Michael, M. Z. & Graham, M. W. (1989). Quantitative evaluation of *Escherichia coli* host strains for tolerance to cytosine methylation in plasmid and phage recombinants. *Nucleic Acids Res.* 17, 3469-3478.
3. Sourjik, V. & Berg, H. C. (2004). Functional interactions between receptors in bacterial chemotaxis. *Nature.* 428, 437-441.
4. Rico-Jimenez, M., Munoz-Martinez, F., Garcia-Fontana, C., Fernandez, M., Morel, B., Ortega, A., Ramos, J. L. & Krell, T. (2013). Paralogous chemoreceptors mediate chemotaxis towards protein amino acids and the non-protein amino acid gamma-aminobutyrate (GABA). *Mol Microbiol.* 88, 1230-1243.
5. Fernandez, M., Morel, B., Corral-Lugo, A. & Krell, T. (2016). Identification of a chemoreceptor that specifically mediates chemotaxis toward metabolizable purine derivatives. *Mol Microbiol.* 99, 34-42.
6. Corral-Lugo, A., de la Torre, J., Matilla, M. A., Fernandez, M., Morel, B., Espinosa-Urgel, M. & Krell, T. (2016). Assessment of the contribution of chemoreceptor-based signaling to biofilm formation. *Environ Microbiol.* 18, 3355-3372.
7. Martin-Mora, D., Ortega, A., Perez-Maldonado, F. J., Krell, T. & Matilla, M. A. (2018). The activity of the C4-dicarboxylic acid chemoreceptor of *Pseudomonas aeruginosa* is controlled by chemoattractants and antagonists. *Sci Rep.* 8, 2102.
8. Compton, K. K., Hildreth, S. B., Helm, R. F. & Scharf, B. E. (2018). Sinorhizobium meliloti Chemoreceptor McpV Senses Short-Chain Carboxylates via Direct Binding. *J Bacteriol.* 200
9. Fernandez, M., Matilla, M. A., Ortega, A. & Krell, T. (2017). Metabolic Value Chemoattractants Are Preferentially Recognized at Broad Ligand Range Chemoreceptor of *Pseudomonas putida* KT2440. *Front Microbiol.* 8, 990.
10. Lacal, J., Alfonso, C., Liu, X., Parales, R. E., Morel, B., Conejero-Lara, F., Rivas, G., Duque, E., Ramos, J. L. & Krell, T. (2010). Identification of a chemoreceptor for tricarboxylic acid cycle intermediates: differential chemotactic response towards receptor ligands. *J Biol Chem.* 285, 23126-23136.
11. Martin-Mora, D., Reyes-Darias, J. A., Ortega, A., Corral-Lugo, A., Matilla, M. A. & Krell, T. (2016). McpQ is a specific citrate chemoreceptor that responds preferentially to citrate/metal ion complexes. *Environ Microbiol.* 18, 3284-3295.

12. Rico-Jimenez, M., Krell, T. & Matilla, M. A. (2022). A bacterial chemoreceptor that mediates chemotaxis to multiple plant hormones. *(submitted).*
13. Lacal, J., Busch, A., Guazzaroni, M. E., Krell, T. & Ramos, J. L. (2006). The TodS-TodT two-component regulatory system recognizes a wide range of effectors and works with DNA-bending proteins. *Proc Natl Acad Sci U S A.* 103, 8191-8196.
14. Matilla, M. A., Daddaoua, A., Chini, A., Morel, B. & Krell, T. (2018). An auxin controls bacterial antibiotics production. *Nucleic Acids Res.* 46, 11229-11238.
15. Rojas, A., Segura, A., Guazzaroni, M. E., Terán, W., Hurtado, A., Gallegos, M. T. & Ramos, J. L. (2003). In vivo and in vitro evidence that TtgV is the specific regulator of the TtgGHI multidrug and solvent efflux pump of Pseudomonas putida. *J Bacteriol.* 185, 4755-4763.
16. Buron-Barral, M. C., Gosink, K. K. & Parkinson, J. S. (2006). Loss- and gain-of-function mutations in the F1-HAMP region of the Escherichia coli aerotaxis transducer Aer. *J Bacteriol.* 188, 3477-3486.
17. Bi, S., Jin, F. & Sourjik, V. (2018). Inverted signaling by bacterial chemotaxis receptors. *Nat Commun.* 9, 2927.
18. Bi, S., Pollard, A. M., Yang, Y., Jin, F. & Sourjik, V. (2016). Engineering Hybrid Chemotaxis Receptors in Bacteria. *ACS Synth Biol.* 5, 989-1001.
